# Regioselective synthesis of methyl 5-(*N*-Boc-cycloaminyl)-1,2-oxazole-4-carboxylates as new amino acid-like building blocks

**DOI:** 10.3762/bjoc.18.11

**Published:** 2022-01-12

**Authors:** Jolita Bruzgulienė, Greta Račkauskienė, Aurimas Bieliauskas, Vaida Milišiūnaitė, Miglė Dagilienė, Gita Matulevičiūtė, Vytas Martynaitis, Sonata Krikštolaitytė, Frank A Sløk, Algirdas Šačkus

**Affiliations:** 1 Institute of Synthetic Chemistry, Kaunas University of Technology, K. Baršausko g. 59, Kaunas LT-51423, Lithuania; 2 Department of Organic Chemistry, Kaunas University of Technology, Radvilėnų pl. 19, Kaunas LT-50254, Lithuania; 3 Vipergen ApS, Gammel Kongevej 23A, DK-1610 Copenhagen V, Denmark

**Keywords:** β-enamino ketoesters, heterocyclic amino acids, ^15^N-labeled 1,2-oxazole, NMR (^1^H, ^13^C, ^15^N), 1,2-oxazole (isoxazole), X-ray structure analysis

## Abstract

A convenient and efficient synthesis of novel achiral and chiral heterocyclic amino acid-like building blocks was developed. Regioisomeric methyl 5-(*N*-Boc-cycloaminyl)-1,2-oxazole-4-carboxylates were prepared by the reaction of β-enamino ketoesters (including azetidine, pyrrolidine or piperidine enamines) with hydroxylamine hydrochloride. Unambiguous structural assignments were based on chiral HPLC analysis, ^1^H, ^13^C, and ^15^N NMR spectroscopy, HRMS, and single-crystal X-ray diffraction data.

## Introduction

**Figure 1:**
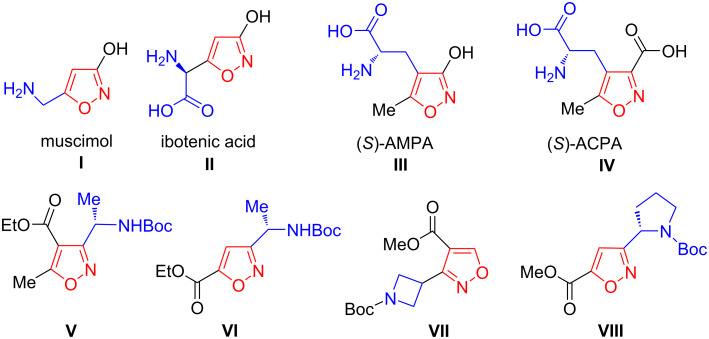
Examples of amino-functionalized 1,2-oxazole derivatives **I**–**VIII**.

1,2-Oxazoles (isoxazoles) constitute an important class of heterocyclic compounds that plays a fundamental role in drug discovery [1–7]. Many amino-functionalized 1,2-oxazole derivatives are biologically active substances that include naturally occurring and synthetic neuroactive compounds. Specifically, natural products such as muscimol (**I**) and ibotenic acid (**II**) (Figure 1) have been isolated from several fungal species and are active on the γ-aminobutyric acid (GABA) and glutamate receptors of the central nervous system (CNS), respectively [8–9]. Various unnatural amino acids bearing a 1,2-oxazole moiety, such as nonproteinogenic α-amino acids, have been used as excitatory amino acid receptor agonists [10–13]. For example, (*S*)-AMPA (**III**) and (*S*)-ACPA (**IV**) are specific agonists of an AMPA receptor that mimic the effects of the neurotransmitter glutamate [14–16].

Unnatural heteroarene amino acids have also been widely used as building blocks to prepare various heterocyclic peptides [17–22]. In particular, 1,2-oxazole amino acid derivatives, such as compounds **V** [20], **VI** [21], and **VII, VIII** [22], can be easily synthesized and are suitable for insertion with the corresponding heterocycle into a peptide-like structure.

Heterocyclic amino acids and related compounds have been used to prepare synthetic DNA-encoded compound libraries for the discovery of small molecule protein ligands [23–25]. Recently, a highly specific and potent p38α kinase inhibitor containing a 3-amino-1-phenyl-1*H*-pyrazole-4-carboxylic acid residue was identified directly from the 12.6-million-member DNA-encoded small molecule library using yoctoReactor technology [26]. We have developed efficient protocols that provide easy access to highly functional heterocyclic compounds as novel amino acid-like building blocks by combining thiazole, selenazole, pyrazole, indazole, and indole moieties with both carboxyl functional groups and cycloaminyl units [27–31].

In recent decades, various methods of constructing 1,2-oxazole ring systems have been developed [1–7,32]. The two primary pathways to 1,2-oxazoles are: the 1,3-dipolar cycloaddition of alkenes and alkynes with nitrile oxides, and the reaction of a three-carbon atom component, such as a α,β-unsaturated ketone or a 1,3-diketone with hydroxylamine hydrochloride [33]. Recently, Rosa et al. reported a useful procedure for the synthesis of various regioisomeric 1,2-oxazole derivatives. Accordingly, the synthetic route starts from the condensation of 1,3-diketones with *N*,*N*-dimethylformamide dimethylacetal to form β-enamino ketoester. The latter undergoes a subsequent cycloaddition reaction with hydroxylamine to form regioisomerically substituted 1,2-oxazoles [34–35].

This study aimed to develop and synthesize methyl 5-(cycloaminyl)-1,2-oxazole-4-carboxylates, as new amino acid-like building blocks. This type of functionalized heterocycles could exhibit not only useful biological properties, but also find application as building blocks for the generation of DNA-encoded chemical libraries.

## Results and Discussion

**Scheme 1:**
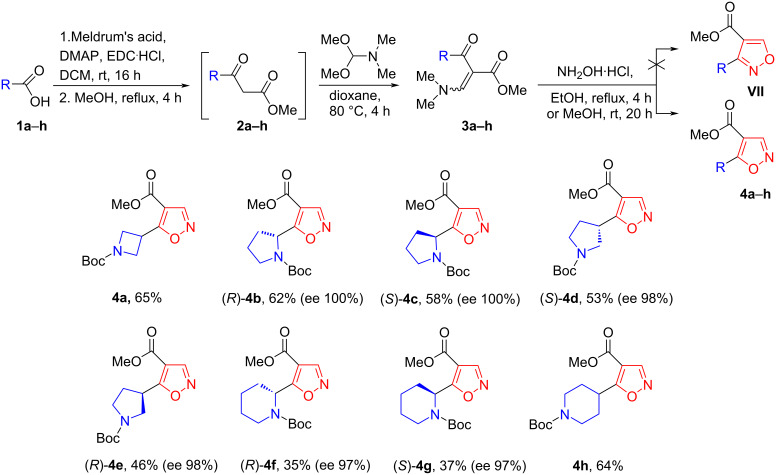
Conversion of cyclic amino acids to 1,2-oxazole derivatives.

**Figure 2:**
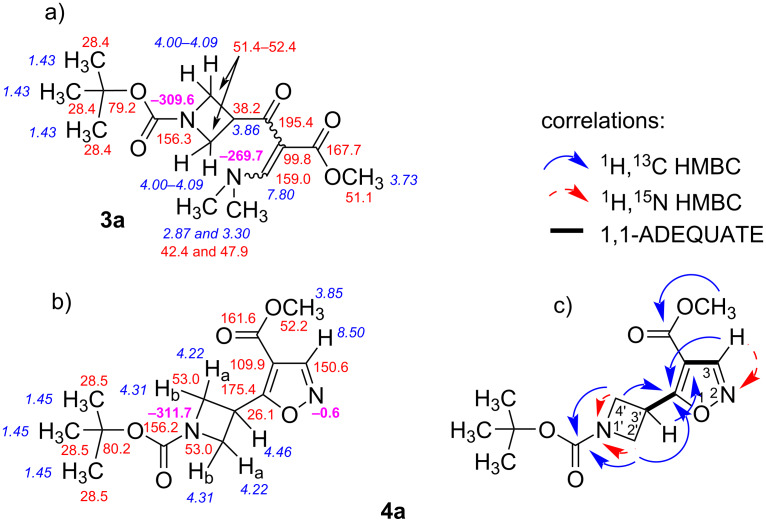
(a) ^1^H NMR (italics), ^13^C NMR (normal), and ^15^N NMR (bold) chemical shifts (ppm) of compound **3a** in CDCl_3_; (b) ^1^H NMR (italics), ^13^C NMR (normal), and ^15^N NMR (bold) chemical shifts (ppm) of compound **4a** in CDCl_3_; (c) relevant ^1^H,^13^C HMBC, ^1^H,^15^N HMBC and 1,1-ADEQUATE correlations of compound **4a**.

The synthetic strategy for the synthesis of novel functionalized 1,2-oxazole derivatives is outlined in Scheme 1. The synthetic sequence began with preparing β-keto esters **2a**–**h** by treating *N*-Boc-protected cyclic amino acids **1a**–**h** with Meldrum’s acid in the presence of EDC·HCl and DMAP, followed by methanolysis of the corresponding adducts [27–28,31,36–38]. Reaction of the resulting β-keto esters **2a**–**h** with *N*,*N*-dimethylformamide dimethylacetal afforded cycloaminyl β-enamino ketoesters **3a**–**h**. After isolation of compounds **3a**–**h** from the corresponding reaction mixtures, they were identified using LC–MS analysis, and were immediately treated with hydroxylamine hydrochloride in an appropriate solvent to obtain the target 1,2-oxazoles **4a**–**h**. A representative β-enamino ketoester **3a** was subjected to a detailed NMR analysis (Figure 2a). The ^1^H NMR spectrum of compound **3a** showed the appearance of a new downfield enamine proton signal which resonated at δ 7.80 ppm. The connectivity of the β-enamino ketoester moiety and the *N*-Boc-protected azetidine fragment were easily confirmed based on long-range ^1^H,^13^C correlations, obtained from gs-HMBC spectra. The aforementioned enamine proton and protons 2’(4’)-H (δ 4.00-4.09 ppm) from the azetidine ring system shared the HMBC cross-peak with the ketone carbonyl carbon (δ 195.4 ppm). Finally, in the ^1^H,^15^N-HMBC spectrum of **3a**, an expected long-range correlation between the enamine proton (δ 7.80 ppm) and the dimethylamino nitrogen (δ −269.7 ppm) was observed, thus allowing to prove the formation of β-enamino ketoester.

**Scheme 2:**
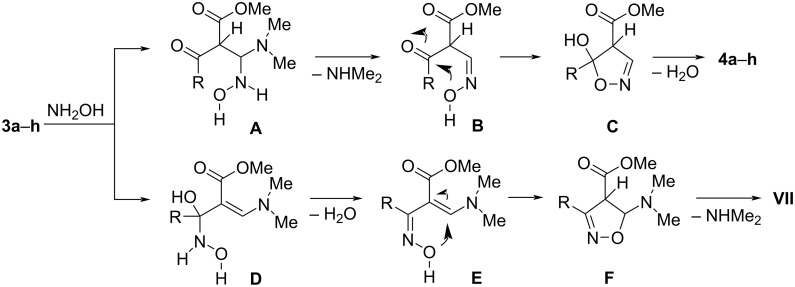
Plausible mechanisms for the formation of 1,2-oxazoles **4a**–**h** and **VII** from β-enamino ketoesters **3a**–**h** with hydroxylamine.

Analysis of the possible resulting reaction showed that the condensation reaction between β-enamino ketoester precursors **3a–h** and hydroxylamine could lead to the formation of two isomeric 1,2-oxazoles (Scheme 2) [34–35]. In the first route, enaminone **3a**–**h** and hydroxylamine gives intermediate **A**, which then removes one molecule of dimethylamine to form intermediate **B**. This is followed by intramolecular cyclization to intermediate **C**, and subsequent dehydration to generate the final products **4a**–**h**. Alternatively, nucleophilic attack of hydroxylamine to the carbonyl carbon atom of the enone moiety forms intermediate **D**. The dehydration of the latter provides oxime **E**, which through the formation of intermediate **F**, should be capable to form final product **VII**. However, as demonstrated with substrate **3a**, the reaction only gave product **4a** with a yield of 65% (Scheme 1). LC–MS analysis and flash column chromatography did not allow to detect the formation of the second possible isomeric product methyl 3-(*N*-Boc-azetidin-3-yl)-1,2-oxazole-4-carboxylate (**VII**) (Figure 1) [22].

The structural assignment of regiospecific compound **4a** was readily deduced via detailed spectral data analysis. The IR spectrum of **4a** contained characteristic absorption bands such as 1723 (C=O, ester), and 1687 (C=O, Boc) cm^−1^. The ^1^H NMR spectrum of compound **4a** revealed a characteristic resonance for the Boc group protons, a singlet in the δ 1.45 ppm region, while the signal of the protons of the COOMe group appeared as a singlet at approximately δ 3.85 ppm (Figure 2b). The azetidine methylene protons (CH_a_H_b_-2’ and CH_a_H_b_-4’) exhibited a doublet of doublets at δ 4.24 ppm (*J* = 8.7, 6.5 Hz) and a triplet at δ 4.32 ppm (*J* = 8.8 Hz), while the methine proton (3’-H) yielded a triplet of triplets at δ 4.48 ppm (*J* = 8.9, 6.5 Hz). A comparison between the DEPT-90, DEPT-135 and ^13^C NMR spectra of compound **4a** clearly indicated the presence of methine carbons C-3 (δ 150.6 ppm) and C-3’ (δ 26.1 ppm), respectively. The ^1^H,^13^C-HMBC spectrum of compound **4a** revealed that the methylene protons from the azetidine moiety and the 1,2-oxazole methine proton H-3, exhibited long-range correlations with quaternary carbon C-5 (Figure 2c). The aforementioned protonated carbons C-3 and C-3’ showed correlations in the 1,1-ADEQUATE spectrum thus allowing to assign a neighboring quaternary carbons C-4 (δ 109.9 ppm) and C-5 (δ 175.4 ppm), H–C(3)–C(4) and H–C(3’)–C(5), respectively. The ^1^H,^15^N-HMBC experiment revealed an expected long-range correlation between the 1,2-oxazole methine H-3 proton (δ 8.50 ppm) and nitrogen N-2 which resonated at δ −0.6 ppm, while the azetidine ring protons showed a sole correlation with the azetidine nitrogen N-1’ (δ −311.7 ppm) [29,39].

**Scheme 3:**
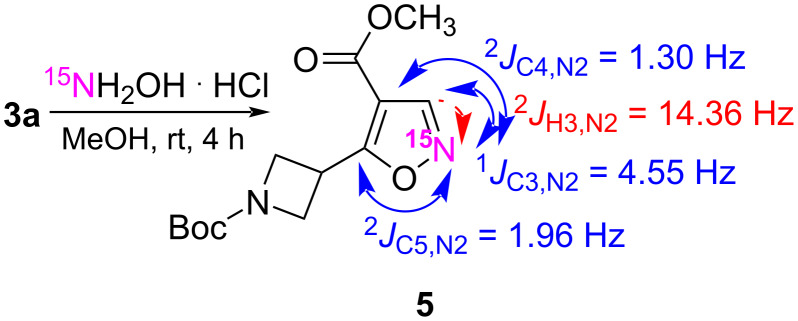
Synthesis of compound ^15^N-1,2-oxazole **5**. The coupling constants of *J*_HN_ and *J*_CN_ from ^15^N2 are indicated by arrows.

Furthermore, ^15^N-labeled methyl 5-(*N*-Boc-azetidin-3-yl)-1,2-oxazole-4-carboxylate (**5**) was synthesized by analogy to **4a**, by the reaction of β-enamino ketoester **3a** with ^15^N-hydroxylamine hydrochloride (Scheme 3). Incorporation of a ^15^N atom in azaheterocycles is an important method for studying molecular structures, which significantly expands the possibilities of using NMR methods [40]. The ^15^N-labeled aromatic heterocyclic structures usually have well-resolved ^1^H,^15^N (*J*_HN_) and ^13^C,^15^N (*J*_CN_) coupling constants, as well as additional splitting of the corresponding signals in the standard proton decoupled 1D ^13^C NMR and 1D ^1^H NMR spectra [41–42]. The ^1^H,^15^N coupling constants in the azoles, especially the rather large ^2^*J*_HN_ values, which are in the range of 13–15 Hz is widely accepted in the structure assignments and are even considered as diagnostic for this class of compounds, because the ^3^*J*_HN_ is typically in the range of 1–3 Hz. For example, the ^1^H,^15^N coupling constants were measured for a series of 1,2-oxazoles, which provided the ^2^*J*_HN_ values in the range of 14.4–14.7 Hz, while the ^3^*J*_HN_ values were in the range of 1–3 Hz [42–43]. The coupling constants ^13^C,^15^N of a series of ^15^N-labeled pyrazoles gave ^1^*J*_CN_ values of 8–11 Hz, while ^2^*J*_CN_ were less than 2 Hz [44]. The ^15^N-labeled 1,2-thiazole moiety was readily determined by measuring the corresponding direct ^13^C,^15^N coupling constant ^1^*J*_C3-N2_ of 6.9 Hz [44].

Therefore, the ^1^H NMR spectrum of ^15^N-labeled methyl 5-(*N*-Boc-azetidin-3-yl)-1,2-oxazole-4-carboxylate (**5**) showed a ^1^H,^15^N coupling constant (^2^*J*_H3–N2_ = 14.36 Hz)**,** while in the ^13^C NMR spectrum, the ^13^C,^15^N interaction was observed for signals C-3 (^1^*J*_C3-N2_ = 4.55 Hz), C-4 (^2^*J*_C4-N2_ = 1.30 Hz), and C-5 (^2^*J*_C5-N2_ = 1.96 Hz), which unambiguously indicates the presence of a 1,2-oxazole ring in the target compound.

**Figure 3:**
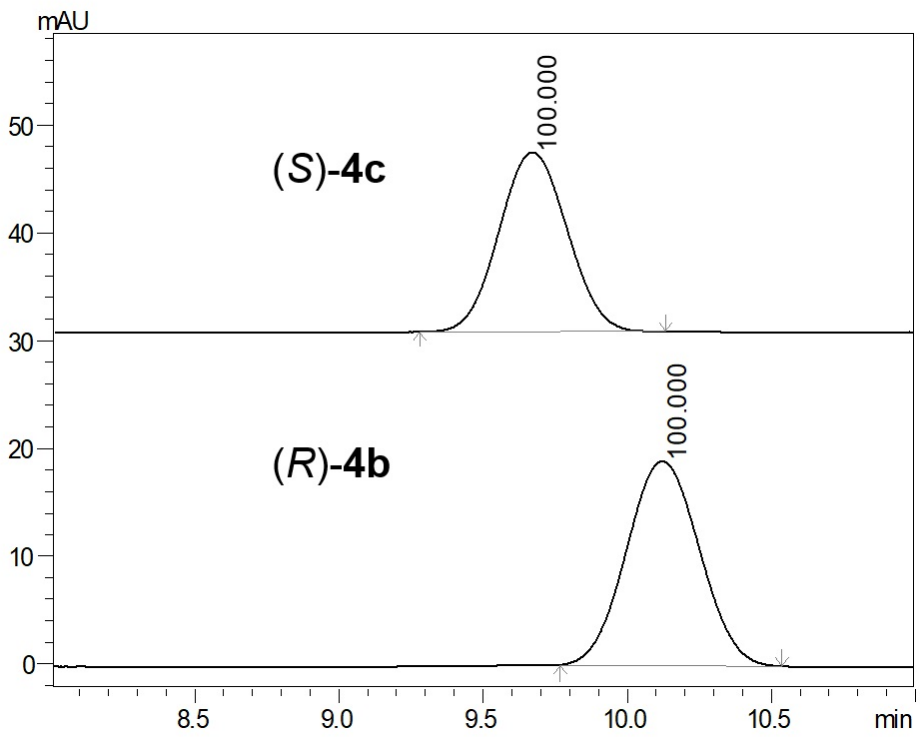
Stacked chromatogram view of pairs of enantiomers with area, %: (*R*)-**4b**, ee 100% (*t*_R_ = 10.1 min) and (*S*)-**4c**, ee 100% (*t*_R_ = 9.6 min); conditions: CHIRAL ART Cellulose-SB (100 × 4.6 mm I.D., S-3 µm, chiral selector cellulose tris(3,5-dimethylphenylcarbamate), YMC); mobile phase: ACN/(H_2_O + 0.1% HCOOH (30:70 isocratic mode); *T* = 36 °C; flow rate 1.0 mL/min. Samples were prepared in methanol. The injection volume was 10 μL, λ_det_ = 245 nm.

Also, chiral methyl 5-(*N*-Boc-cycloaminyl)-1,2-oxazole-4-carboxylates **4b**–**g** were obtained from β-enamino ketoesters **3b**–**g** with hydroxylamine hydrochloride in methanol (Scheme 1). Synthesized compounds **4b**–**g** exhibited optical activity, and the corresponding (*R*)- or (*S*)-enantiomers rotated the plane of plane-polarized light in opposite directions. The enantiomeric purity of chiral compounds **4b**–**g** was assessed via chiral HPLC analysis of enantiomeric samples, as shown in Figure 3 (**4b**,**c**, ee 100%), Supporting Information File 1, Figure S51 (**4d**,**e**, ee 98%) and Supporting Information File 1, Figure S52 (**4f**,**g**, ee 97%).

**Figure 4:**
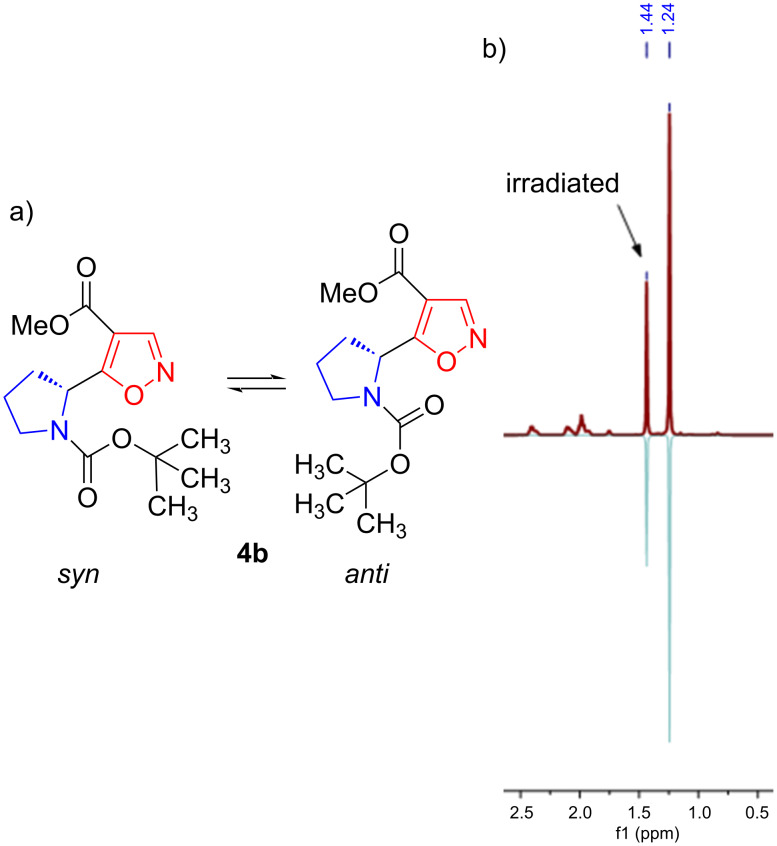
(a) Structure of **4b** with *syn*- and *anti*-conformers; (b) superimposed ^1^H NMR and 1D gradient NOE spectra with a selective irradiation of signal at δ 1.44 ppm.

The structure of the newly synthesized chiral 1,2-oxazole derivatives **4b**–**g** was described and confirmed via NMR spectroscopy data (Supporting Information File 1 in Figures S21–S38). In ^1^H NMR and ^13^C NMR spectra of compounds **4b** and **4c**, double sets of signals with different intensities were observed. It is possible that these signals are associated with the presence of equilibrating conformers arising from the rotation of the *tert*-butoxycarbonyl (Boc) moiety around a C–N single bond (Figure 4a). It is known that some *N*-Boc-substituted heterocyclic compounds, including oxazolidines, consist of NMR spectra showing two sets of signals due to the dynamic equilibrium between the two conformers formed by the rotation of the Boc moiety, and their *syn-* and *anti*-orientation is present in the molecule [45–46].

Ley et al. demonstrated that selective chemical exchange NMR experiments are very useful in the determination of equilibrating rotamers, including chiral *N*-Boc amino acid derivatives, from non-equilibrating diastereomers [47]. Chemical exchange NMR experiments such as saturation transfer have been widely used in such cases (e.g., 1D selective NOESY). In this case, the ^1^H NMR spectrum of **4b** revealed singlets of *tert*-butyl protons at δ 1.24 ppm (major rotamer) and δ 1.44 ppm (minor rotamer). When the signal at δ 1.44 ppm was irradiated, two negative signals of the same phase at δ 1.44 ppm and δ 1.24 ppm were observed (Figure 4b; Supporting Information File 1 in Figure S53a). Additionally, when the pyrrolidine ring proton 2-H (δ 5.61–5.65 ppm) was irradiated, two negative signals of the same phase at δ 5.51–5.56 ppm and δ 5.61–5.65 ppm appeared, implying chemical exchange and therefore the presence of rotamers (Supporting Information File 1 in Figure S53b).

The synthesis of methyl 5-(*N*-Boc-piperidin-4-yl)-1,2-oxazole-4-carboxylate (**4h**), a nonchiral amino acid-like building block, was obtained by the reaction of β-enamino ketoester **3h** with hydroxylamine hydrochloride (Scheme 1). The ^1^H NMR spectrum of compound **4h** showed a characteristic resonance for the Boc-group methyl protons, a singlet at δ 1.47 ppm, while the 1,2-oxazole methine proton appeared as a singlet at δ 8.46 ppm. The ^13^C NMR spectrum of **4h** contained the characteristic signals of the 1,2-oxazole ring skeleton carbons at δ 108.3 (C-4), 150.2 (C-3), and 179.5 (C-5) ppm. The ^15^N NMR spectrum of **4h** exhibited characteristic resonances of nitrogen atoms at δ −294.6 (piperidine) and δ −3.1 (1,2-oxazole) ppm, respectively.

**Scheme 4:**
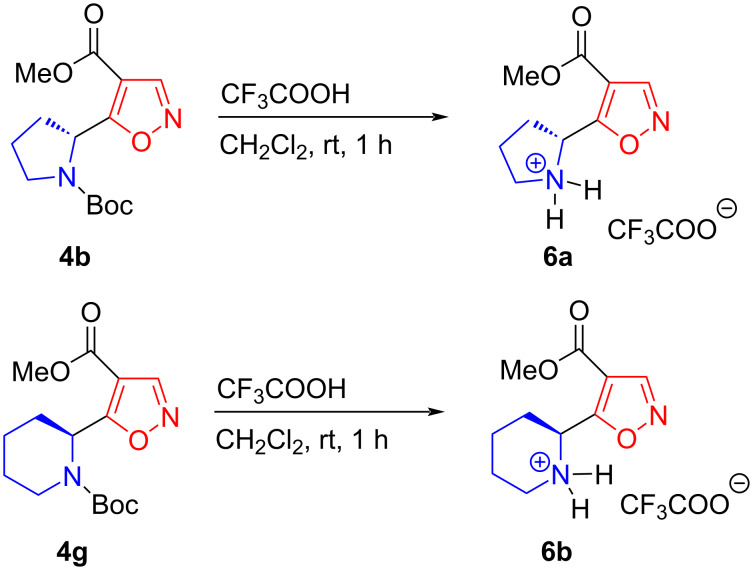
Synthesis of 2-[4-(methoxycarbonyl)-1,2-oxazol-5-yl]cycloaminyl-1-ium trifluoroacetates **6a**,**b**.

Next, we studied the reaction of compounds **4b** and **4g** with trifluoroacetic acid. Removal of the Boc protection in the presence of CF_3_COOH in dichloromethane yielded trifluoroacetates **6a** and **6b** as white solids (Scheme 4). A single crystal of **6b** was prepared via recrystallization from dichloromethane for X-ray diffraction analysis [48].

**Figure 5:**
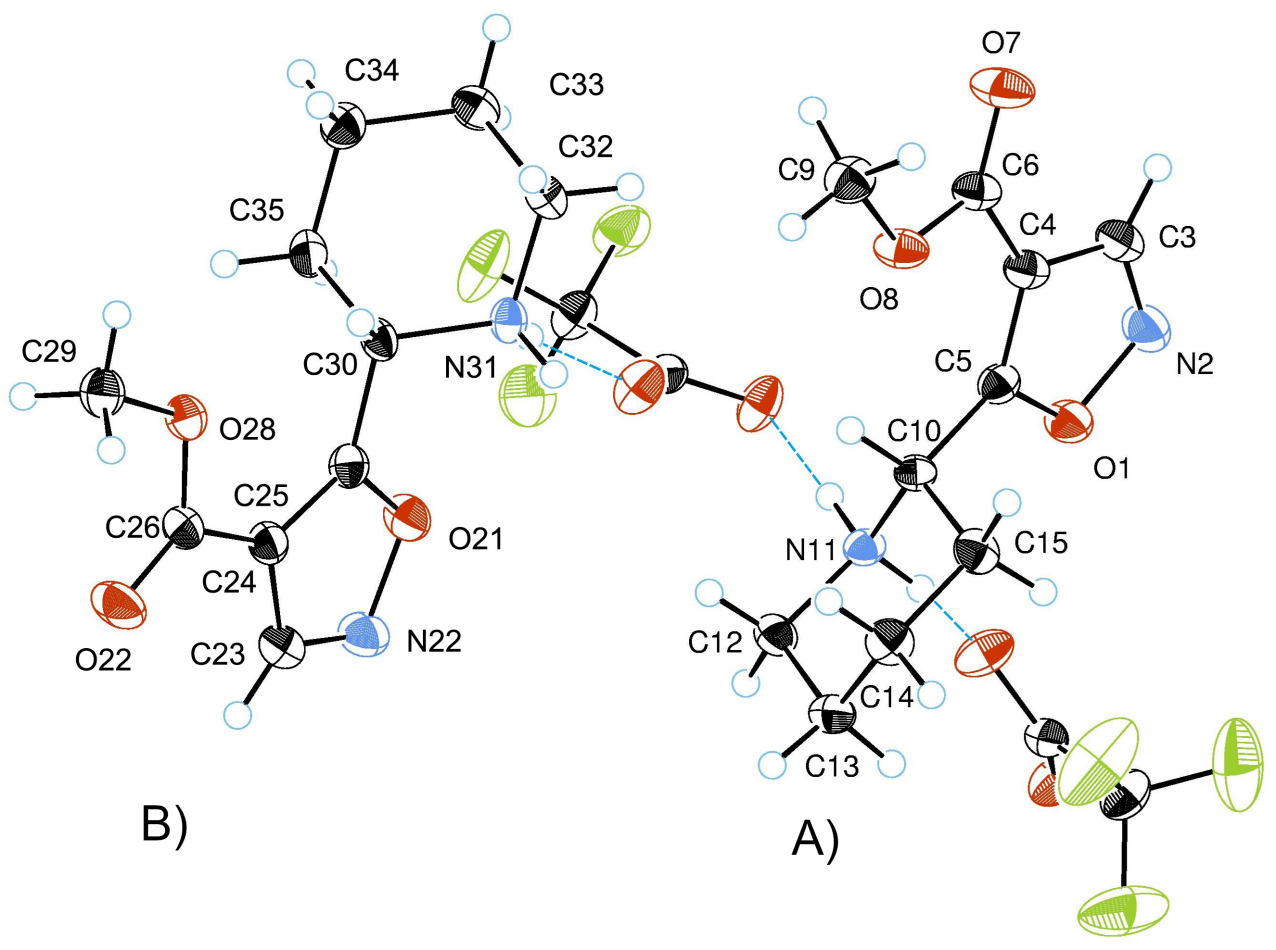
ORTEP diagram of the asymmetric unit consisting of two cations **6b**(**A**) and **6b**(**B**) and triflate anions.

The asymmetric unit of the crystal of **6b** consists of two (2*S*)-2-[4-(methoxycarbonyl)-1,2-oxazol-5-yl]piperidin-1-ium cations and two 2,2,2-trifluoroacetate anions (2C_10_H_15_N_2_O_3_^+^·2C_2_F_3_O_2_^−^) (Figure 5; Table 1, Supporting Information File 1 in Tables S1–S3). The substituted piperidinium moieties are in chair conformation. The 1,2-oxazole rings occupy equatorial positions at the 2nd atom of the piperidinium ring, and the dihedral angle H(10)–C(10)–C(5)–O(1) is 158°. The methoxycarbonyl group is in the same plane as the 1,2-oxazole ring.

**Table 1:** Bond lengths and angles of 1,2-oxazole fragments in an asymmetric unit of **6b**.

Molecule **6b**(**A**)	*d*, (Å)	Φ, deg	

O(1)–N(2)	1.420(2)	O(1)–N(2)–C(3)	105.00(16)
N(2)–C(3)	1.298(3)	N(2)–C(3)–C(4)	112.61(17)
C(3)–C(4)	1.424(3)	C(3)–C(4)–C(5)	104.01(16)
C(4)–C(5)	1.361(2)	C(4)–C(5)–O(1)	109.44(16)
C(5)–O(1)	1.351(2)	C(5)–O(1)–N(2)	108.95(14)

Molecule **6b**(**B**)	*d*, (Å)	Φ*,* deg	

O(21)–N(22)	1.419(2)	O(21)–N(22)–C(23)	104.71(16)
N(22)–C(23)	1.305(3)	N(22)–C(23)–C(24)	112.48(17)
C(23)–C(24)	1.425(3)	C(23)–C(24)–C(25)	103.89(16)
C(24)–C(25)	1.360(2)	C(24)–C(25)–O(21)	109.68(16)
C(25)–O(21)	1.345(2)	C(25)–O(21)–N(22)	109.23(14)

The bond lengths and angles of 1,2-oxazole rings are shown in Table 1 which correlates with the literature data [49]. In addition, there were some marginal differences between the bond lengths in the two 1,2-oxazole rings.

## Conclusion

A series of novel 1,2-oxazole-4-carboxylate derivatives possessing Boc-protected 4-, 5- and 6-membered saturated nitrogen heterocycles were synthesized as amino-acid-like building blocks. Construction of 5-cycloaminyl-1,2-oxazole compounds was based on the reaction of β-enamino ketoester precursors with hydroxylamine hydrochloride in moderate yields. When using the starting chiral saturated *N*-heterocyclic carboxylic acids, the target adducts were obtained with up to 97–100% ee. Discrimination between the regioisomeric compounds methyl 5-(*N*-Boc-azetidin*-*3-yl)-1,2-oxazole-4-carboxylate and methyl 3-(*N-*Boc-azetidin-3-yl)-1,2-oxazole-4-carboxylate was based on data from ^1^H, ^13^C and ^15^N NMR experiments.

In the NMR spectra of chiral 1,2-oxazoles, two sets of signals with different intensities were observed due to the existence of two Boc-group rotational conformers. The X-ray structure of (2*S*)-2-[4-(methoxycarbonyl)-1,2-oxazol-5-yl]piperidin-1-ium trifluoroacetate (**6b**) finally supported this structure analysis.

## Supporting Information

File 1 General information, synthesis procedures, and spectral data.
