# Supplementary material for: Regioselective synthesis of methyl 5-(N-Boc-cycloaminyl)-1,2-oxazole-4-carboxylates as new amino acid-like building blocks
Source: Beilstein J Org Chem. 2022 Jan 12;18:102–9. doi: 10.3762/bjoc.18.11 (PMC8767561; doi:10.3762/bjoc.18.11)
Supplement: File 1 — General information, synthesis procedures, and spectral data. [file Beilstein_J_Org_Chem-18-102-s001.pdf]

BEILSTEIN JOURNAL OF ORGANIC CHEMISTRY

Supporting Information

for

Regioselective synthesis of methyl 5-(*N*-Boc-cycloaminyl)-1,2-oxazole-4-carboxylates as new amino acid-like building blocks

Jolita Bruzgulienė, Greta Račkauskienė, Aurimas Bieliauskas, Vaida Milišiūnaitė, Miglė Dagilienė, Gita Matulevičiūtė, Vytas Martynaitis, Sonata Krikštolaitytė, Frank A. Sløk and Algirdas Šačkus

Beilstein J. Org. Chem. **2022,** *18,* 102–109. doi:10.3762/bjoc.18.11

General information, synthesis procedures, and spectral data

License and Terms: This is a supporting information file under the terms of the Creative Commons Attribution License (https://creativecommons.org/licenses/by/4.0). Please note that the reuse, redistribution and reproduction in particular requires that the author(s) and source are credited and that individual graphics may be subject to special legal provisions.
The license is subject to the *Beilstein Journal of Organic Chemistry* terms and conditions: (https://www.beilstein-journals.org/bjoc/terms)

Table of contents

1. General information	S2
2. General procedure for the synthesis of *β*-enamino keto esters **3a–h**	S3
3. General procedure for the synthesis of compounds **4a–h**	S7
4. Methyl 5-[1-(*tert*-butoxycarbonyl)azetidin-3-yl]-^{15}N-1,2-oxazole-4-carboxylate (**5**)	S13
5. Synthesis of compounds **6a**,**b**	S14
6. ^{1}H, ^{13}C and ^{15}N NMR spectra of new compounds **3a**–**h**, **4a**–**h**, **5**, **6a**,**b**	S15
7. Stacked chromatogram profile view of isolated enantiomers **4d–g**	S41
8. The ^{1}H NMR and 1D gradient NOE spectra of compound **4b**	S42
9. X-ray analysis of compound **6b**	S43
10. References	S46

1. General information

All reagents and solvents were purchased from commercial sources and were used without further purification. Analytical thin layer chromatography (TLC) was performed on aluminum foil backed plates (Merck Kieselgel 60 F254). Visualization of the compounds was effected by UV light (254 nm). Column chromatography was performed on silica gel SI 60 (43–60 mm, E. Merck). Melting points were determined on a Büchi M-565 melting point apparatus and were uncorrected. The IR spectra were recorded on a Bruker Vertex 70v FTIR spectrometer using neat samples. High-resolution ESI TOF mass spectra were measured on a Bruker maXis and Bruker MicrOTOF-Q III spectrometers. The ^{1}H, ^{13}C, and ^{15}N NMR spectra were recorded in $CDCl_3$ and DMSO-d_6 solutions at 25 °C on a Bruker Avance III 700 (700 MHz for ^{1}H, 176 MHz for ^{13}C, 71 MHz for ^{15}N) spectrometer equipped with a 5 mm TCI ^{1}H-^{13}C/^{15}N/D z-gradient cryoprobe, and a Bruker Avance III 400 (400 MHz for ^{1}H, 101 MHz for ^{13}C, 40 MHz for ^{15}N) spectrometer using a 5 mm directly detecting BBO probe. The chemical shifts (δ) expressed in ppm, were relative to tetramethylsilane (TMS). The ^{15}N NMR spectra were referenced to neat, external nitromethane (coaxial capillary). Rotation angles of the solutions of chiral compounds in chloroform were measured with polarimeter Unipol L, with a 10 cm optical length cell at 22 °C. Single crystals of $C_{12}H_{15}F_3N_2O_5$ were investigated on a Rigaku, XtaLAB Synergy, Dualflex, HyPix diffractometer. The crystal was kept at 150.0(1) K during data collection. Using Olex2 [1], the structure was solved with the ShelXT [2] structure solution program using Intrinsic Phasing and refined with the olex2.refine [3] refinement package using Gauss-Newton minimization.

Analytical chromatographic chiral separations were carried out on a Chiral Art Amylose-SA and Chiral Art Cellulose-SB columns (100 mm × 4.6 mm, 3 µm) with a mobile phase consisting of water + 0.1% formic acid/acetonitrile, at a flow rate of 1 mL/min and maintaining the column

temperature at 36 °C. Compounds **4b**,**c** were separated on a Cellulose-SB column, isocratic mode water + 0.1% formic acid/acetonitrile 70:30. Compounds **4d**,**e** were separated on an Amylose-SA column gradient conditions from water + 0.1% formic acid/acetonitrile 70:30 to 50:50 in 10 minutes. Compounds **4f**,**g** were separated on an Amylose-SA column isocratic mode water + 0.1% formic acid/acetonitrile 70:30. Samples were prepared in methanol. The injection volume was 10 µL, sampler temperature was set at 15 °C, and the detection wavelength was set at 245 nm. Intermediate compounds **2a** [4], **2b** [5], **2c**, **2d**, **2e**, **2f**, **2g** [6] and **2h** [7,8] were obtained according to literature data. The synthesis of compound **3h** was obtained according to the patent [7].

2. General procedure for the synthesis of β-enamino keto esters 3a–h

The β-keto ester **2a**–**h** (21.6 mmol) was dissolved in 1,4-dioxane (40 mL) and *N*,*N*-dimethylformamide dimethylacetal (3.59 mL, 27 mmol) was added under argon atmosphere and the resulting mixture was stirred at 80°C for 4 h. After removal of the solvent in vacuo, the residue was purified by flash chromatography on silica gel (Hex/EtOAc 2:1 v/v) to give compound (**3a-h**) as a yellowish oil.

2.1. *tert*-Butyl 3-[3-(dimethylamino)-2-(methoxycarbonyl)prop-2-enoyl]azetidine-1-carboxylate (3a)

carboxylate (3a) yellowish oil (5.06 g, 75%). IR, (ν_{max}, cm^{-1}): 2974 (CH-aliph), 1688 (C=O), 1639 (C=O), 1576, 1365, 1117, 773. ^{1}H NMR (700 MHz, $CDCl_3$): δ 1.43 (s, 9H, 3 × CH_3), 2.87 (s, 3H, -$N(CH_3)_2$), 3.30 (s, 3H, -$N(CH_3)_2$), 3.73 (s, 3H, O-CH_3), 3.83–3.90 (m, 1H, Az 3-H), 4.00–4.08 (m, 4H, Az 2,4-H), 7.80 (s, 1H, =C*H*-$N(CH_3)_2$). ^{13}C NMR (176 MHz, $CDCl_3$): δ 28.4 (3×CH_3), 38.2

(Az C-3), 42.4 (-N(CH_3)$_2$), 47.9 (-N(CH_3)$_2$), 51.1 (O-CH_3), 51.4 and 52.4 (Az C-2,4), 79.2 [C(CH_3)$_3$], 99.8 (C=CH-N(CH_3)$_2$), 156.3 (*N*-Boc C=O), 159.0 (C=CH-N(CH_3)$_2$), 167.7 (COOMe C=O), 195.4 (C=O). ^{15}N NMR (71 MHz, $CDCl_3$): δ -309.6 (*N*-Boc), -269.7 (C=CH-N(CH_3)$_2$). HRMS (ESI), m/z: calcd. for $C_{15}H_{24}N_2O_5Na^+$ 335.1577 [M+Na]$^+$; found 335.1576.

2.2. *tert*-Butyl (2*R*)-2-[3-(dimethylamino)-2-(methoxycarbonyl)prop-2-enoyl]pyrrolidine-1-carboxylate (3b) yellowish oil (6.70 g, 95%). IR, (ν_{max}, cm^{-1}): 2976 (CH-aliph), 1674 (C=O), 1644 (C=O), 1584, 1404, 1094, 768. ^{1}H NMR (700 MHz, $CDCl_3$): (two rotamers are seen in the spectra ratio~1:0.8) δ 1.37 (s, 7.2H, 3 × CH_3 *of minor rotamer*), 1.42 (s, 9H, 3 × CH_3 *of major rotamer*), 1.76–2.00 (m, 5.4H *of both rotamers*), 2.17–2.32 (m, 1.8H *of both rotamers*), 2.88 (s, 5.4H, -N(CH_3)$_2$ *of both rotamers*), 3.25 (s, 5.4H, -N(CH_3)$_2$ *of both rotamers*), 3.39–3.49 (m, 1.8H *of both rotamers*), 3.53–3.59 (m, 1.8H *of both rotamers*), 3.71 (s, 3H, O-CH_3 *of major rotamer*), 3.75 (s, 2.4H, O-CH_3 *of minor rotamer*), 4.82–5.10 (m, 1.8H *of both rotamers*), 7.78 (s, 0.8H, =C*H*-N(CH_3)$_2$ *of minor rotamer*), 7.80 (s, 1H, =C*H*-N(CH_3)$_2$ *of major rotamer*). ^{13}C NMR (176 MHz, $CDCl_3$): δ 23.2, 24.1, 28.38, 28.42, 30.9, 31.3, 41.9, 42.5, 46.8, 47.4, 47.9, 50.8, 51.0, 62.9, 63.2, 78.9, 79.0, 99.6, 99.8, 154.2, 154.5, 158.5, 159.5, 168.1, 196.2, 197.4. HRMS (ESI), m/z: calcd. for $C_{16}H_{26}N_2O_5Na^+$ 349.1734 [M+Na]$^+$; found 349.1736.

2.3. *tert*-Butyl (2*S*)-2-[3-(dimethylamino)-2-(methoxycarbonyl)prop-2-enoyl]pyrrolidine-1-carboxylate (3c) yellowish oil (6.42 g, 91%). IR, (ν_{max}, cm^{-1}): 2976 (CH-aliph), 1673 (C=O), 1644 (C=O), 1583, 1403, 1093, 767. ^{1}H NMR (700 MHz, $CDCl_3$): (two rotamers are seen in the spectra ratio~1:0.8) δ 1.37 (s, 7.2H, 3 × CH_3 *of minor rotamer*), 1.42 (s, 9H, 3 × CH_3 *of major rotamer*), 1.75–2.01 (m, 5.4H *of both rotamers*), 2.17–2.33 (m, 1.8H *of both rotamers*), 2.89 (s, 5.4H, -

$N(CH_3)_2$ *of both rotamers*), 3.25 (s, 5.4H, -$N(CH_3)_2$ *of both rotamers*), 3.39–3.49 (m, 1.8H *of both rotamers*), 3.54–3.59 (m, 1.8H *of both rotamers*), 3.70 (s, 3H, O-CH_3 *of major rotamer*), 3.75 (s, 2.4H, O-CH_3 *of minor rotamer*), 4.92–5.02 (m, 1.8H *of both rotamers*), 7.78 (s, 0.8H, =C*H*-$N(CH_3)_2$ *of minor rotamer*), 7.80 (s, 1H, =C*H*-$N(CH_3)_2$ *of major rotamer*). ^{13}C NMR (176 MHz, $CDCl_3$): δ 23.2, 24.2, 28.41, 28.44, 30.9, 31.3, 41.9, 42.5, 46.8, 47.4, 47.9, 50.8, 51.0, 63.0, 63.3, 78.9, 79.0, 99.6, 99.9, 154.2, 154.5, 158.5, 159.5, 168.1, 196.3, 197.6. HRMS (ESI), m/z: calcd. for $C_{16}H_{26}N_2O_5Na^+$ 349.1734 $[M+Na]^+$; found 349.1739.

2.4. *tert*-Butyl (3*S*)-3-[3-(dimethylamino)-2-(methoxycarbonyl)prop-2-enoyl]pyrrolidine-1-carboxylate (3d) yellowish oil (5.99 g, 85%). IR, (ν_{max}, cm^{-1}): 2976 (CH-aliph), 1684 (C=O), 1643 (C=O), 1580, 1402, 1096, 771. ^{1}H NMR (700 MHz, $CDCl_3$): (two rotamers are seen in the spectra ratio~1:0.8) δ 1.45 (s, 9H, 3 × CH_3 *of both rotamers*), 1.99–2.19 (m, 2H *of both rotamers*), 2.79 (s, 3H, -$N(CH_3)_2$ *of both rotamers*), 3.27 (s, 3H, -$N(CH_3)_2$ *of both rotamers*), 3.28–3.36 (m, 1H *of both rotamers*), 3.41–3.52 (m, 2H *of both rotamers*), 3.52–3.62 (m, 1H *of both rotamers*), 3.75 (s, 1.3H, O-CH_3 *of minor rotamer*), 3.76 (s, 1.7H, O-CH_3 *of major rotamer*), 3.80 (m, 1H *of both rotamers*), 7.70-7.74 (m, 1H, =C*H*-$N(CH_3)_2$ *of both rotamers*. ^{13}C NMR (176 MHz, $CDCl_3$): δ 28.5, 29.1, 29.3, 41.9, 45.5, 45.9, 47.8, 49.1, 49.2, 51.2, 79.0, 79.02, 101.2, 154.4, 154.5, 157.8, 168.32, 168.35, 197.3. HRMS (ESI), m/z: calcd. for $C_{16}H_{26}N_2O_5Na^+$ 349.1734 $[M+Na]^+$; found 349.1738.

2.5. *tert*-Butyl (3*R*)-3-[3-(dimethylamino)-2-(methoxycarbonyl)prop-2-enoyl]pyrrolidine-1-carboxylate (3e) yellowish oil (5.78 g, 82%). IR, (ν_{max}, cm^{-1}): 2975 (CH-aliph), 1685 (C=O), 1641 (C=O), 1577, 1403, 1112, 772. ^{1}H NMR (700 MHz, $CDCl_3$): (two rotamers are seen in the spectra

ratio~1:0.8) δ 1.45 (s, 9H, 3 × CH_3 *of both rotamers*), 2.01–2.17 (m, 2H *of both rotamers*), 2.79 (s, 3H, -$N(CH_3)_2$ *of both rotamers*), 3.25 (s, 3H, -$N(CH_3)_2$ *of both rotamers*), 3.28–3.37 (m, 1H *of both rotamers*), 3.41–3.51 (m, 2H *of both rotamers*), 3.52–3.63 (m, 1H *of both rotamers*), 3.74 (s, 1.3H, O-CH_3 *of minor rotamer*), 3.76 (s, 1.7H, O-CH_3 *of major rotamer*), 3.80 (m, 1H *of both rotamers*), 7.70-7.75 (m, 1H, =C*H*-$N(CH_3)_2$ *of both rotamers*. ^{13}C NMR (176 MHz, $CDCl_3$): δ 28.5, 29.15, 29.33, 41.9, 45.55, 45.88, 47.8, 49.09, 49.23, 51.2, 78.95, 79.02, 101.2, 154.45, 154.52, 157.9, 168.4, 197.3. HRMS (ESI), m/z: calcd. for $C_{16}H_{26}N_2O_5Na^+$ 349.1734 $[M+Na]^+$; found 349.1735.

2.6. *tert*-Butyl (2*R*)-2-[3-(dimethylamino)-2-(methoxycarbonyl)prop-2-enoyl]piperidine-1-carboxylate (3f) yellowish oil (5.66 g, 77%). IR, (ν_{max}, cm^{-1}): 2975 (CH-aliph), 1685 (C=O), 1644 (C=O), 1579, 1421, 1159, 729. ^1H NMR (700 MHz, $CDCl_3$): δ 1.15–1.35 (m, 1H), 1.37–1.51 (m, 9H), 1.52–1.79 (m, 3H), 1.96–2.11 (m, 1H), 2.86 (s, 3H), 2.99–3.11 (m, 2H), 3.22 (s, 3H), 3.74 (s, 3H), 3.82–4.06 (m, 1H), 5.34–5.69 (m, 1H), 7.66–7.78 (m, 1H). ^{13}C NMR (176 MHz, $CDCl_3$): δ 20.6, 24.9, 25.2, 26.5, 27.0, 28.28, 28.41, 41.4, 42.7, 47.7, 51.13, 51.40, 57.8, 58.5, 79.2, 99.8, 100.9, 156.1, 158.2, 168.1, 196.6, 196.8. HRMS (ESI), m/z: calcd. for $C_{17}H_{28}N_2O_5Na^+$ 363.1890 $[M+Na]^+$; found 363.1892.

2.7. *tert*-Butyl (2*S*)-2-[3-(dimethylamino)-2-(methoxycarbonyl)prop-2-enoyl]piperidine-1-carboxylate (3g) yellowish oil (5.59 g, 76%). IR, (ν_{max}, cm^{-1}): 2973 (CH-aliph), 1686 (C=O), 1644 (C=O), 1579, 1421, 1158, 730. ^1H NMR (700 MHz, $CDCl_3$): δ 1.17–1.29 (m, 1H), 1.32–1.53 (m, 9H), 1.53–1.80 (m, 3H), 1.95–2.11 (m, 1H), 2.86 (s, 3H), 2.98–3.11 (m, 2H), 3.22 (s, 3H), 3.74 (s, 3H), 3.82–4.07 (m, 1H), 5.31–5.70 (m, 1H), 7.64–7.80 (m, 1H). ^{13}C NMR (176 MHz, $CDCl_3$):

δ 18.7, 22.99, 23.27, 24.7, 25.1, 26.40, 26.53, 39.5, 40.8, 45.9, 49.25, 49.52, 55.9, 56.6, 77.3, 98.0, 98.9, 154.2, 156.3, 166.2, 194.69, 194.97. HRMS (ESI), m/z: calcd. for $C_{17}H_{28}N_2O_5Na^+$ 363.1890 $[M+Na]^+$; found 363.1893.

2.8. *tert*-Butyl 4-[3-(dimethylamino)-2-(methoxycarbonyl)prop-2-enoyl]piperidine-1-carboxylate (3h) yellowish oil (5.22 g, 71%). IR, (ν_{max}, cm^{-1}): 2974 (CH-aliph), 1683 (C=O), 1642 (C=O), 1579, 1365, 1122, 729. ^1H NMR (700 MHz, $CDCl_3$): δ 1.45 (s, 9H), 1.50–1.59 (m, 2H), 1.70–1.83 (m, 2H), 2.61–2.94 (m, 5H), 3.05–3.40 (m, 4H), 3.74 (s, 3H), 4.03–4.24 (m, 2H), 7.69 (s, 1H). ^{13}C NMR (176 MHz, $CDCl_3$): δ 28.47, 28.84, 41.7, 43.3, 44.0, 46.3, 47.7, 51.1, 79.3, 100.8, 154.8, 157.5, 168.6, 200.5. HRMS (ESI), m/z: calcd. for $C_{17}H_{28}N_2O_5Na^+$ 363.1890 $[M+Na]^+$; found 363.1892.

3. General procedure for the synthesis of 1,2-oxazole-4-carboxylates 4a–h

To a solution of appropriate β-enamino keto ester **3a**–**h** (5 mmol) in ethanol (**3a** and **3h**, 50 mL) or in methanol (**3b**–**g**, 33 mL), hydroxylamine hydrochloride (0.52 g, 7.5 mmol) was added and the reaction mixture was stirred and refluxed for 4 h (**3a** and **3h**) or was stirred at rt for 20 h (**3b**–**g**). The solvent was removed under reduced pressure and the residue was purified by column chromatography using an appropriate eluent.

3.1. Methyl 5-[1-(*tert*-butoxycarbonyl)azetidin-3-yl]-1,2-oxazole-4-carboxylate (4a)

Purified by column chromatography on silica gel with (Hex/EtOAc 4:1 v/v) to afford the title compound **4a** as white crystalline solid (0.92 g, 65%), mp 84–85 ºC (from ethyl acetate). IR,

(ν_{max}, cm^{-1}): 3014 (CH-arom), 2967 (CH-aliph), 1723 (C=O), 1687 (C=O), 1407, 1137, 1091, 771. ^{1}H NMR (700 MHz, $CDCl_3$): δ 1.46 (s, 9H, 3 × CH_3), 3.87 (s, 3H, O-CH_3), 4.24 (dd, *J* = 8.7, 6.5 Hz, 2H, Az 2,4-H), 4.32 (t, *J* = 8.8 Hz, 2H, Az 2,4-H), 4.48 (tt, *J* = 8.9, 6.5 Hz, 1H, Az 3-H), 8.52 (s, 1H, Isox 3-H). ^{13}C NMR (176 MHz, $CDCl_3$): δ 26.0 (Az C-3), 28.4 (3×*C*H_3), 52.1 (O-*C*H_3), 52.9 (Az C-2,4), 80.1 [*C*$(CH_3)_3$], 109.8 (Isox C-4), 150.5 (Isox C-3), 156.1 (*N*-Boc C=O), 161.5 (C=O), 175.3 (Isox C-5). ^{15}N NMR (71 MHz, $CDCl_3$): δ -311.8 (*N*-Boc), -1.2 (*N*-Isox). HRMS (ESI), m/z: calcd. for $C_{13}H_{18}N_2O_5Na^+$ 305.1108 $[M+Na]^+$; found 305.1108.

3.2. Methyl 5-[(2*R*)-1-(*tert*-butoxycarbonyl)pyrrolidin-2-yl]-1,2-oxazole-4-carboxylate (4b)

Purified by column chromatography on silica gel with (Hex/EtOAc 8:1 v/v) to afford the title compound **4b** as colorless oil (0.92 g, 62%), $[\alpha]_D^{23}$ -7.8 (c 2.55 $CHCl_3$). IR, (ν_{max}, cm^{-1}): 3011 (CH-arom), 2976 (C-aliph), 1723 (C=O), 1695 (C=O), 1389, 1242, 1061, 780. ^{1}H NMR (700 MHz, $CDCl_3$): (two rotamers are seen in the spectra ratio~1:0.6) δ 1.25 (s, 9H, 3 × CH_3 *of major rotamer*), 1.44 (s, 5.4H, 3 × CH_3 *of minor rotamer*), 1.90–2.03 (m, 3.2H, Pyr 4-H_a, Pyr 3-H_a *of both rotamers*), 2.04–2.15 (m, 1.6H, Pyr 4-H_b, *of both rotamers*), 2.34–2.45 (m, 1.6H, Pyr 3-H_b *of both rotamers*), 3.48–3.62 (m, 1.6H, Pyr 5-H_b *of both rotamers*), 3.63–3.71 (m, 1.6H, Pyr 5-H_a *of both rotamers*), 3.85 (s, 1.8H, O-CH_3 *of minor rotamer*), 3.87 (s, 3H, O-CH_3 *of major rotamer*), 5.54 (dd, *J* = 8.0, 4.4 Hz, 1H, Pyr 2-H *of major rotamer*), 5.63 (dd, *J* = 8.2, 3.1 Hz, 0.6H, Pyr 2-H *of minor rotamer*), 8.47 (s, 0.6H, Isox 3-H *of minor rotamer*), 8.49 (s, 1H, Isox 3-H *of major rotamer*). ^{13}C NMR (176 MHz, $CDCl_3$): δ 23.9 (Pyr C-4 *of major rotamer*), 24.4 (Pyr C-4 *of minor rotamer*), 28.0 (3 × CH_3 *of major rotamer*), 28.4 (3 × CH_3 *of minor rotamer*), 32.1 (Pyr C-3 *of minor rotamer*), 32.7 (Pyr C-3 *of major rotamer*), 46.6 (Pyr C-5 *of major rotamer*), 46.9 (Pyr C-5 *of minor rotamer*), 51.9 (O-CH_3 *of minor rotamer*), 52.0 (O-CH_3 *of major rotamer*),

53.4 (Pyr C-2 *of major rotamer*), 53.7 (Pyr C-2 *of minor rotamer*), 80.01 [*C*(CH_3)$_3$ *of major rotamer*], 80.04 [*C*(CH_3)$_3$ *of minor rotamer*], 108.41 (Isox C-4 *of major rotamer*), 108.45 (Isox C-4 *of minor rotamer*), 150.0 (Isox C-3 *of major rotamer*), 150.3 (Isox C-3 *of minor rotamer*), 153.5 (*N*-Boc C=O *of major rotamer*), 154.1 (*N*-Boc C=O *of minor rotamer*), 161.6 (C=O *of both rotamers*), 177.8 (Isox C-5 *of minor rotamer*), 178.1 (Isox C-5 *of major rotamer*). ^{15}N NMR (71 MHz, $CDCl_3$): δ -283.8 (*N*-Boc *of both rotamers*), -3.3 (*N*-Isox *of minor rotamer*), -3.2 (*N*-Isox *of major rotamer*). HRMS (ESI), m/z: calcd. for $C_{14}H_{20}N_2O_5Na^+$ 319.1264 [M+Na]$^+$; found 319.1264.

3.3. Methyl 5-[(2*S*)-1-(*tert*-butoxycarbonyl)pyrrolidin-2-yl]-1,2-oxazole-4-carboxylate (4c)

Purified by column chromatography on silica gel with (Hex/Acetone 5:1 v/v) to afford the title compound **4c** as colorless oil (0.86 g, 58%), α]$_D^{23}$ 7.9 (c 2.78 $CHCl_3$). IR, (ν_{max}, cm^{-1}): 3077 (CH-arom), 2976 (CH-aliph), 1722 (C=O), 1694 (C=O), 1389, 1241, 1080, 780. ^{1}H NMR (400 MHz, $CDCl_3$): (two rotamers are seen in the spectra ratio~1:0.6) δ 1.23 (s, 9H, 3 × CH_3 *of major rotamer*), 1.42 (s, 9H, 3 × CH_3 *of minor rotamer*), 1.92–2.02 (m, 3.2H, Pyr 4-H_a, Pyr 3-H_a *of both rotamers*), 2.04–2.13 (m, 1.6H, Pyr 4-H_b, *of both rotamers*), 2.33–2.44 (m, 1.6H, 3-H_b *of both rotamers*), 3.46–3.58 (m, 1.6H, Pyr 5-H_b *of both rotamers*), 3.63–3.70 (m, 1.6H, Pyr 5-H_a *of both rotamers*), 3.84 (s, 1.8H, O-CH_3 *of minor rotamer*), 3.86 (s, 3H, O-CH_3 *of major rotamer*), 5.50–5.54 (m, 1H, Pyr 2-H *of major rotamer*), 5.60–5.63 (m, 0.6H, Pyr 2-H *of minor rotamer*), 8.46 (s, 0.6H, Isox 3-H *of minor rotamer*), 8.47 (s, 1H, Isox 3-H *of major rotamer*). ^{13}C NMR (100 MHz, $CDCl_3$): δ 24.0 (Pyr C-4 *of major rotamer*), 24.5 (Pyr C-4 *of minor rotamer*), 28.1 (3 × CH_3 *of major rotamer*), 28.5 (3 × CH_3 *of minor rotamer*), 32.2 (Pyr C-3 *of minor rotamer*), 32.8 (Pyr C-3 *of major rotamer*), 46.8 (Pyr C-5 *of major rotamer*), 47.0 (Pyr C-5 *of minor rotamer*), 52.1 (O-CH_3 *of both rotamers*), 53.5 (Pyr C-2 *of major rotamer*), 53.8 (Pyr C-2 *of minor rotamer*), 80.1

[$C(CH_3)_3$], 108.5 (Isox C-4 *of both rotamers*), 150.1 (Isox C-3 *of major rotamer*), 150.4 (Isox C-3 *of minor rotamer*), 153.6 (*N*-Boc C=O *of major rotamer*), 154.2 (*N*-Boc C=O *of minor rotamer*), 161.7 (C=O *of both rotamers*), 177.9 (Isox C-5 *of minor rotamer*), 178.2 (Isox C-5 *of major rotamer*). ^{15}N NMR (71 MHz, $CDCl_3$): δ -284.0 (*N*-Boc *of both rotamers*), -3.3 (*N*-Isox *of minor rotamer*), -3.2 (*N*-Isox *of major rotamer*). HRMS (ESI), m/z: calcd. for $C_{14}H_{20}N_2O_5Na^+$ 319.1264 $[M+Na]^+$; found 319.1264.

3.4. Methyl 5-[(3*S*)-1-(*tert*-butoxycarbonyl)pyrrolidin-3-yl]-1,2-oxazole-4-carboxylate (4d)

Purified by column chromatography on silica gel with (Hex/EtOAc 7:1 v/v) to afford the title compound **4d** as colorless oil (0.79 g, 53%), $[\alpha]_D^{23}$ -20.6 (c 3.11 $CHCl_3$). IR, (ν_{max}, cm^{-1}): 3014 (CH-arom), 2974 (CH-aliph), 1720 (C=O), 1686 (C=O), 1391, 1237, 1120, 770. ^1H NMR (400 MHz, $CDCl_3$): δ 1.43 (s, 9H, 3 × CH_3), 2.21–2.29 (m, 2H, Pyr 4-H_a, Pyr 4-H_b), 3.41–3.52 (m, 2H, Pyr 5-H_a, Pyr 2-H_a), 3.59–3.65 (m, 1H, Pyr 5-H_b), 3.71–3.77 (m, 1H, Pyr 2-H_b), 3.84 (s, 3H, O-CH_3), 4.10–4.24 (m, 1H, Pyr 3-H), 8.46 (s, 1H, Isox 3-H). ^{13}C NMR (100 MHz, $CDCl_3$): δ 28.5 (3 × CH_3), 29.6 (Pyr C-4 *of major rotamer*), 30.2 (Pyr C-4 *of minor rotamer*), 35.8 (Pyr C-3 *of minor rotamer*), 36.6 (Pyr C-3 *of major rotamer*), 45.4 (Pyr C-5 *of major rotamer*), 45.6 (Pyr C-5 *of minor rotamer*), 49.2 (Pyr C-2 *of both rotamers*), 52.1 (O-CH_3), 79.7 [$C(CH_3)_3$], 109.5 (Isox C-4), 150.3 (Isox C-3), 154.3 (*N*-Boc C=O), 161.7 (C=O), 176.2 (Isox C-5 *of major rotamer*), 176.5 (Isox C-5 *of minor rotamer*). ^{15}N NMR (71 MHz, $CDCl_3$): δ -292.2 (*N*-Boc of both rotamers), -1.9 (*N-Isox of both rotamers*). HRMS (ESI), m/z: calcd. for $C_{14}H_{20}N_2O_5Na^+$ 319.1264 $[M+Na]^+$; found 319.1265.

3.5. Methyl 5-[(3*R*)-1-(*tert*-butoxycarbonyl)pyrrolidin-3-yl]-1,2-oxazole-4-carboxylate (4e)

Purified by column chromatography on silica gel with (Hex/EtOAc 6:1 v/v) to afford the title compound **4e** as white crystalline solid (0.68 g, 46%), mp 96–97 ºC (from ethyl acetate), $[\alpha]_D^{23}$ 19.1 (c 3.3 $CHCl_3$). IR, (ν_{max}, cm^{-1}): 3014 (CH-arom), 2974 (CH-aliph), 1719 (C=O), 1685 (C=O), 1404, 1237, 1120, 770. ^1H NMR (400 MHz, $CDCl_3$): δ 1.46 (s, 9H, 3 × CH_3), 2.24–2.31 (m, 2H, Pyr 4-H_a, Pyr 4-H_b), 3.46–3.70 (m, 3H, Pyr 5-H_a, Pyr 2-H_a, Pyr 5-H_b), 3.75–3.82 (m, 1H, Pyr 2-H_b), 3.87 (s, 3H, O-CH_3), 4.19–4.25 (m, 1H, Pyr 3-H), 8.49 (s, 1H, Isox 3-H). ^{13}C NMR (100 MHz, $CDCl_3$): δ 28.6 (3 × CH_3), 29.7 (Pyr C-4 *of major rotamer*), 30.3 (Pyr C-4 *of minor rotamer*), 35.8 (Pyr C-3 *of minor rotamer*), 36.7 (Pyr C-3 *of major rotamer*), 45.5 (Pyr C-5 *of major rotamer*), 45.7 (Pyr C-5 *of minor rotamer*), 49.3 (Pyr C-2 *of both rotamers*), 52.1 (O-CH_3), 79.8 [*C*(CH_3)$_3$], 109.6 (Isox C-4), 150.4 (Isox C-3), 154.3 (*N*-Boc C=O), 161.8 (C=O), 176.3 (Isox C-5 *of major rotamer*), 176.6 (Isox C-5 *of minor rotamer*). ^{15}N NMR (71 MHz, $CDCl_3$): δ -292.1 (*N*-Boc of both rotamers), -1.9 (*N-Isox of both rotamers*). HRMS (ESI), m/z: calcd. for $C_{14}H_{20}N_2O_5Na^+$ 319.1264 $[M+Na]^+$; found 319.1265.

3.6. *tert*-Butyl (2*R*)-2-[4-(methoxycarbonyl)-1,2-oxazol-5-yl]piperidine-1-carboxylate (4f)

Purified by column chromatography on silica gel with (Hex/EtOAc 10:1 v/v) to afford the title compound **4f** as colorless oil (0.54 g, 35%), $[\alpha]_D^{23}$ -11.0 (c 2.02 $CHCl_3$). IR, (ν_{max}, cm^{-1}): 3004 (CH-arom), 2971 (CH-alph), 1719 (C=O), 1682 (C=O), 1393, 1224, 1157, 782. ^1H NMR (400 MHz, $CDCl_3$): δ 1.37 (s, 9H, 3 × CH_3), 1.46–1.56 (m, 2H, Pip 4-H), 1.66–1.69 (m, 1H, Pip 5-H_a), 1.75–1.83 (m, 1H, Pip 5-H_b), 1.91–2.04 (m, 2H, Pip 3-H), 3.35–3.38 (m, 1H, Pip 6-H_a), 3.85 (s, 3H, O-CH_3), 4.09–4.11 (m, 1H, Pip 6-H_b), 6.01 (s, 1H, Pip 2-H), 8.49 (s, 1H, Isox 3-H). ^{13}C NMR (100 MHz, $CDCl_3$): δ 19.7 (Pip C-4), 24.8 (Pip C-5), 28.4 (3 × CH_3), 29.1 (Pip C-3), 42.1 (br.s,

Pip C-6), 48.6 (Pip C-2), 52.1 (O-CH_3), 80.5 [$C(CH_3)_3$], 108.5 (Isox C-4), 150.2 (Isox C-3), 155.3 (*N*-Boc C=O), 161.5 (C=O), 178.6 (Isox C-5). ^{15}N NMR (41 MHz, $CDCl_3$): δ -292.3 (*N*-Boc), -1.9 (*N*-Isox). HRMS (ESI), m/z: calcd. for $C_{15}H_{22}N_2O_5Na^+$ 333.1421 $[M+Na]^+$; found 333.1421.

3.7. *tert*-Butyl (2*S*)-2-[4-(methoxycarbonyl)-1,2-oxazol-5-yl]piperidine-1-carboxylate (4g)

Purified by column chromatography on silica gel with (Hex/Acetone 5:1 v/v) to afford the title compound **4g** as colorless oil (0.57 g, 37%), $[\alpha]_D^{23}$ 11.2 (c 2.05 $CHCl_3$). IR, (ν_{max}, cm^{-1}): 3000 (CH-arom), 2975 (CH-aliph), 1732 (C=O), 1692 (C=O), 1394, 1234, 1153, 781. ^1H NMR (400 MHz, $CDCl_3$): δ 1.36 (s, 9H, 3 × CH_3), 1.44–1.56 (m, 2H, Pip 4,5-H_a), 1.65–1.67 (m, 1H, Pip 4-H_b), 1.75–1.83 (m, 1H, Pip 5-H_b), 1.90–2.02 (m, 2H, Pip 3-H), 3.34–3.37 (m, 1H, Pip 6-H_a), 3.85 (s, 3H, O-CH_3), 4.08–4.10 (m, 1H, Pip 6-H_b), 6.00 (br. s, 1H, Pip 2-H), 8.49 (s, 1H, Isox 3-H). ^{13}C NMR (100 MHz, $CDCl_3$): δ 19.7 (Pip C-4), 24.8 (Pip C-5), 28.4 (3 × CH_3), 29.1 (Pip C-3), 42.0 (br s, Pip C-6), 48.5 (Pip C-2), 52.1 (O-CH_3), 80.4 [$C(CH_3)_3$], 108.5 (Isox C-4), 150.2 (Isox C-3), 155.3 (*N*-Boc C=O), 161.5 (C=O), 178.6 (Isox C-5). ^{15}N NMR (71 MHz, $CDCl_3$): δ -292.7 (*N*-Boc), -2.2 (*N*-Isox). HRMS (ESI), m/z: calcd. for $C_{15}H_{22}N_2O_5Na^+$ 333.1421 $[M+Na]^+$; found 333.1421.

3.8. *tert*-Butyl 4-[4-(methoxycarbonyl)-1,2-oxazol-5-yl]piperidine-1-carboxylate (4h)

Purified by column chromatography on silica gel with (eluent: Hex/ EtOAc 4:1) to afford the title compound **4h** as white crystals (0.99 g, 64%), mp 91–92°C (from ethyl acetate). IR, (ν_{max}, cm^{-1}): 3015 (CH-arom), 2997 (C-aliph), 1728 (C=O), 1677 (C=O), 1432, 1225, 1060, 776. ^1H NMR (400 MHz, $CDCl_3$): δ 1.47 (s, 9H, 3 × CH_3), 1.79–1.89 (m, 4H, Pip 3,5-H_a, Pip 3,5-H_b), 2.76–2.95 (m, 2H, Pip 2-H_b, 6-H_b), 3.61–3.69 (m, 1H, Pip 4-H), 3.85 (s, 3H, O-CH_3), 4.13–4.30 (m, 2H, Pip

2-H_a, 6-H_a), 8.46 (s, 1H, Isox 3-H). ^{13}C NMR (100 MHz, $CDCl_3$): δ 28.6 (3 × CH_3), 29.3 (Pip C-3, C-5), 34.9 (Pip C-4), 43.6 (br. s, Pip C-2, C-6), 52.0 (O-CH_3), 79.9 [*C*(CH_3)$_3$], 108.3 (Isox C-4), 150.2 (Isox C-3), 154.8 (*N-Boc* C=O), 162.0 (C=O), 179.5 (Isox C-5). ^{15}N NMR (71 MHz, $CDCl_3$): δ -294.7 (*N*-Boc), -3.1 (*N*-Isox). HRMS (ESI), m/z: calcd. for $C_{15}H_{22}N_2O_5Na^+$ 333.1421 $[M+Na]^+$; found 333.1423.

4. Methyl 5-[1-(*tert*-butoxycarbonyl)azetidin-3-yl]-^{15}N-1,2-oxazole-4-carboxylate (5)

To a solution of *tert*-butyl 3-[3-(dimethylamino)-2-(methoxycarbonyl)prop-2-enoyl]azetidine-1-carboxylate (**3a**) (0.2 g, 0.64 mmol) in ethanol (7 mL) was added ^{15}N hydroxylamine hydrochloride (0.1 g, 0.77 mmol) and the reaction mixture was stirred at rt for 4 h. The solvent was removed at reduced pressure and the residue was subjected to flash column chromatography on silica gel (eluent: Hex/ EtOAc 8:1) to afford the title compound **5** as colorless oil (0.12 g, 67%). IR, (ν_{max}, cm^{-1}): 3115 (CH-arom), 2968 (CH-aliph), 1723 (C=O), 1688 (C=O), 1409, 1138, 857, 779. ^{1}H NMR (700 MHz, $CDCl_3$): δ 1.46 (s, 9H, 3 × CH_3), 3.87 (s, 3H, O-CH_3), 4.24 (dd, *J* = 8.8, 6.6 Hz, 2H, Az 2,4-H), 4.32 (t, *J* = 8.8 Hz, 2H, Az 2,4-H), 4.48 (tt, *J* = 9.0, 6.5 Hz, 1H, Az 3-H), 8.52 (d, $^2J_{H,N}$ = 14.4 Hz, 1H, Isox 3-H). ^{13}C NMR (176 MHz, $CDCl_3$): δ 26.0 (Az C-3), 28.4 (3×*C*H_3), 52.1 (O-*C*H_3), 52.9 (Az C-2,4), 80.1 [*C*(CH_3)$_3$], 109.8 (d, $^2J_{C,N}$ = 1.30 Hz, Isox C-4), 150.4 (d, $^1J_{C,N}$ = 4.55 Hz, Isox C-3), 156.0 (*N*-Boc C=O), 161.5 (C=O), 175.3 (d, $^2J_{C,N}$ = 1.96 Hz, Isox C-5). ^{15}N NMR (71 MHz, $CDCl_3$): δ -312.2 (*N*-Boc), -1.5 (*N*-Isox). HRMS (ESI), m/z: calcd. for $C_{13}H_{18}{}^{15}NNO_5Na^+$ 306.1139 $[M+Na]^+$; found 306.1139.

5. Synthesis of compounds 6a,b

5.1. (2*R*)-2-[4-(Methoxycarbonyl)-1,2-oxazol-5-yl]pyrrolidin-1-ium trifluoroacetate (6a)

To a compound **4b** (0.20 g, 0.70 mmol) in dichloromethane (2.0 mL) was added trifluoroacetic acid (2.0 mL) and the mixture was stirred at rt for 1.0 h. After removal of the solvent *in vacuo* the residue were washed with acetone and dried to afford the title compound **6a** as whitish resin (0.12g, 90%). IR, (v_{max}, cm^{-1}): 2963 (CH-arom), 2725 (CH-aliph), 1727 (C=O), 1665 (C=O). ^{1}H NMR (700 MHz, $CDCl_3$): δ 2.21–2.42 (m, 3H, Pyr 4-H, Pyr 3-H_a), 2.50–2.58 (m, 1H, Pyr 3-H_b), 3.60 (m, 2H, Pyr 5-H), 3.88 (s, 3H, O-CH_3), 5.43 (t, *J* = 7.4 Hz, 1H, Pyr 2-H), 8.52 (s, 1H, Isox 3-H). ^{13}C NMR (176 MHz, $CDCl_3$) δ 24.2 (Pyr C-4), 29.5 (Pyr C-3), 45.8 (Pyr C-5), 52.6 (O-CH_3), 53.3 (Pyr C-2), 111.8 (Isox C-4), 116.3 (q, $^{1}J_{C,F}$ = 291.4 Hz, CF_3), 150.3 (Isox C-3), 161.4 (C=O), 162.3 (q, $^{2}J_{C,F}$ = 35.4 Hz, C=O, TFA), 169.8 (Isox C-5). ^{15}N NMR (71 MHz, $CDCl_3$): δ -325.0 (NH_2^+), 3.8 (*N*-Isox). HRMS (ESI), m/z: calcd. for $C_9H_{13}N_2O_3$ 197.0921 [M – CF_3COO^-]$^+$; found 197.0921.

5.2. (2*S*)-2-[4-(Methoxycarbonyl)-1,2-oxazol-5-yl]piperidin-1-ium trifluoroacetate (6b)

To a compound **4g** (0.31 g, 1.0 mmol) in dichloromethane (2.0 mL) was added trifluoroacetic acid (2.0 mL) and the mixture was stirred at rt for 1.0 h. After removal of the solvent *in vacuo*, the residue was kept at 5°C, the formed crystals were washed with acetone and dried to afford the title compound **6b** as whitish crystals (0.28g, 88%), mp 139–140°C. IR, (v_{max}, cm^{-1}): 3071 (CH-arom), 2965 (CH-aliph), 1728 (C=O), 1658 (C=O). ^{1}H NMR (700 MHz, $CDCl_3$): δ 1.71–1.79 (m, 1H, Pip 5-H_a), 1.91–2.25 (m, 5H, Pip 3-CH_2, 4-CH_2, 5-CH_b), 3.23 (t, *J* = 11.0 Hz, 1H, Pip 6-H_a), 3.67 (d, *J* = 12.8 Hz, 1H, Pip 6-H_b), 3.91 (s, 3H, O-CH_3), 4.95 (d, *J* = 9.7 Hz, 1H, Pip 2-H),

8.53 (s, 1H, Isox 3-H), 9.04 (s, 1H, NH_2^+), 10.00 (s, 1H, NH_2^+). ^{13}C NMR (176 MHz, $CDCl_3$): δ 21.7 and 21.8 (Pip C-4 and C-5), 27.0 (Pip C-3), 45.4 (Pip C-6), 52.6 (Pip C-2), 53.0 (O-CH_3), 111.2 (Isox C-4), 115.5 (q, $^1J_{C,F}$ = 288.6 Hz, CF_3), 150.1 (Isox C-3), 161.1 (q, $^2J_{C,F}$ = 38.7 Hz, C=O, TFA), 162.3 (C=O), 170.4 (Isox C-5). ^{15}N NMR (71 MHz, $CDCl_3$): δ -335.6 (NH_2^+), 4.0 (*N*-Isox). HRMS (ESI), m/z: calcd. for $C_{10}H_{15}N_2O_3$ 211.1077 [M – CF_3COO^-]$^+$; found 211.1077.

6. ^1H, ^{13}C and ^{15}N NMR spectra of compounds 3a-h, 4a-h, 5, 6a,b

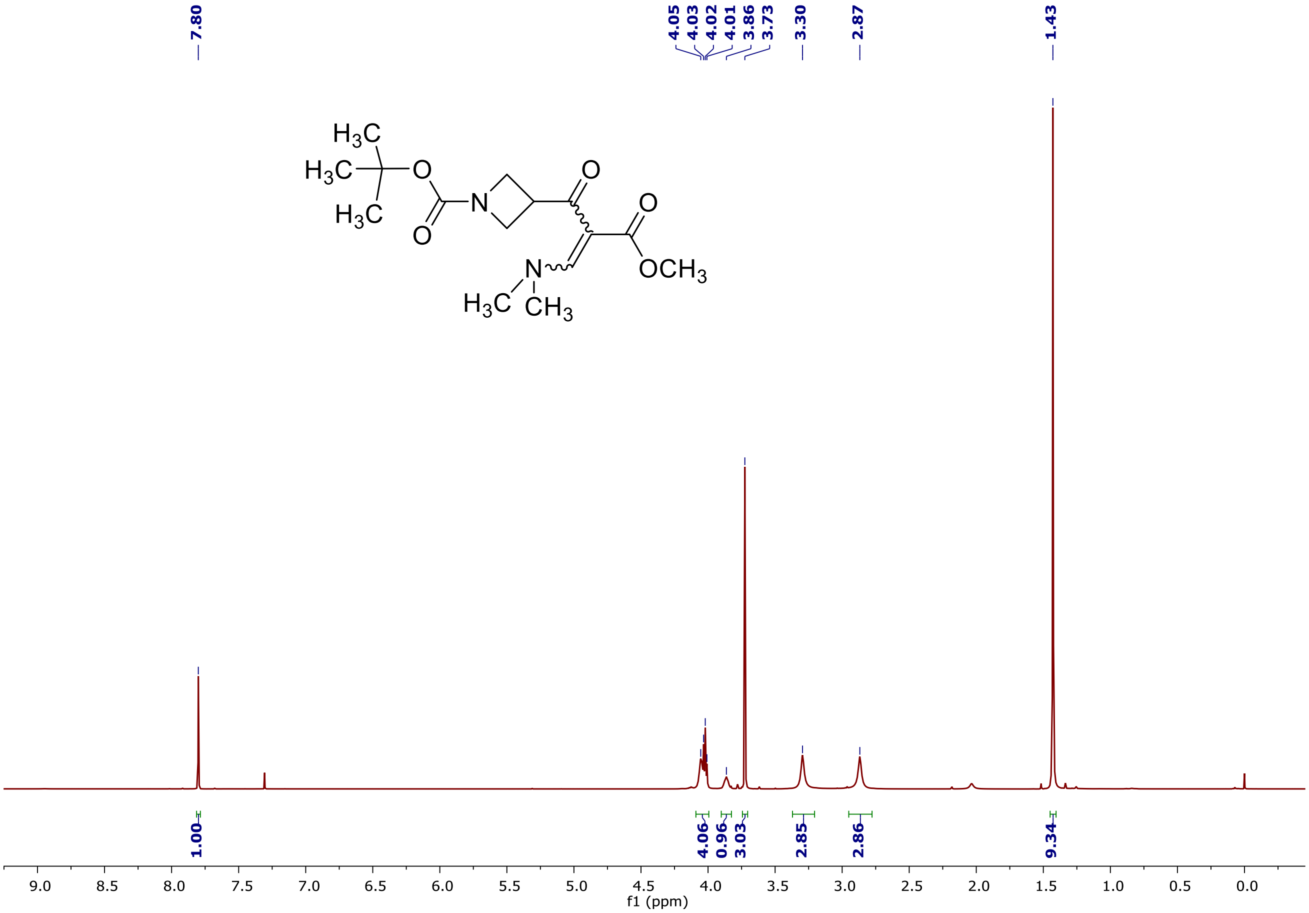

Figure S1: ^1H NMR of **3a**, ($CDCl_3$, 700 MHz).

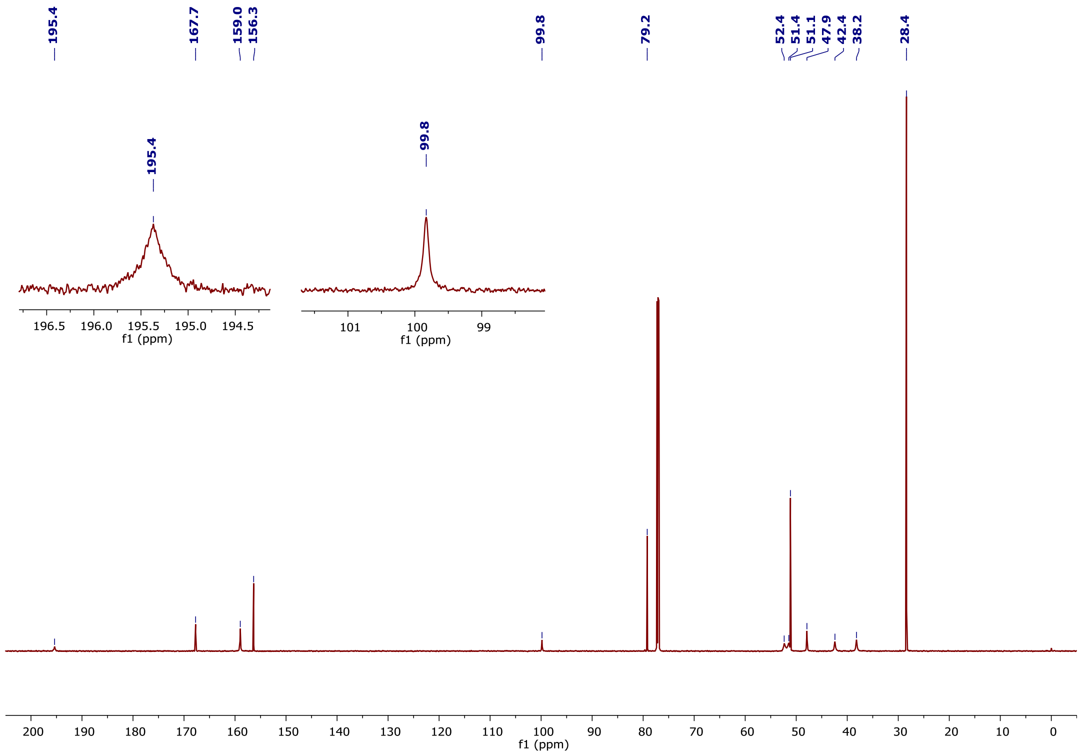

Figure S2: ^{13}C NMR of **3a**, ($CDCl_3$, 176 MHz).

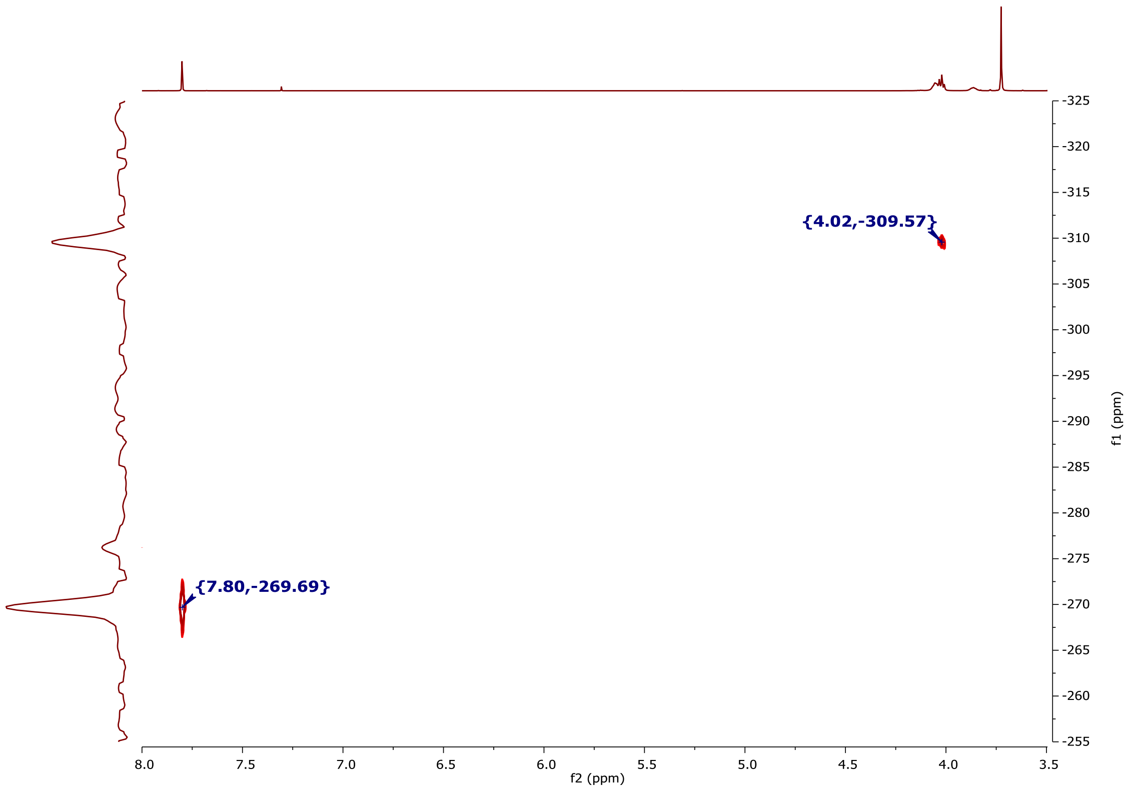

Figure S3: ^{15}N NMR of **3a**, ($CDCl_3$, 71 MHz).

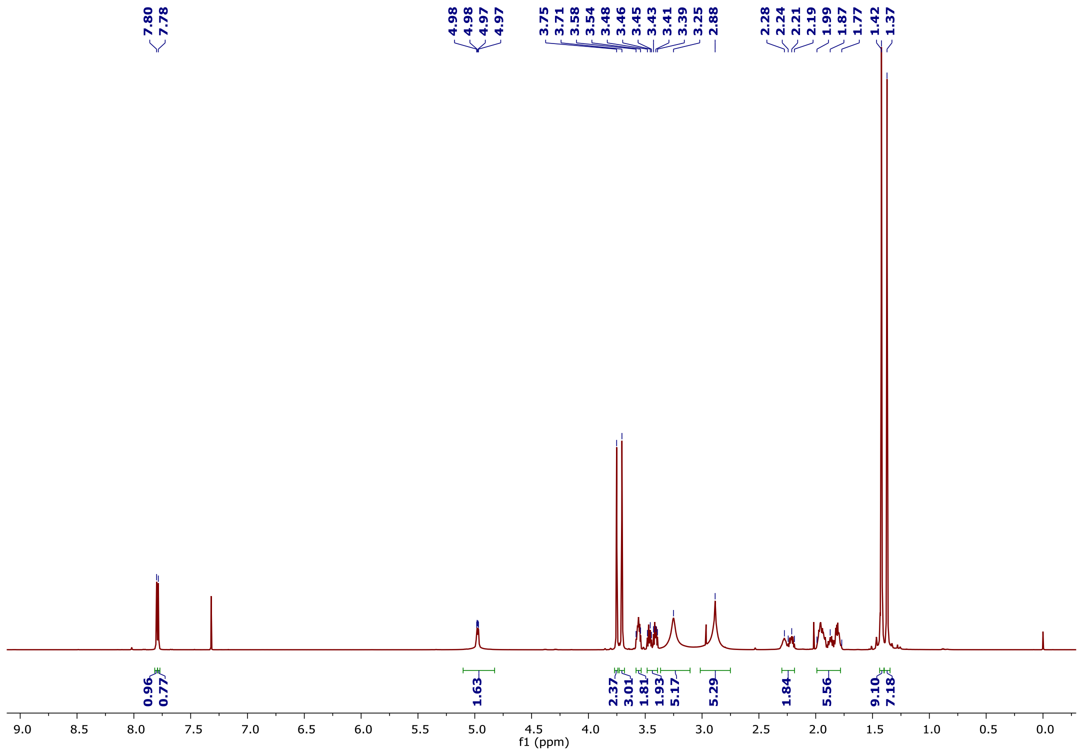

Figure S4: ^{1}H NMR of **3b**, ($CDCl_3$, 700 MHz).

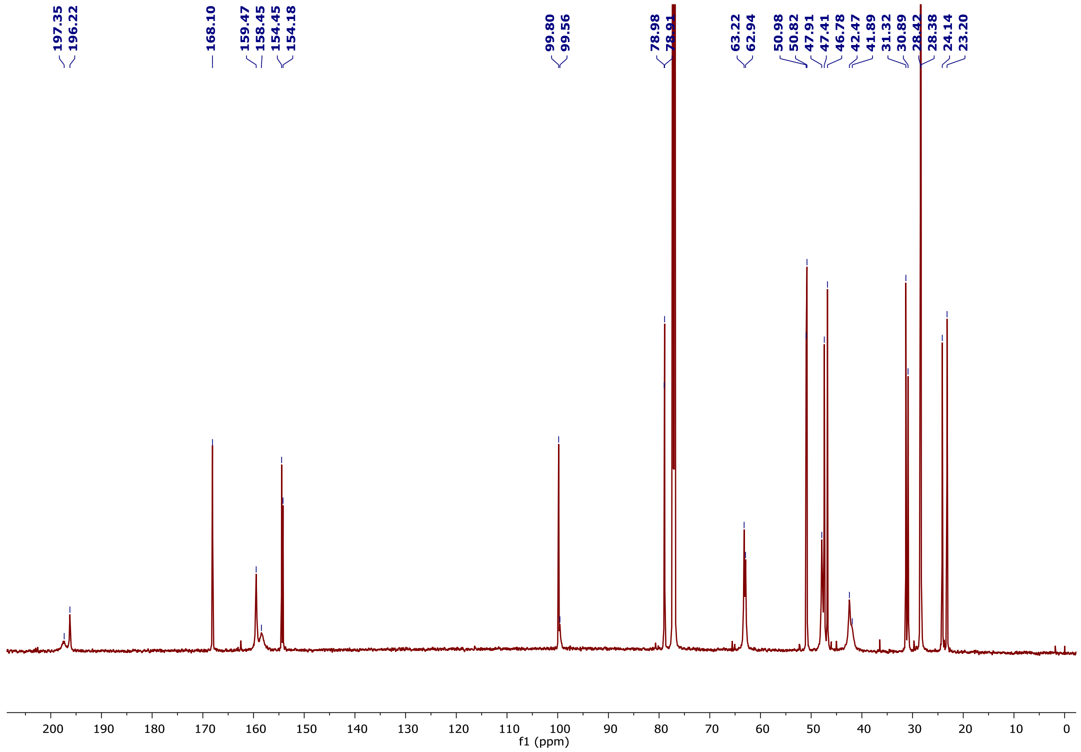

Figure S5: ^{13}C NMR of **3b**, ($CDCl_3$, 176 MHz).

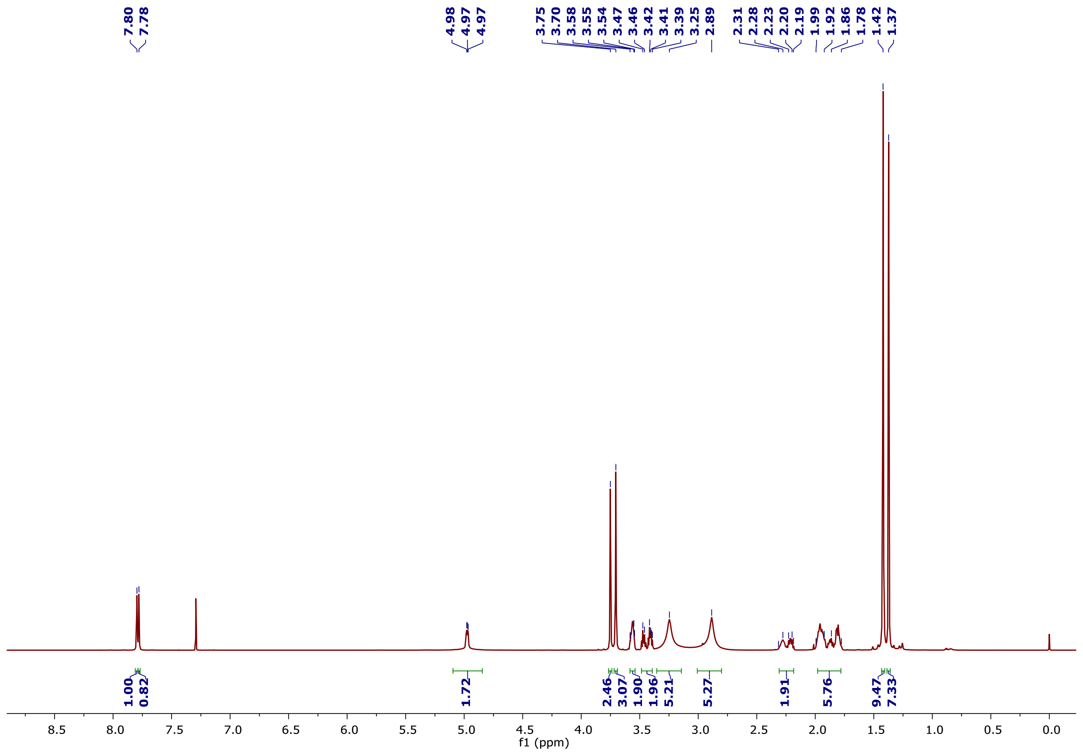

Figure S6: ^{1}H NMR of **3c**, ($CDCl_3$, 700 MHz).

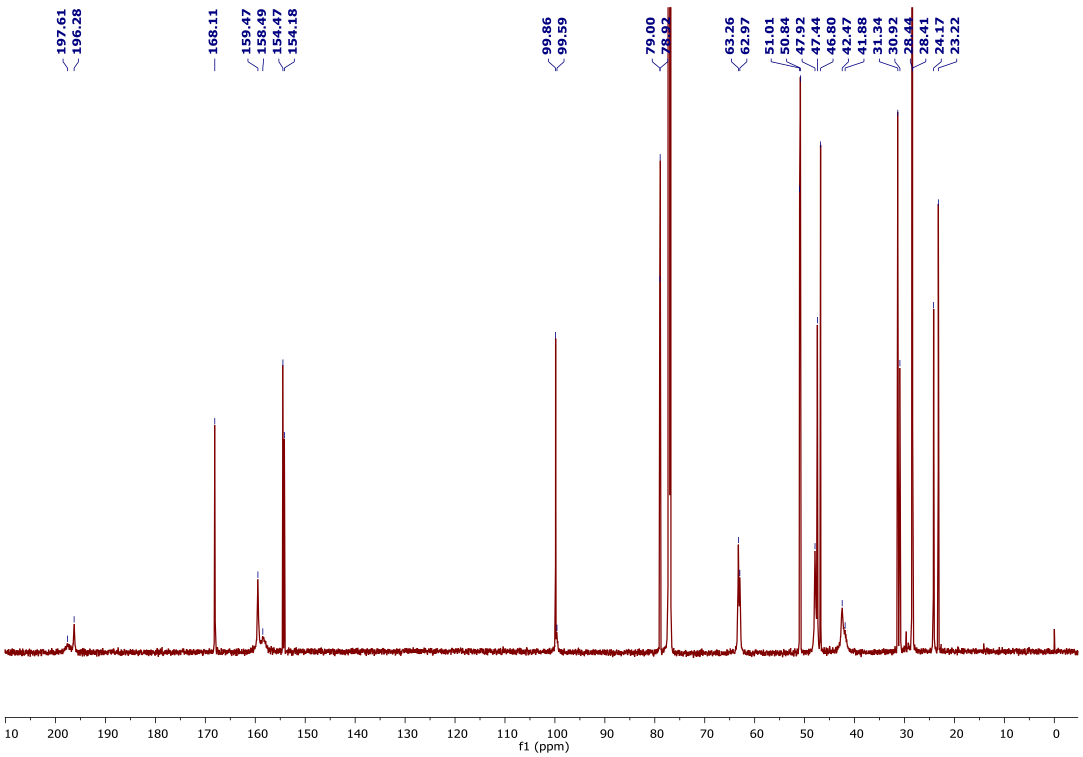

Figure S7: ^{13}C NMR of **3c**, ($CDCl_3$, 176 MHz).

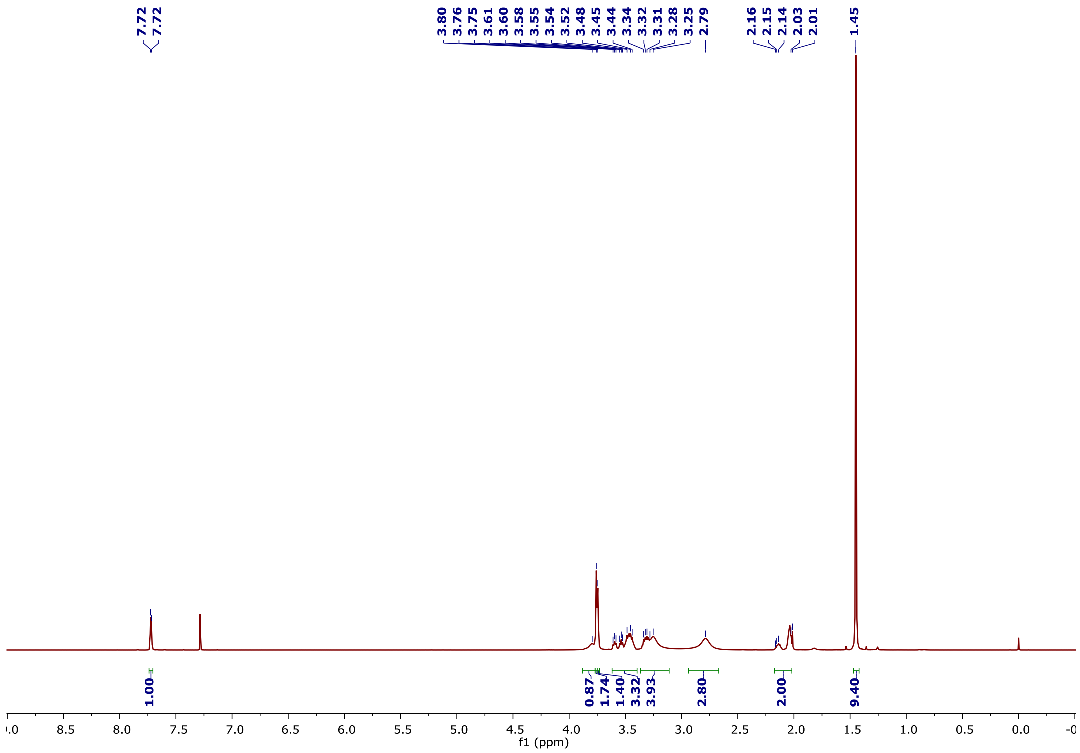

Figure S8: ^{1}H NMR of **3d**, ($CDCl_3$, 700 MHz).

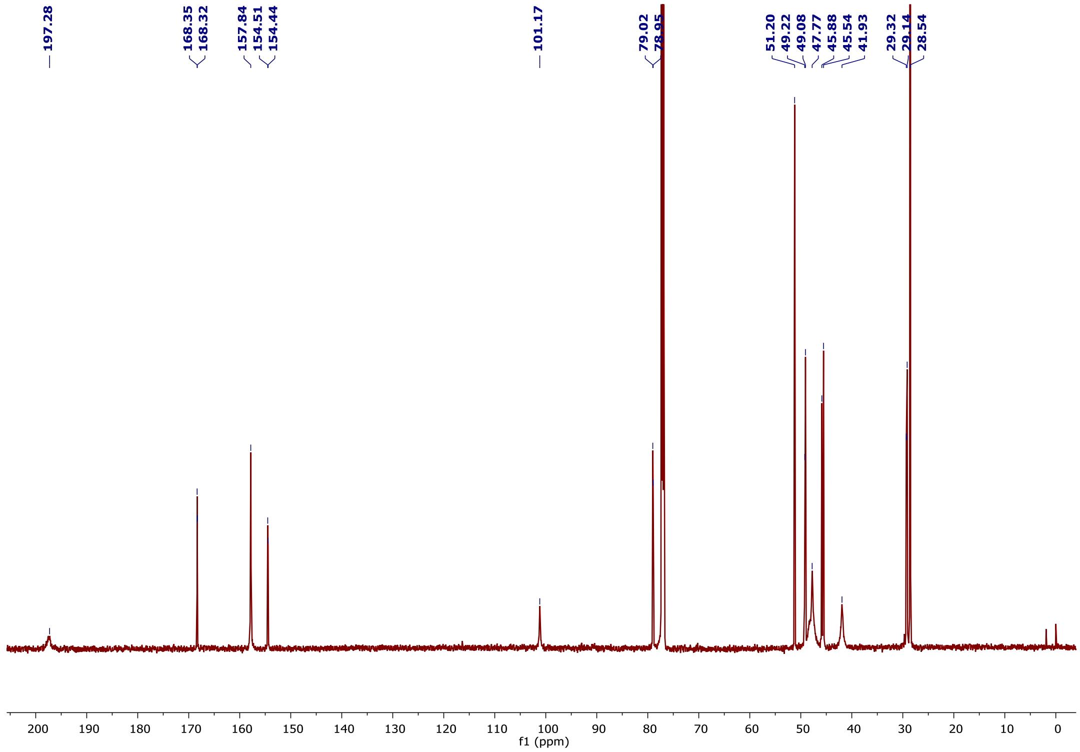

Figure S9: ^{13}C NMR of **3d**, ($CDCl_3$, 176 MHz).

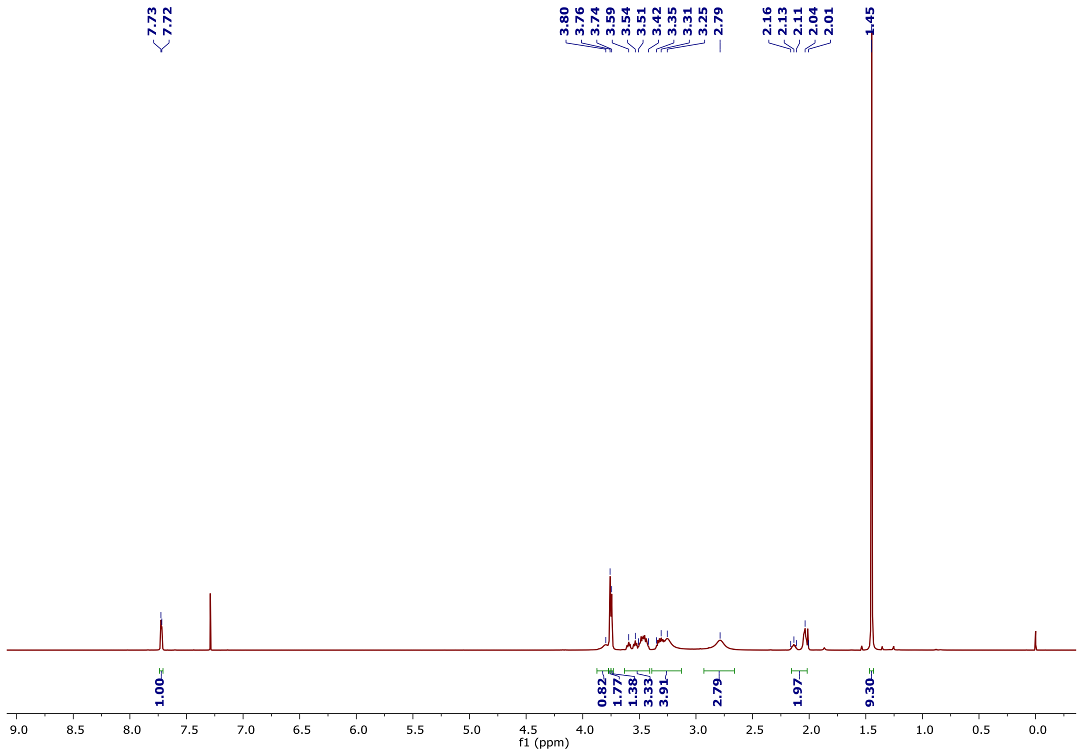

Figure S10: ^{1}H NMR of **3e**, ($CDCl_3$, 700 MHz).

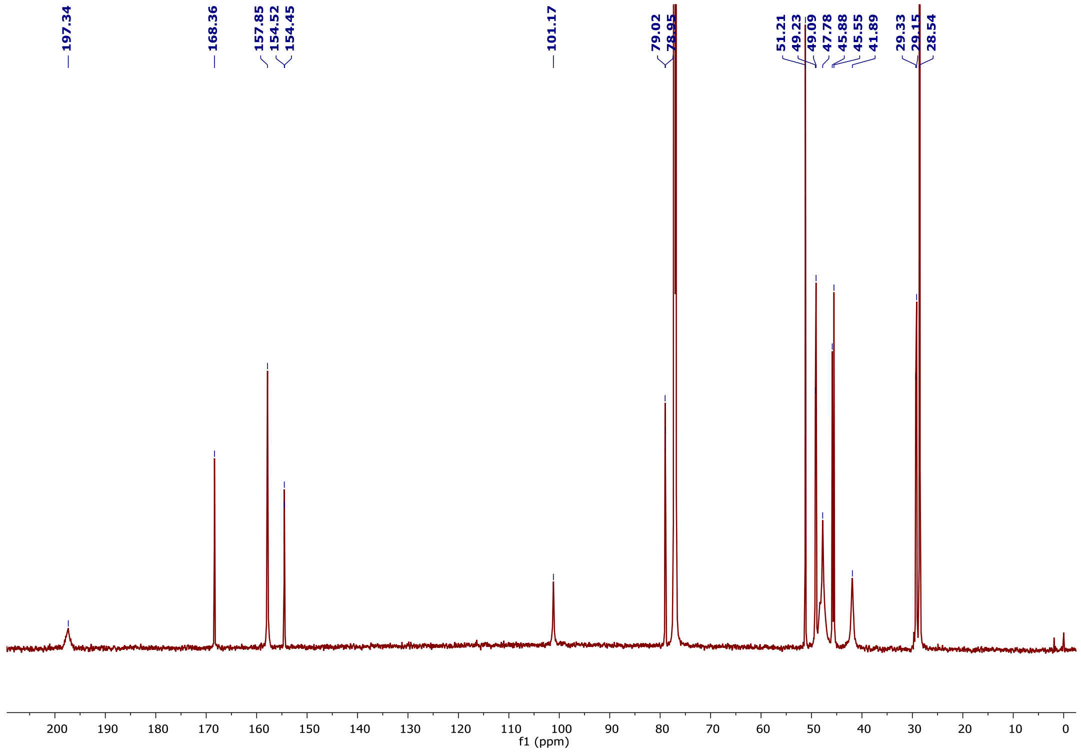

Figure S11: ^{13}C NMR of **3e**, ($CDCl_3$, 176 MHz).

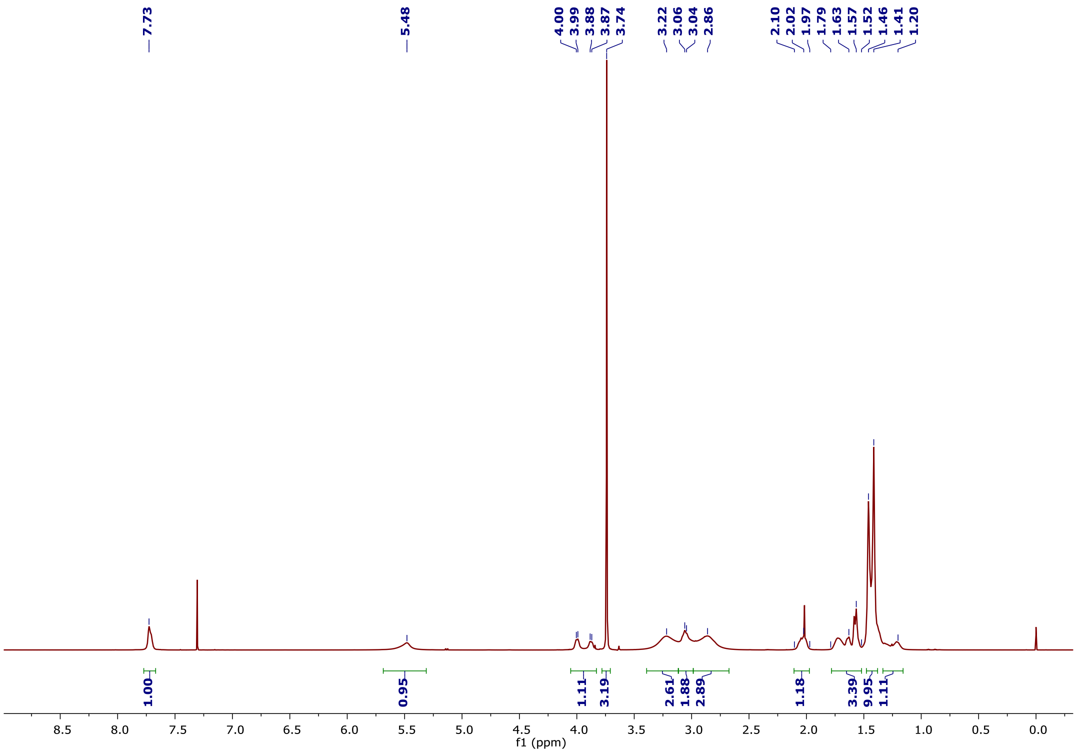

Figure S12: ^{1}H NMR of **3f**, ($CDCl_3$, 700 MHz).

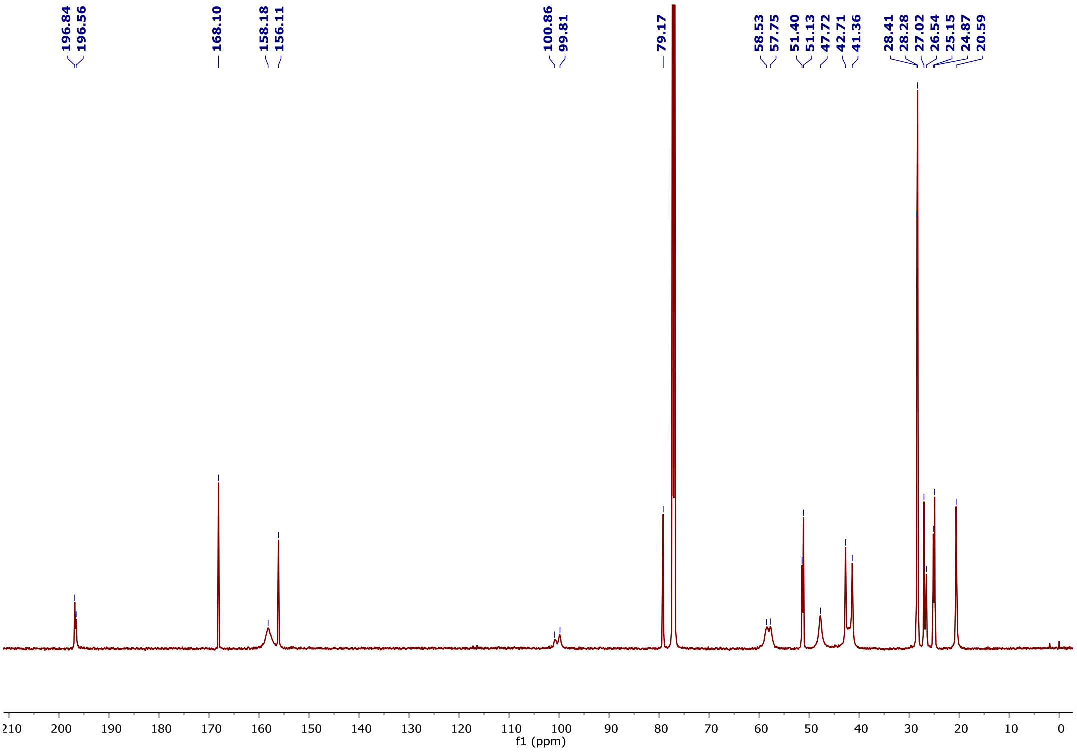

Figure S13: ^{13}C NMR of **3f**, ($CDCl_3$, 176 MHz).

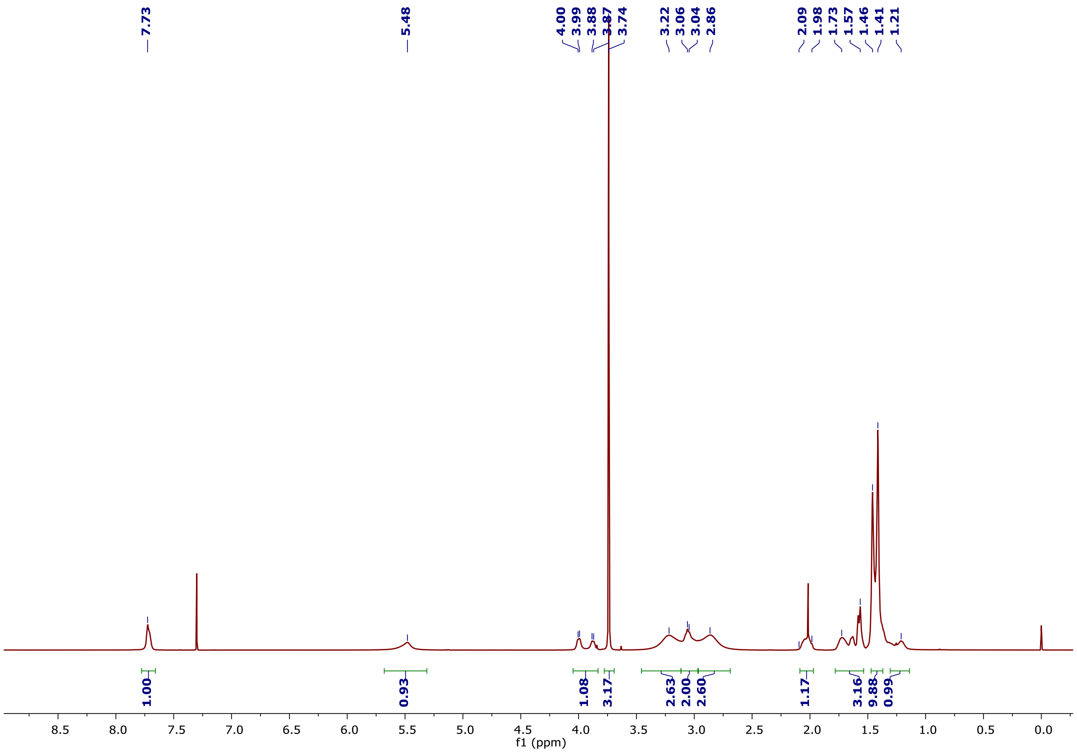

Figure S14: ^{1}H NMR of **3g**, ($CDCl_3$, 700 MHz).

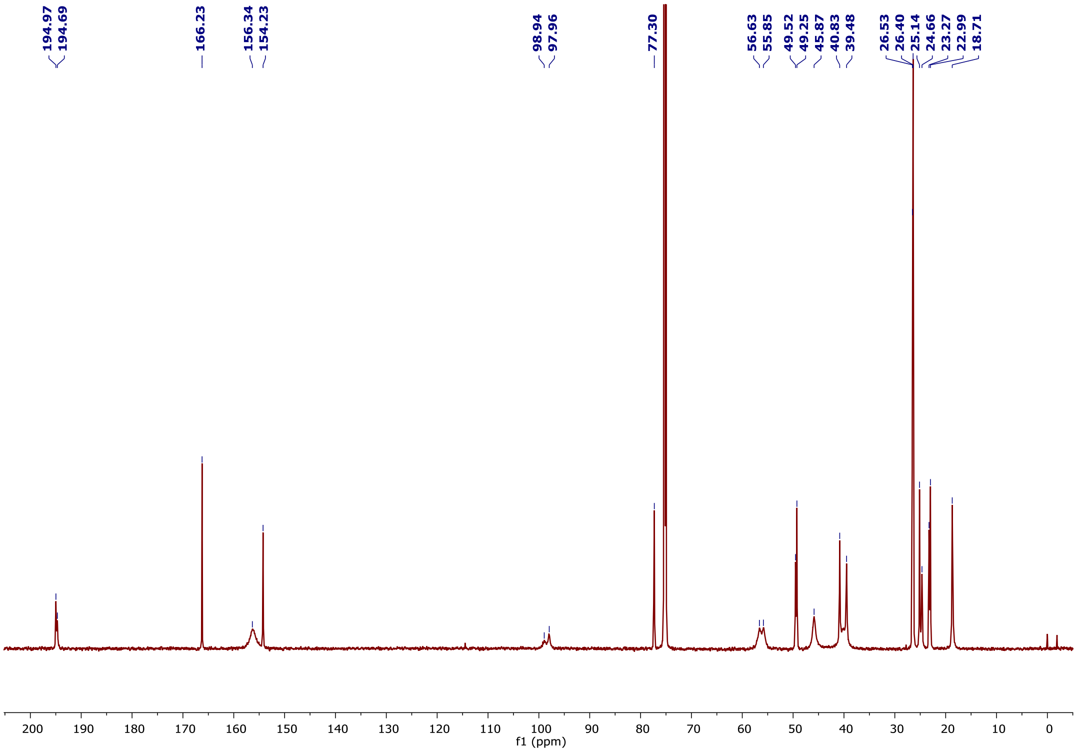

Figure S15: ^{13}C NMR of **3g**, ($CDCl_3$, 176 MHz).

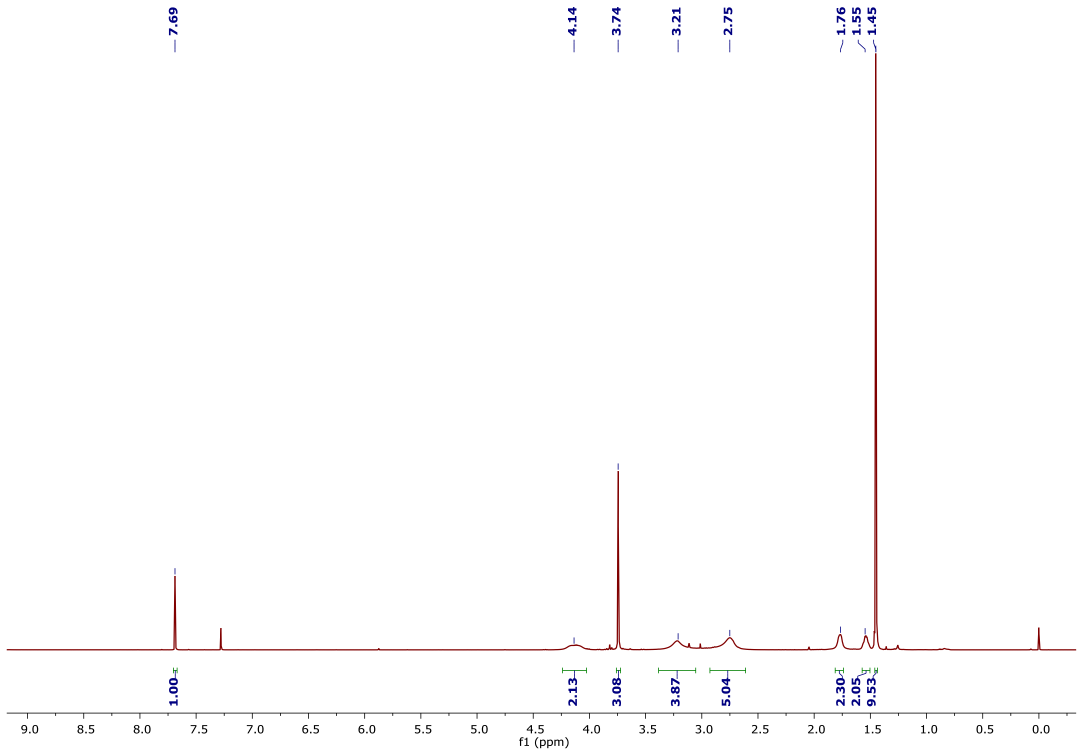

Figure S16: ^{1}H NMR of **3h**, ($CDCl_3$, 700 MHz).

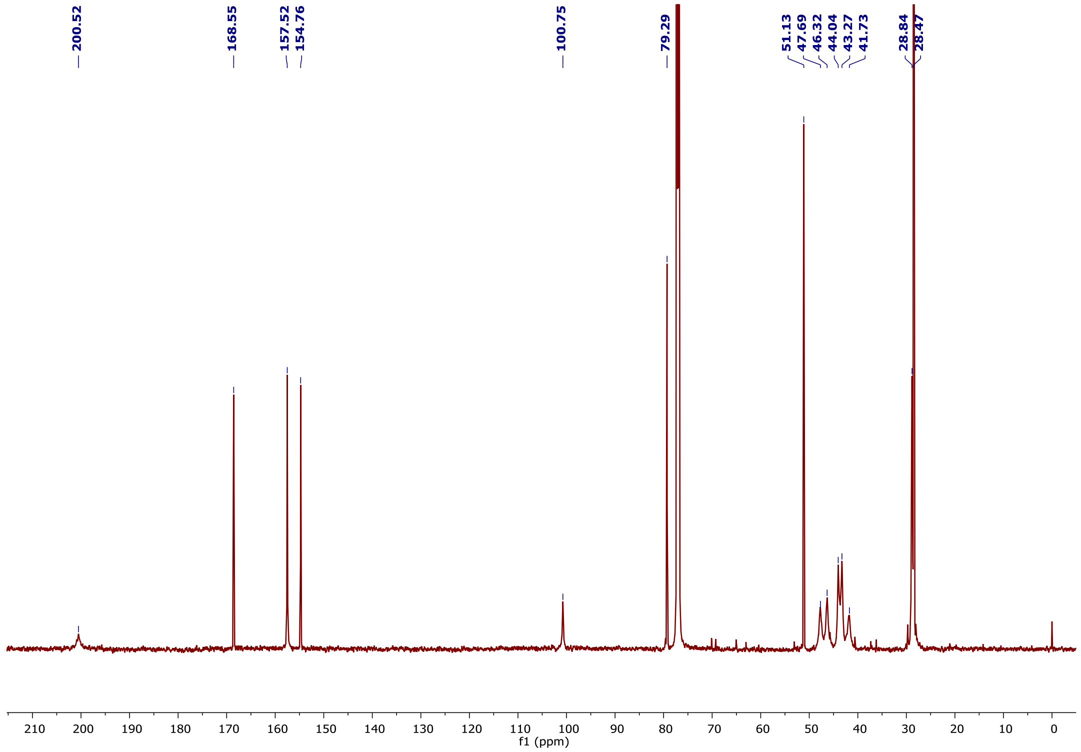

Figure S17: ^{13}C NMR of **3h**, ($CDCl_3$, 176 MHz).

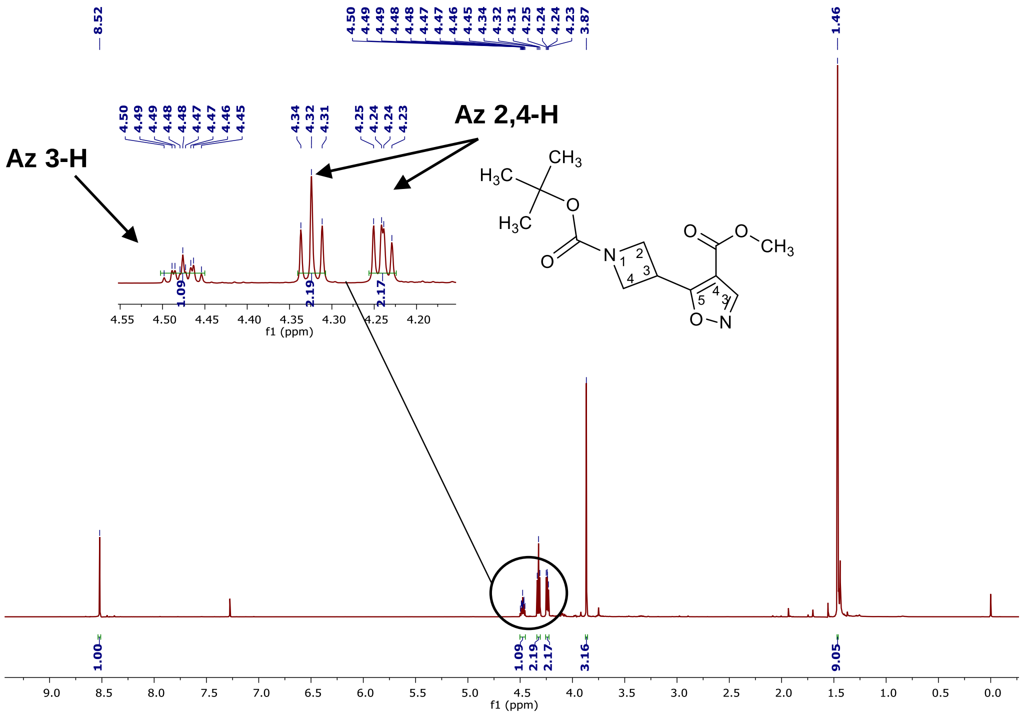

Figure S18: ^{1}H NMR of **4a**, ($CDCl_3$, 700 MHz).

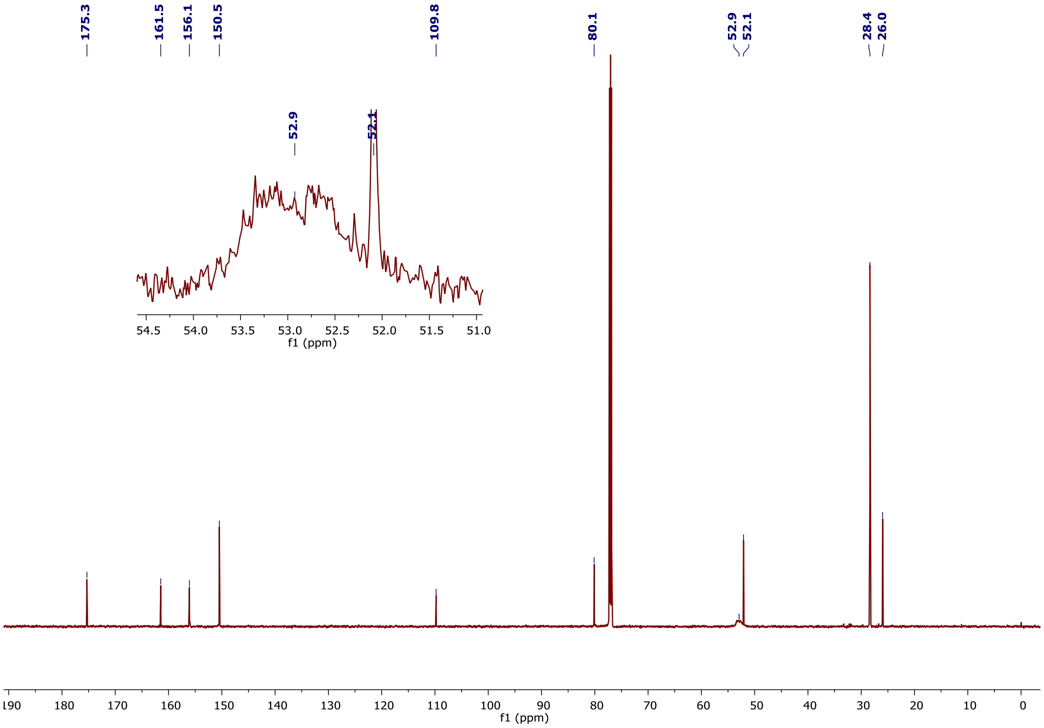

Figure S19: ^{13}C NMR of **4a**, ($CDCl_3$, 176 MHz).

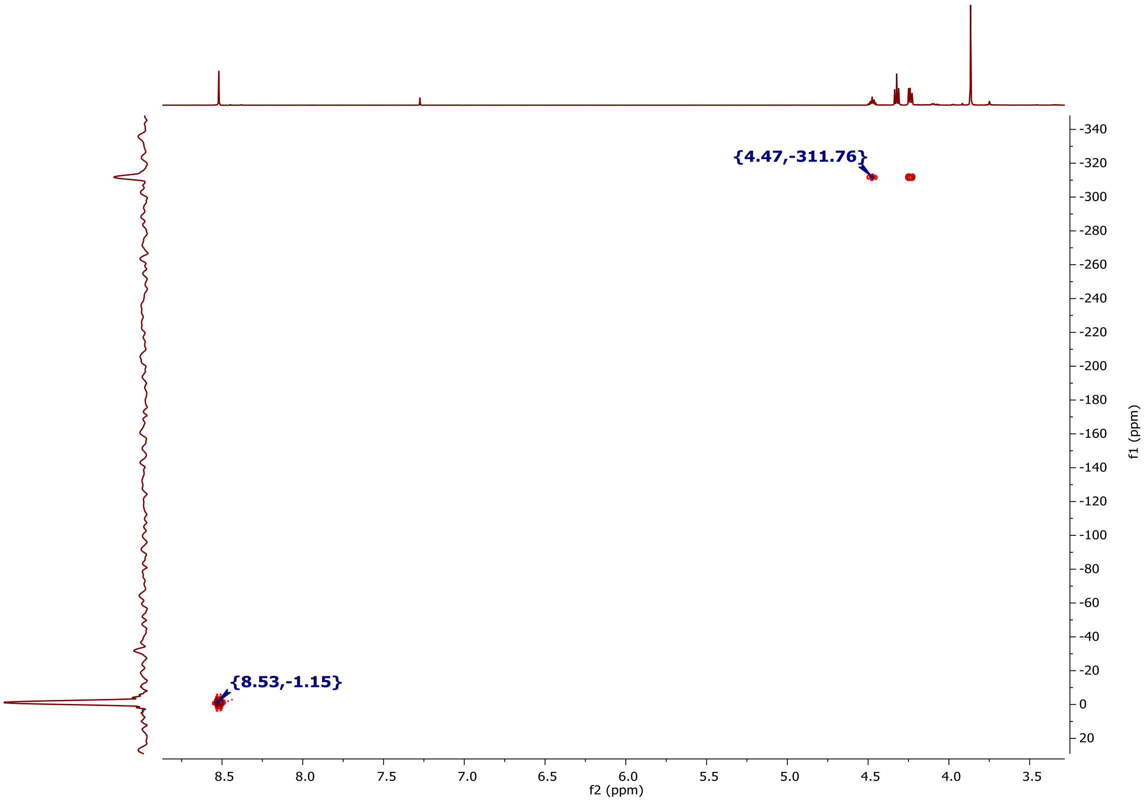

Figure S20: ^{15}N NMR of **4a**, ($CDCl_3$, 71 MHz).

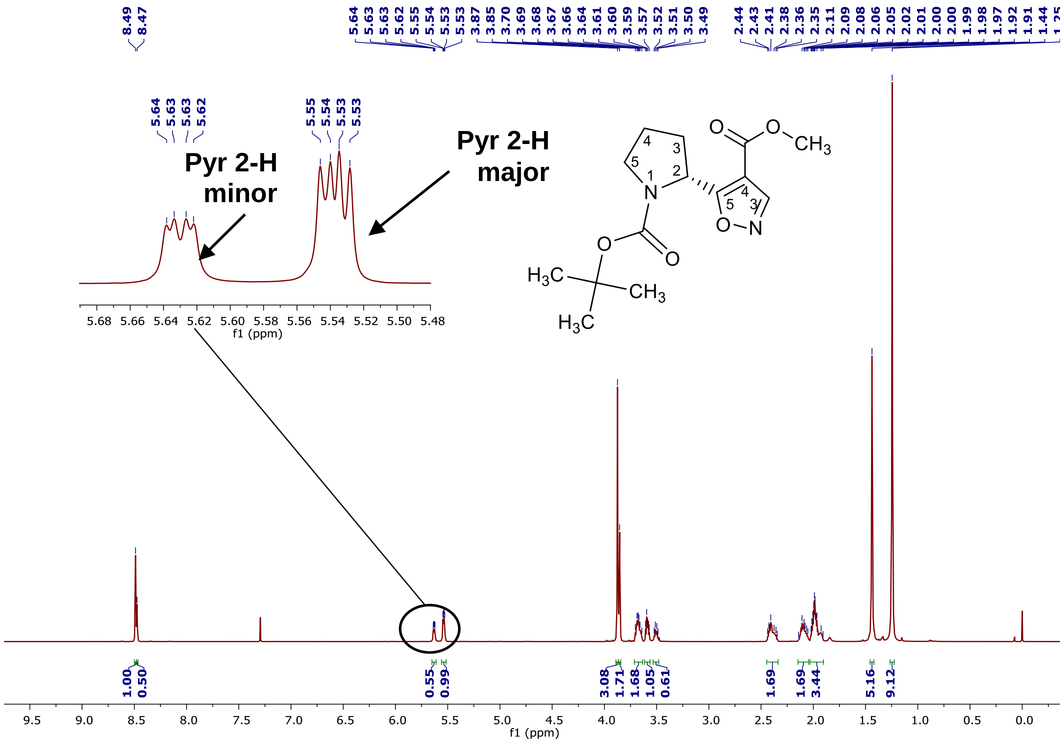

Figure S21: ^{1}H NMR of **4b**, ($CDCl_3$, 700 MHz).

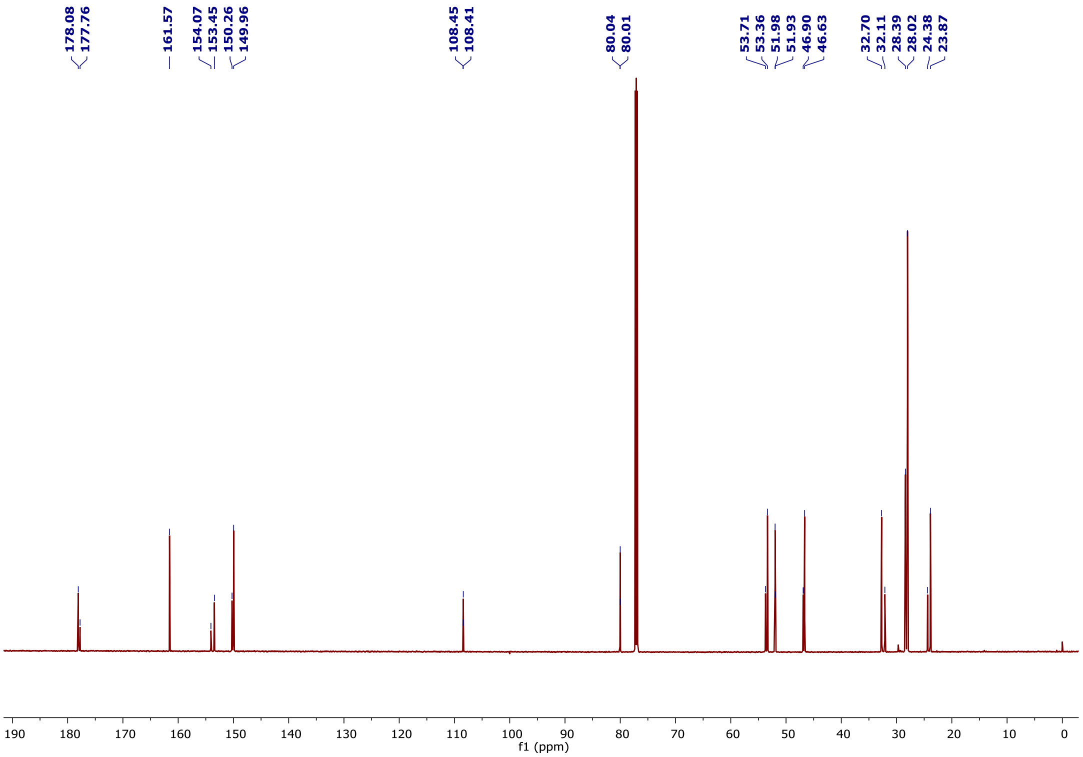

Figure S22: ^{13}C NMR of **4b**, ($CDCl_3$, 176 MHz).

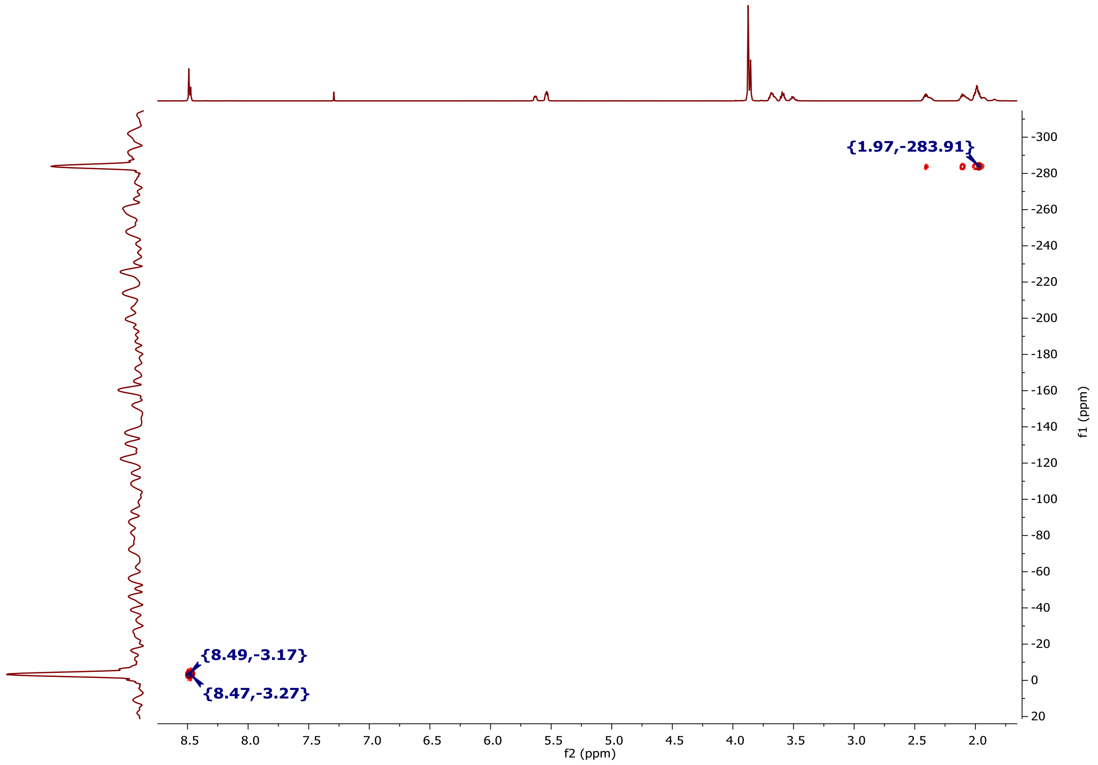

Figure S23: ^{15}N NMR of **4b**, ($CDCl_3$, 71 MHz).

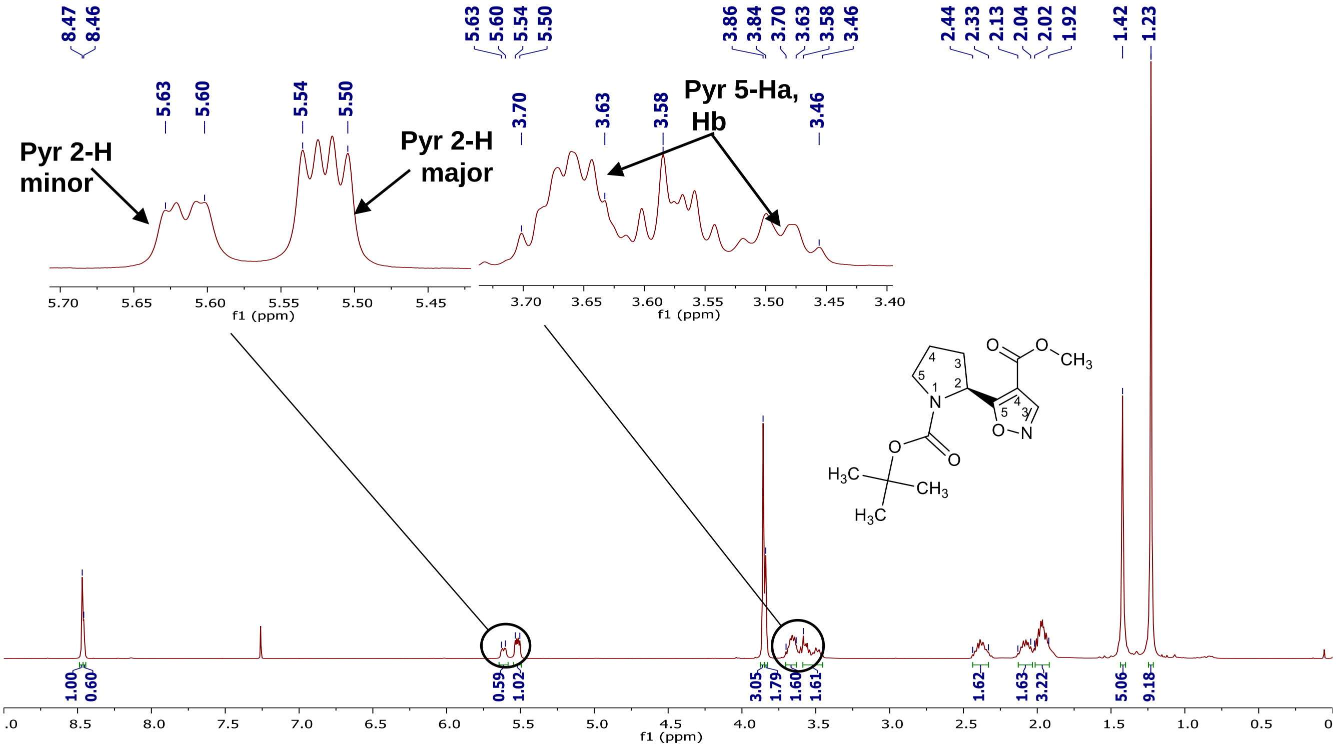

Figure S24: ^{1}H NMR of **4c**, ($CDCl_3$, 400 MHz).

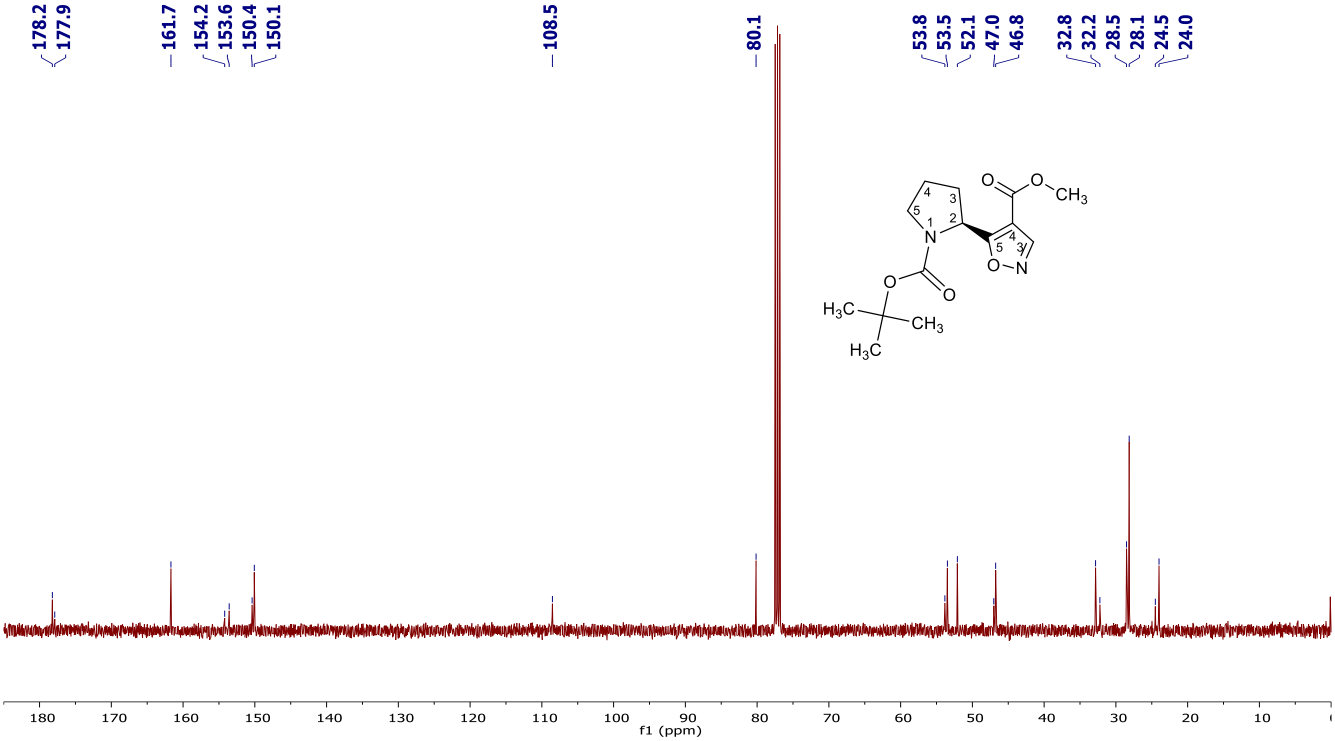

Figure S25: ^{13}C NMR of **4c**, ($CDCl_3$, 100 MHz).

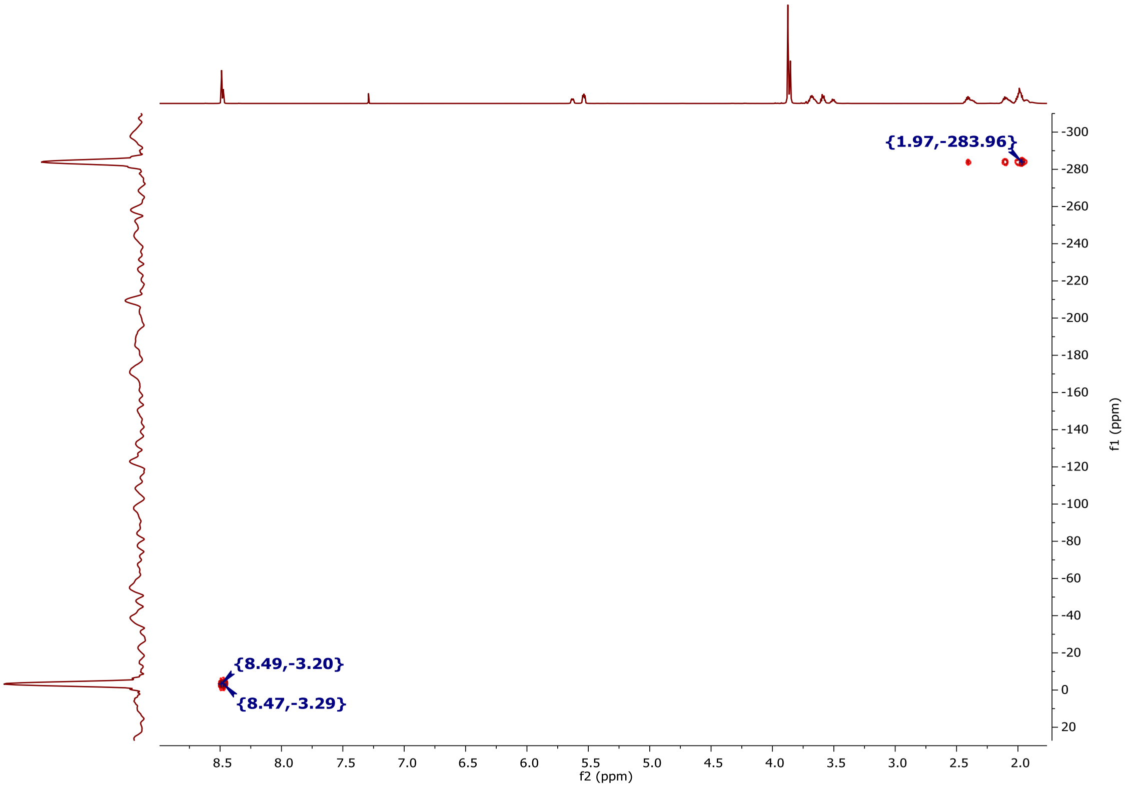

Figure S26: ^{15}N NMR of **4c**, ($CDCl_3$, 71 MHz).

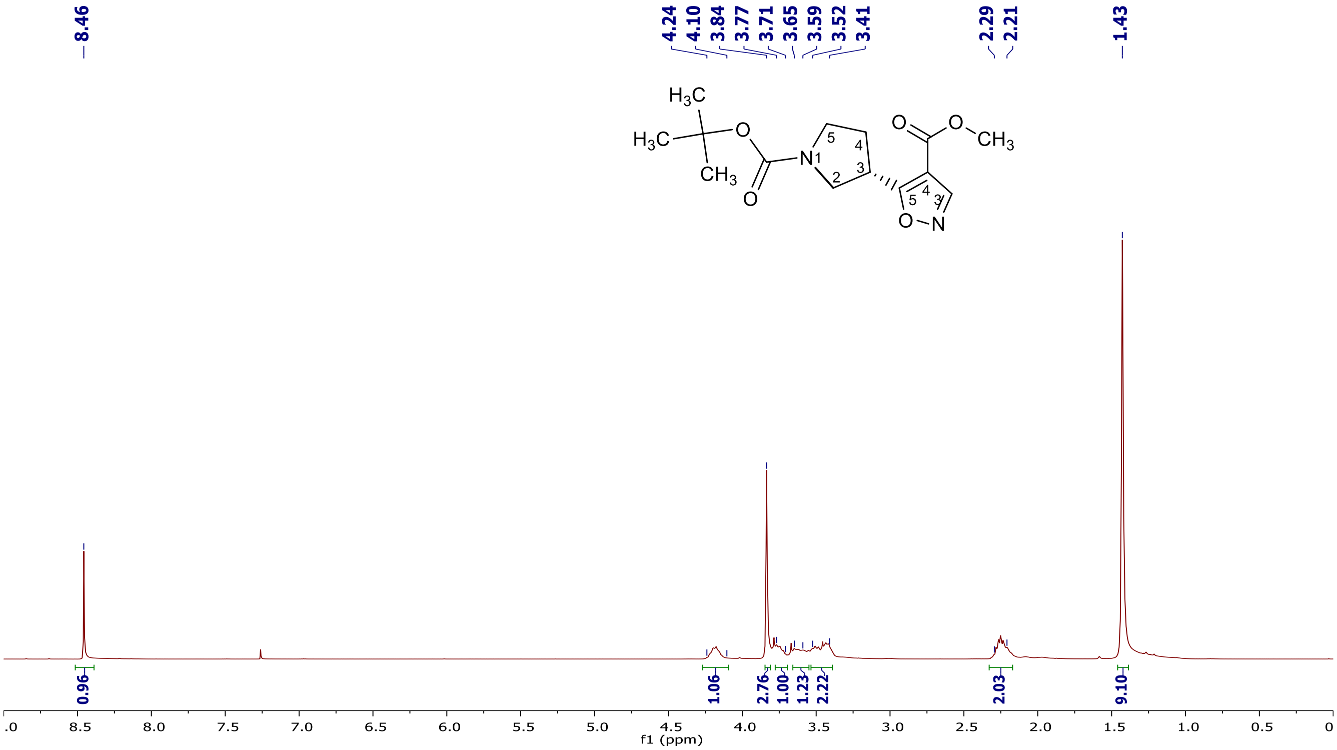

Figure S27: ^{1}H NMR of **4d**, ($CDCl_3$, 400 MHz).

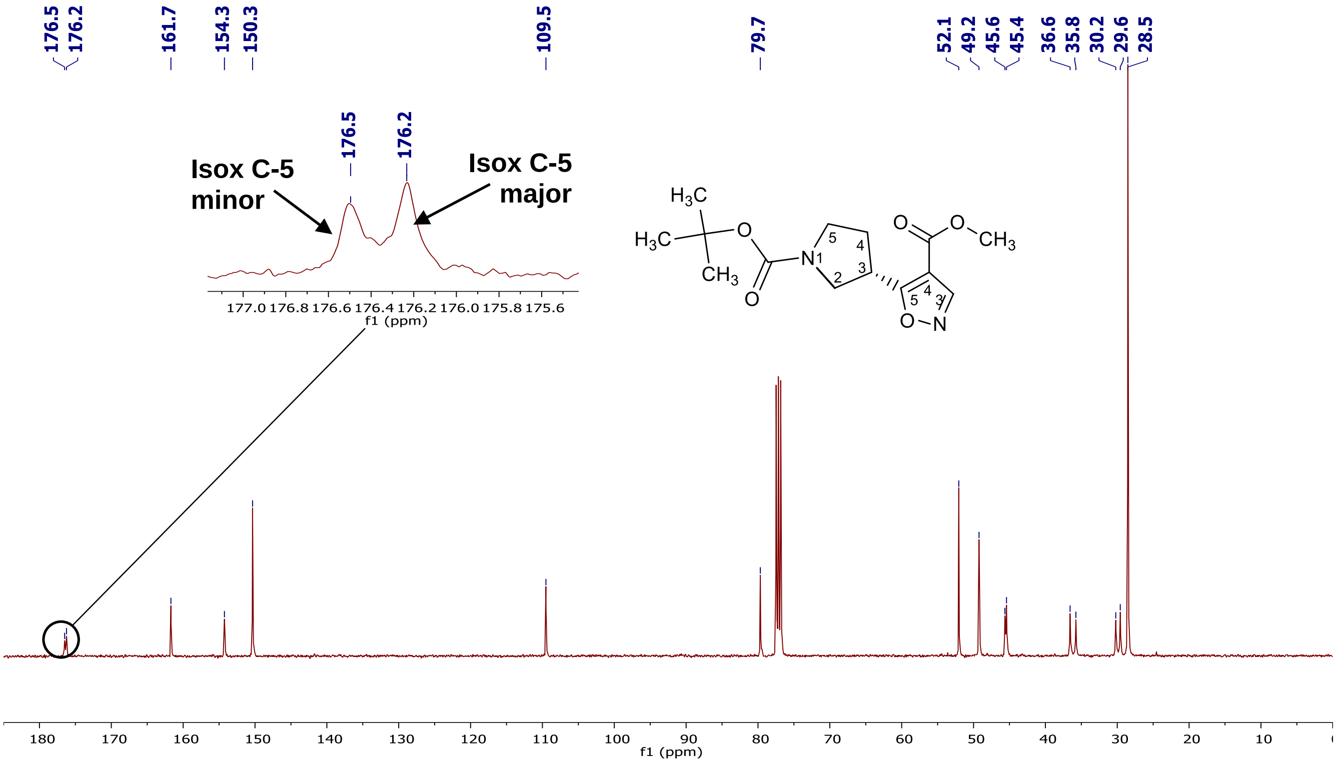

Figure S28: ^{13}C NMR of **4d**, ($CDCl_3$, 100 MHz).

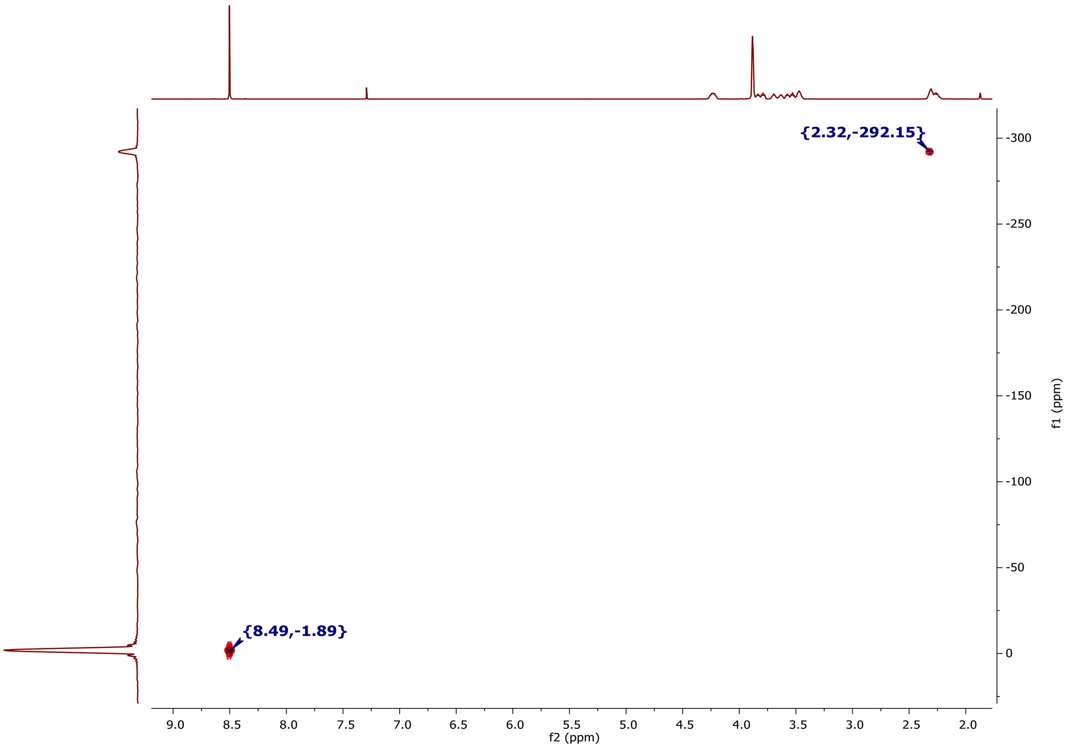

Figure S29: ^{15}N NMR of **4d**, ($CDCl_3$, 71 MHz).

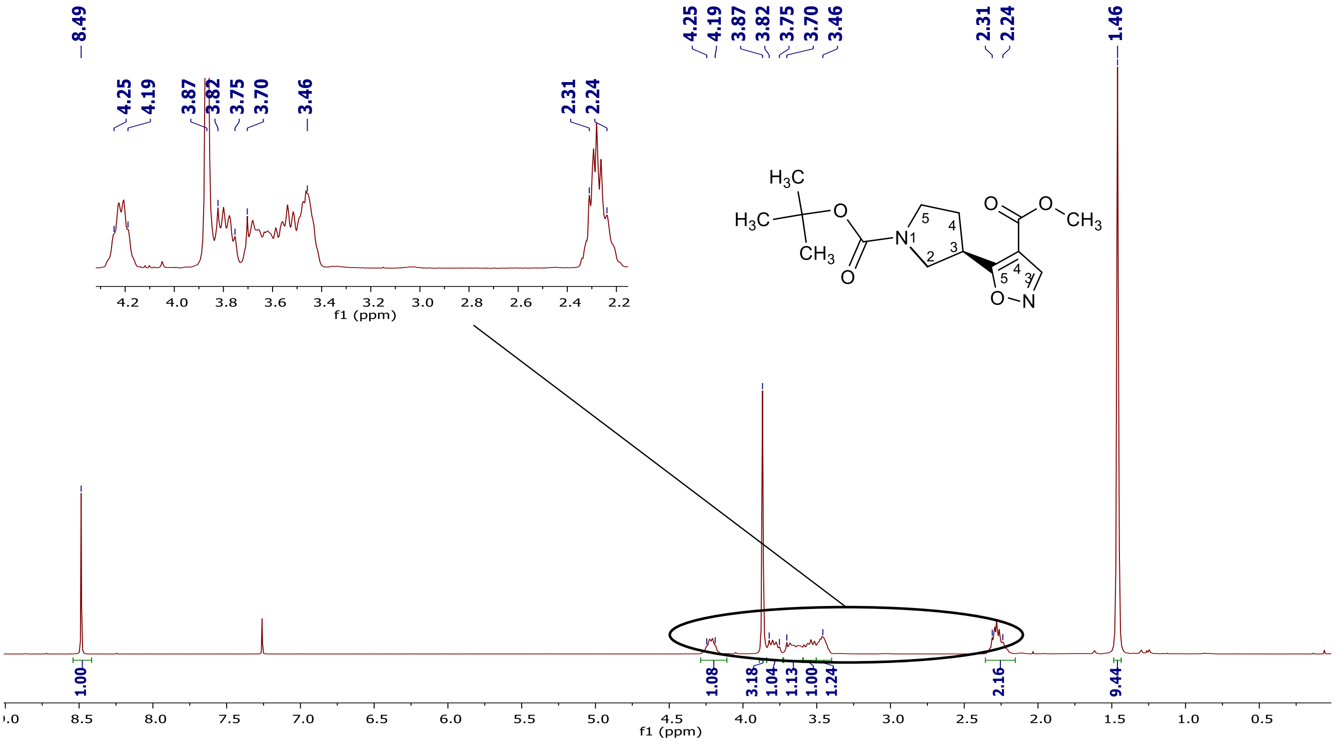

Figure S30: ^{1}H NMR of **4e**, ($CDCl_3$, 400 MHz).

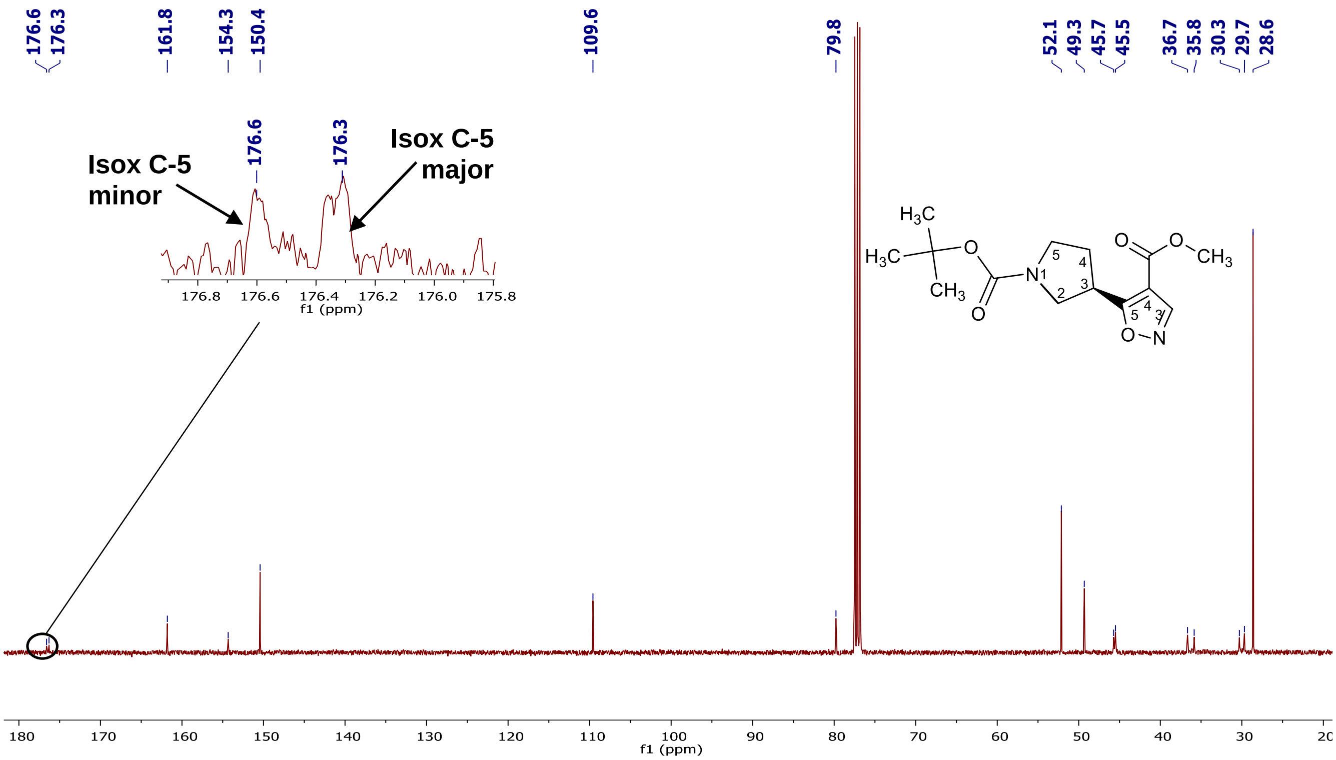

Figure S31: ^{13}C NMR of **4e**, ($CDCl_3$, 100 MHz).

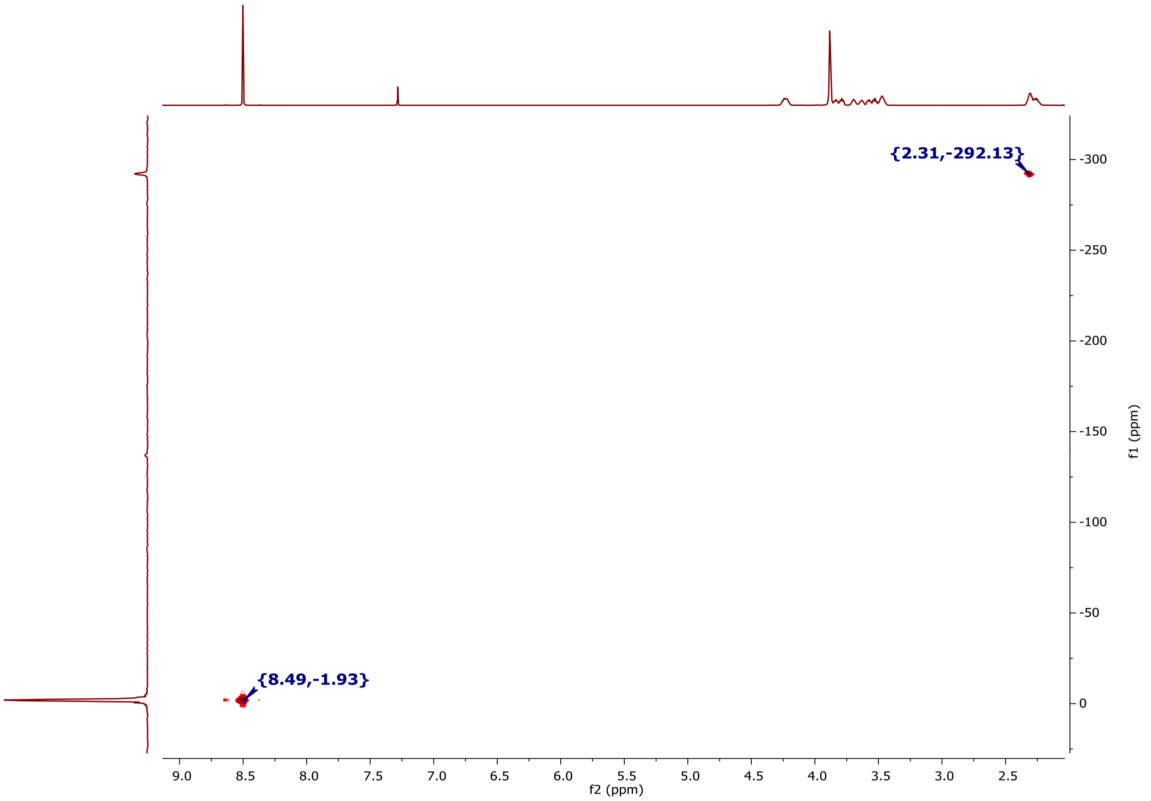

Figure S32: ^{15}N NMR of **4e**, ($CDCl_3$, 71 MHz).

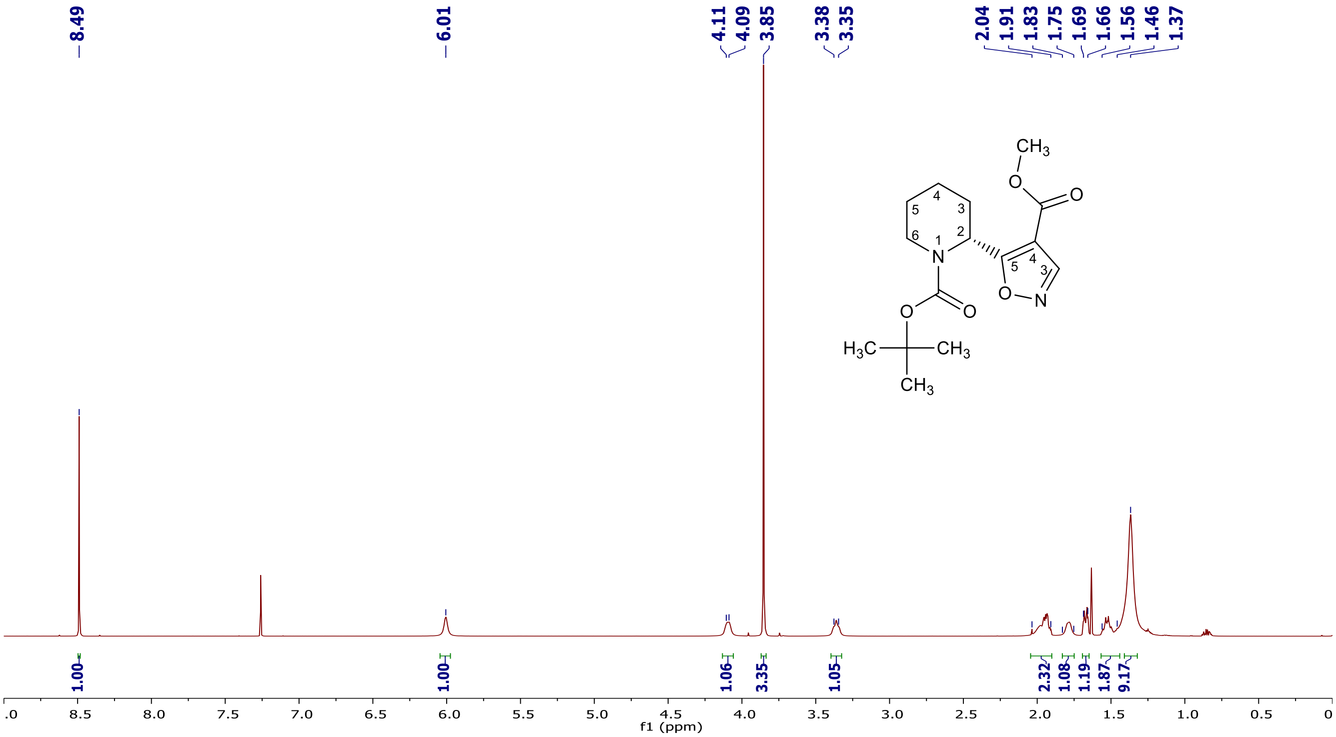

Figure S33: ^{1}H NMR of **4f**, ($CDCl_3$, 400 MHz).

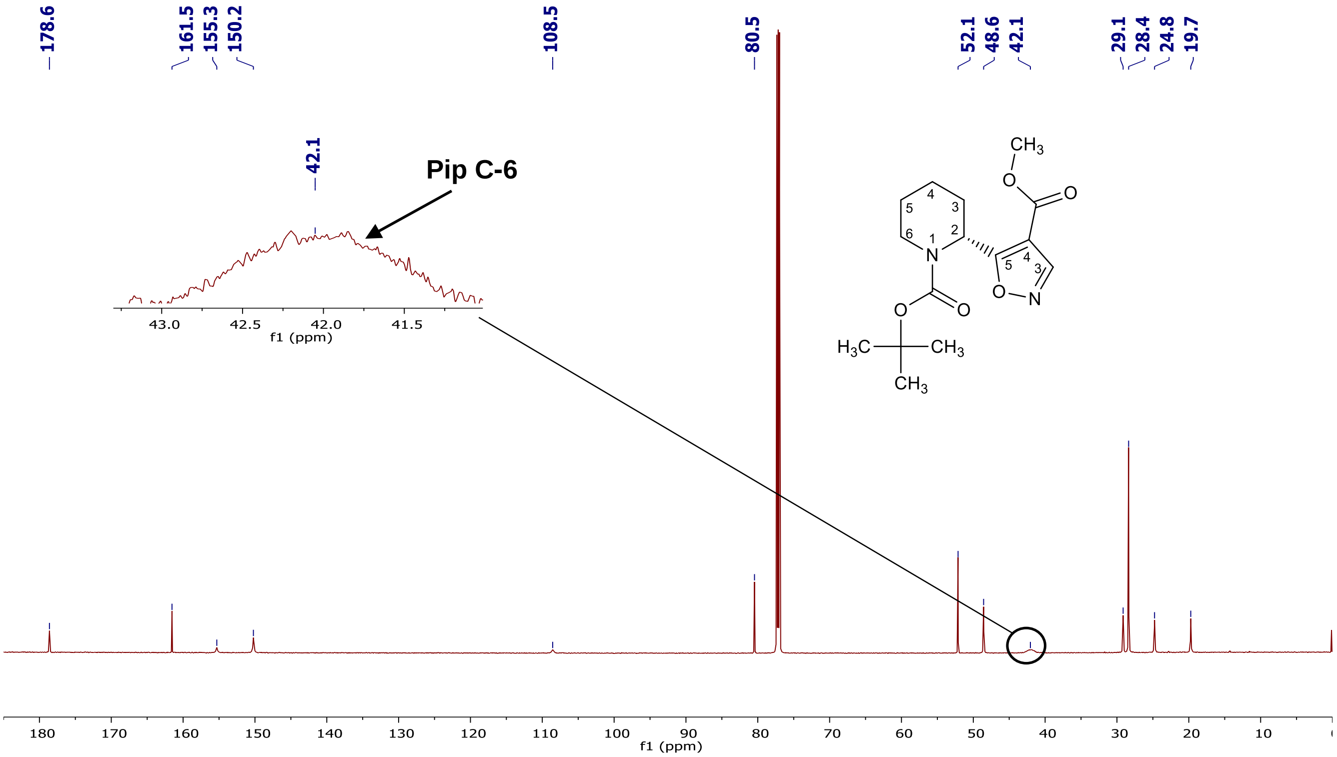

Figure S34: ^{13}C NMR of **4f**, ($CDCl_3$, 100 MHz).

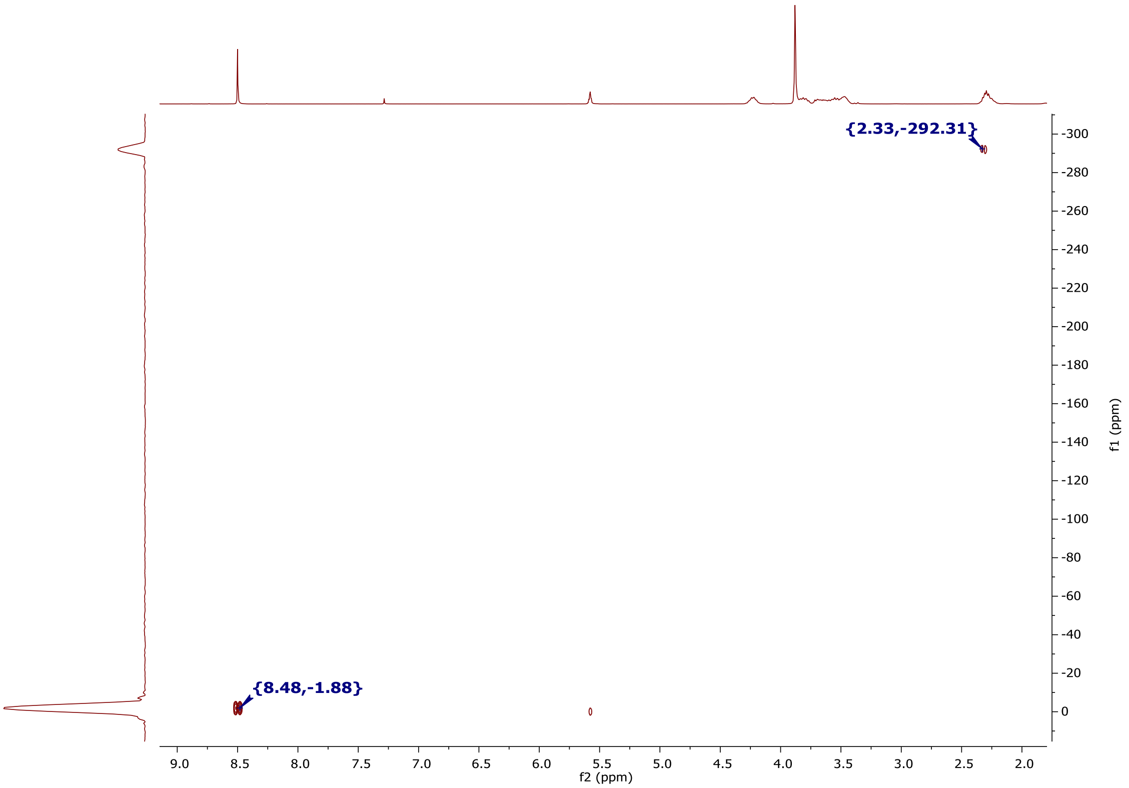

Figure S35: ^{15}N NMR of **4f**, ($CDCl_3$, 41 MHz).

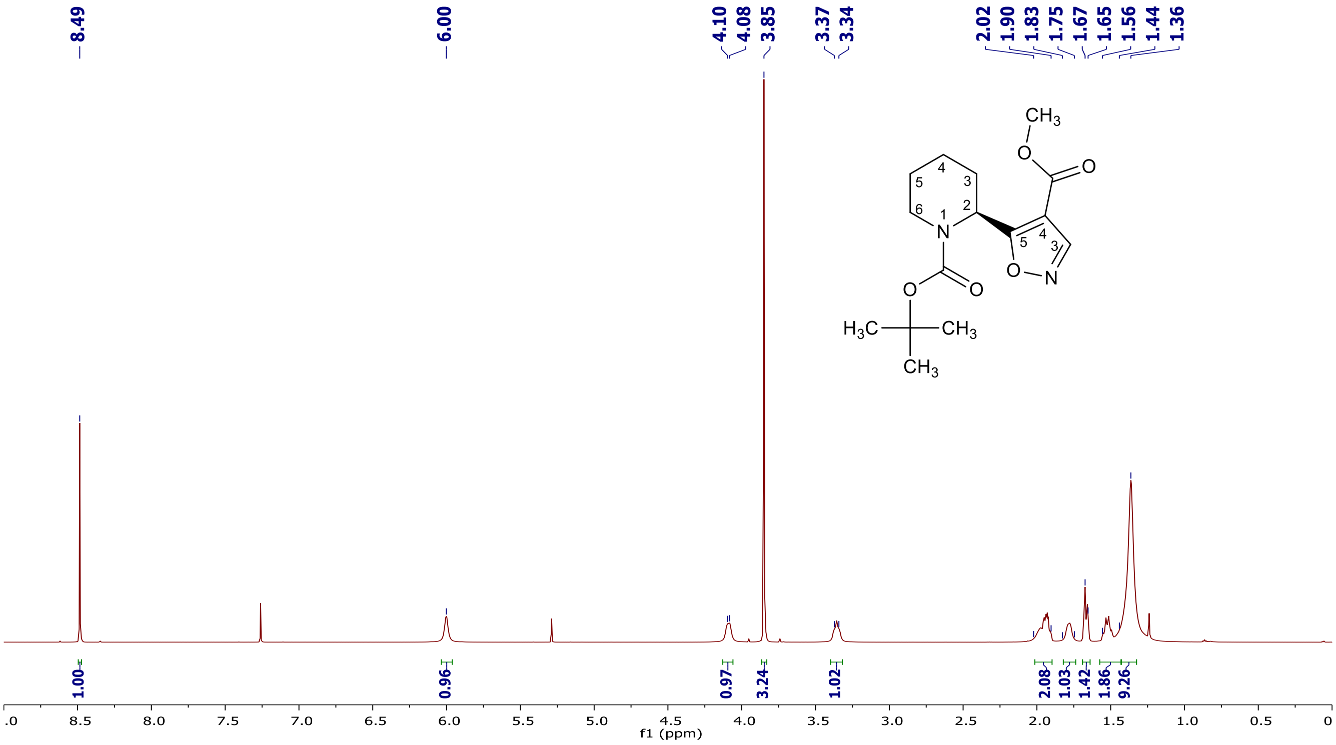

Figure S36: ^{1}H NMR of **4g**, ($CDCl_3$, 400 MHz).

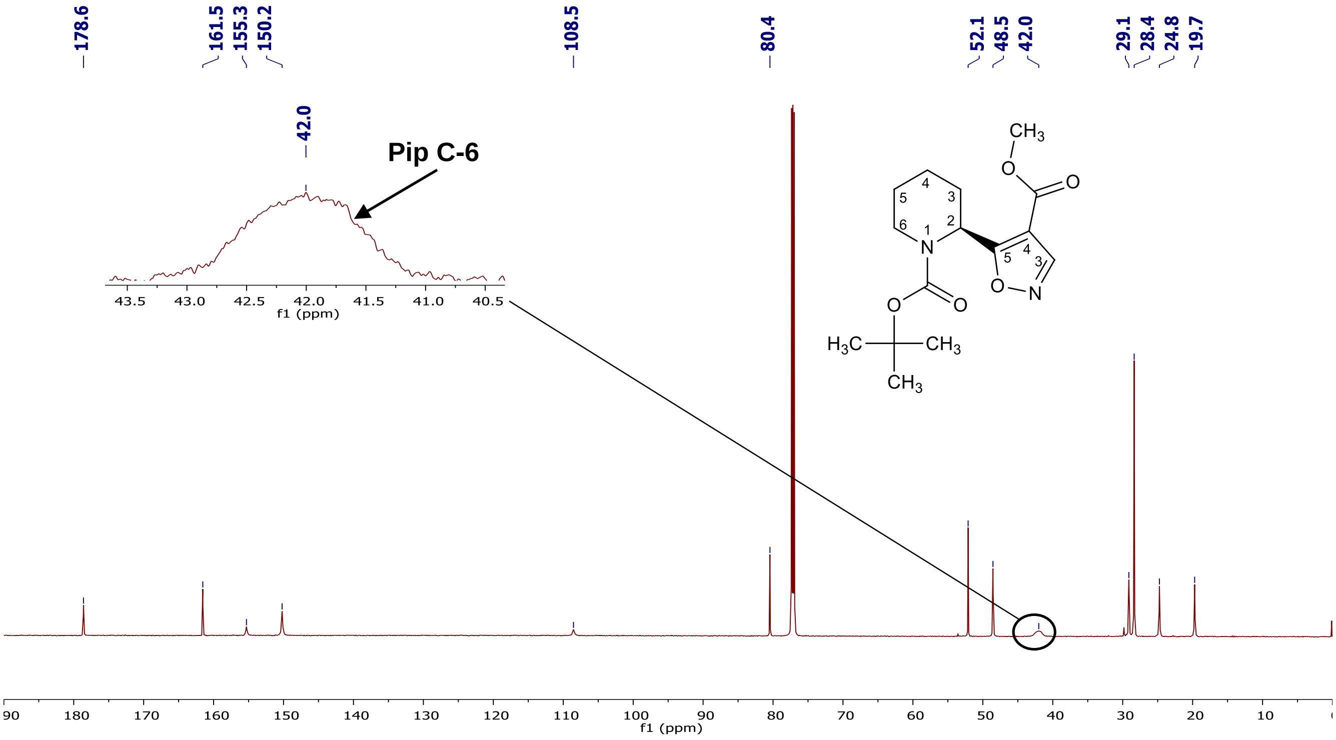

Figure S37: ^{13}C NMR of **4g**, ($CDCl_3$, 100 MHz).

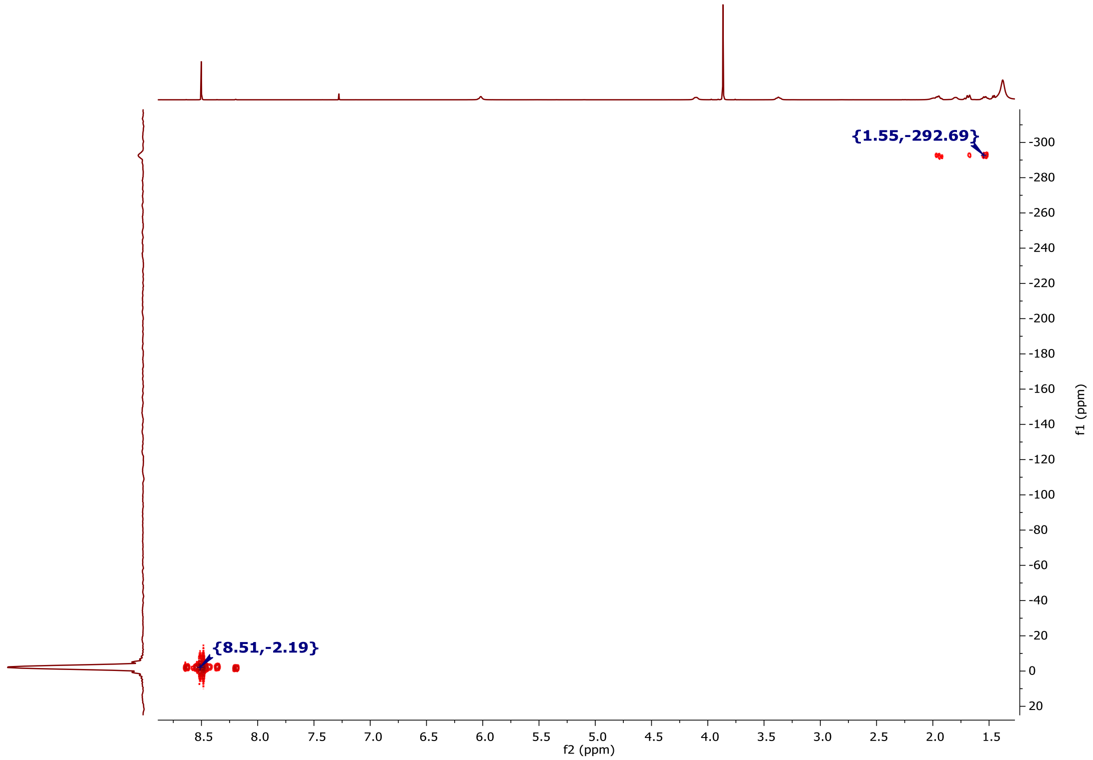

Figure S38: ^{15}N NMR of **4g**, ($CDCl_3$, 71 MHz).

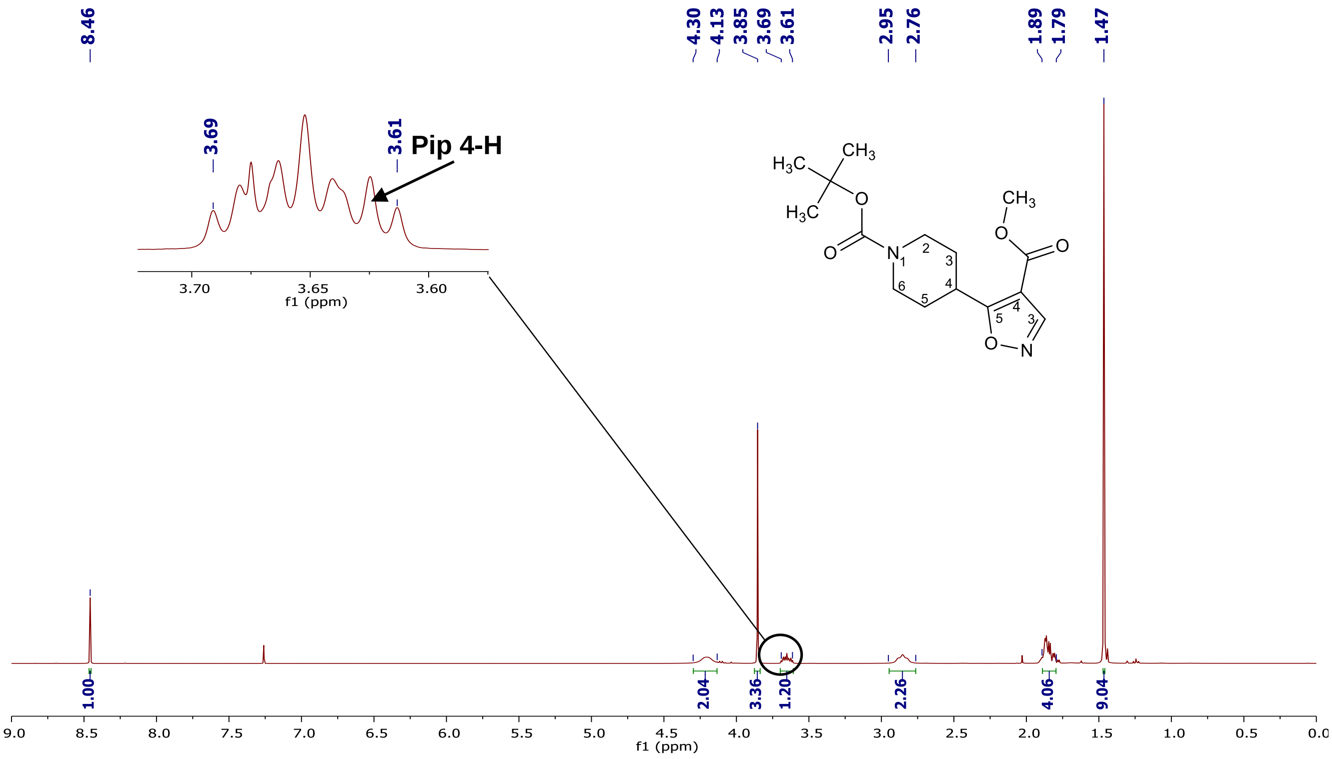

Figure S39: ^{1}H NMR of **4h**, ($CDCl_3$, 400 MHz).

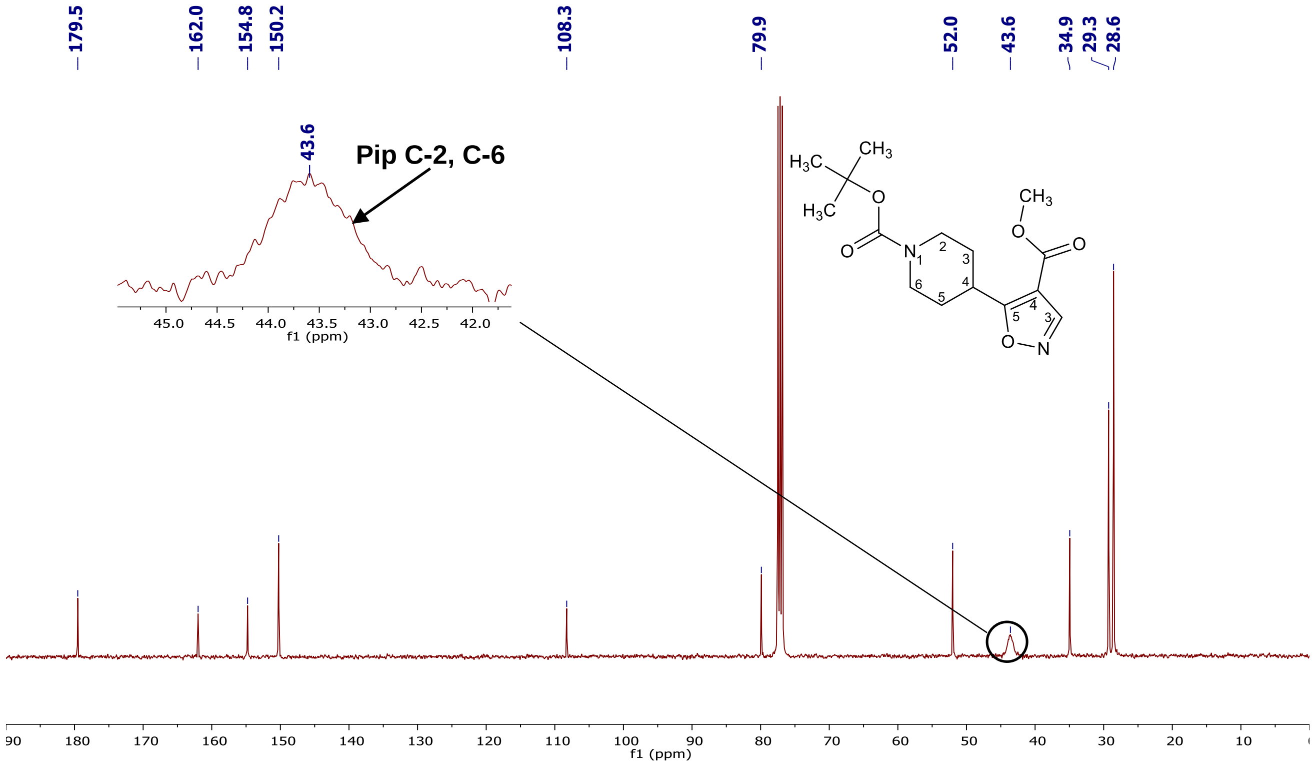

Figure S40: ^{13}C NMR of **4h**, ($CDCl_3$, 100 MHz).

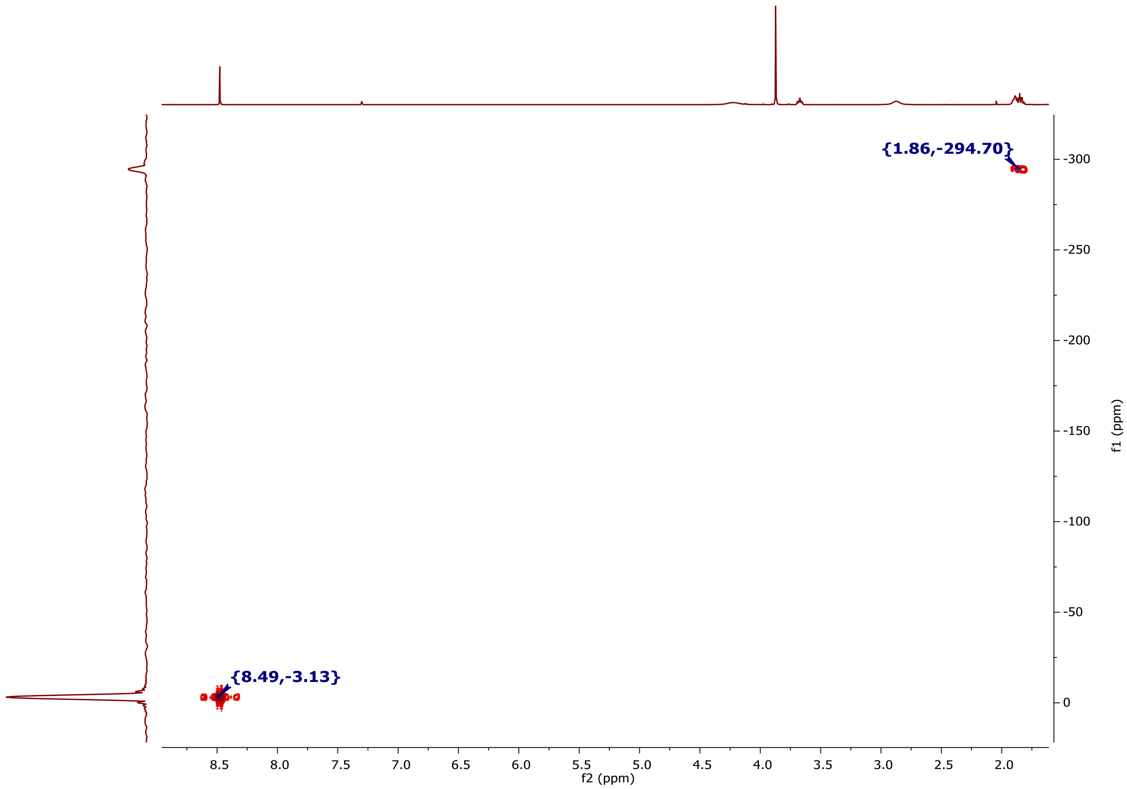

Figure S41: ^{15}N NMR of **4h**, ($CDCl_3$, 71 MHz).

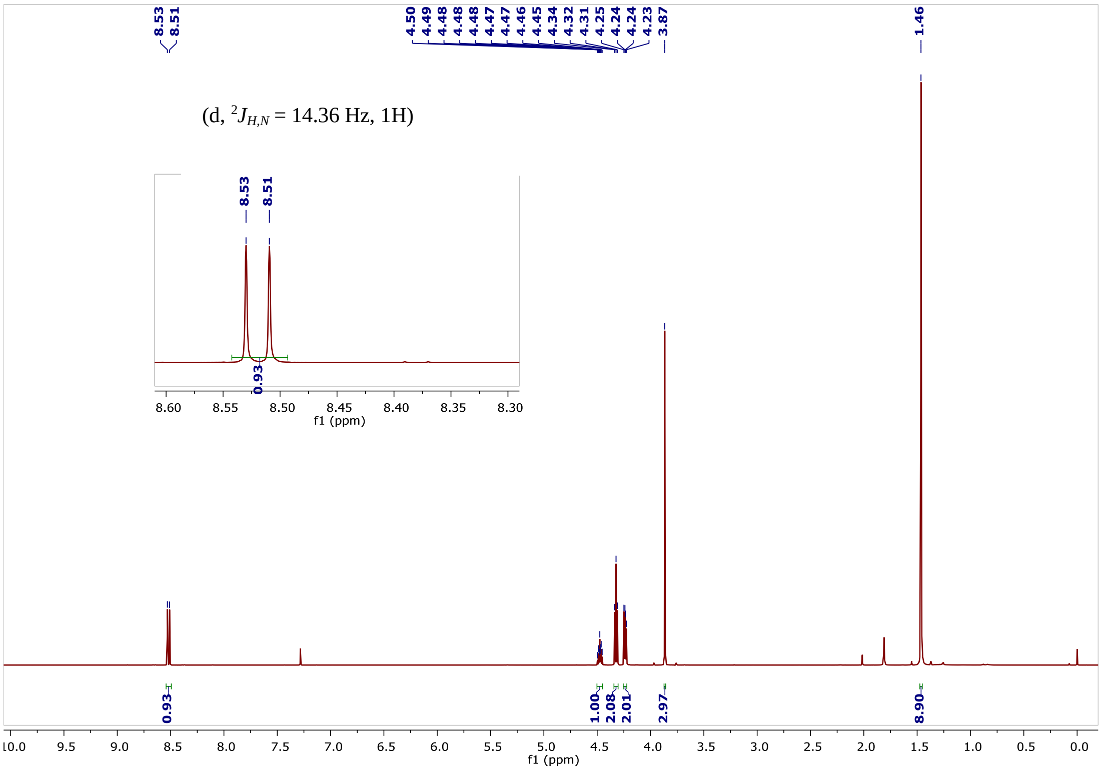

Figure S42: ^{1}H NMR of **5**, ($CDCl_3$, 700 MHz).

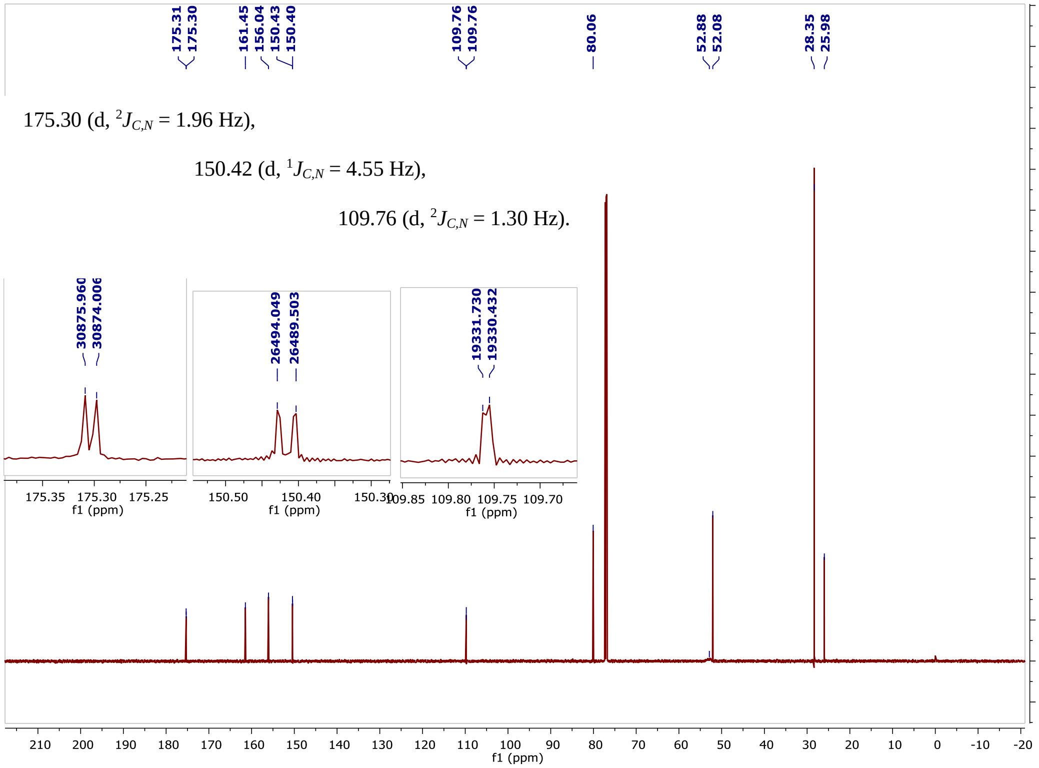

Figure S43: ^{13}C NMR of **5**, ($CDCl_3$, 176 MHz).

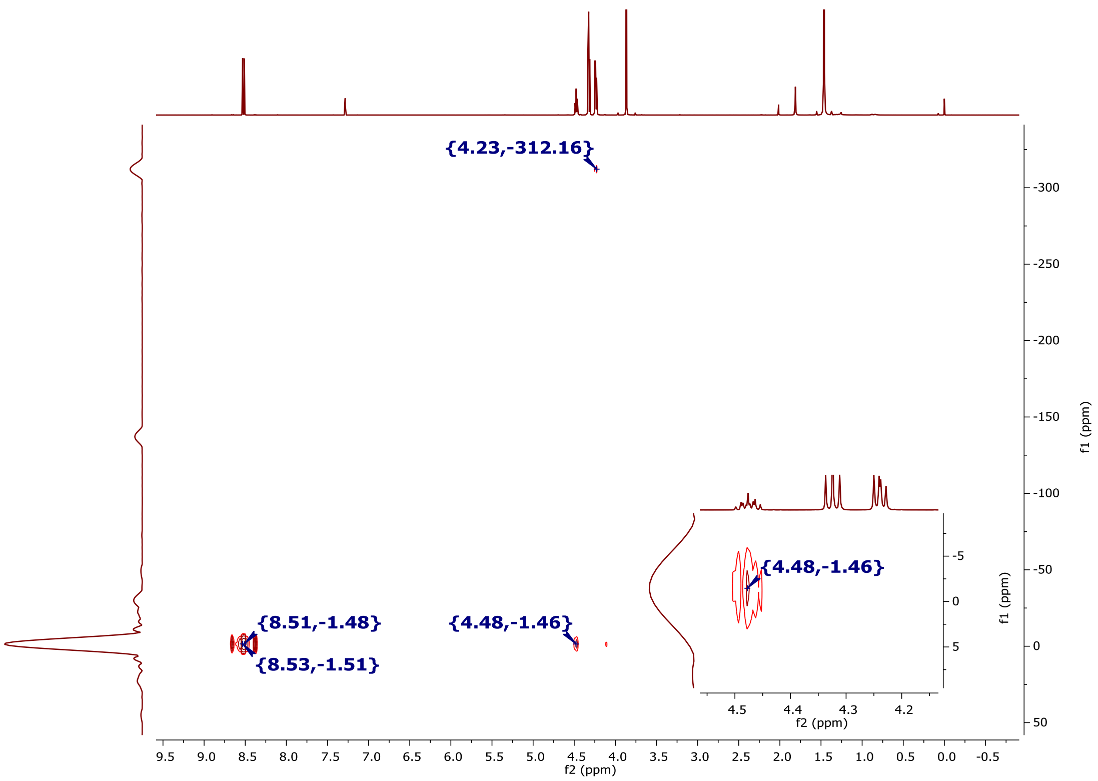

Figure S44: ^{15}N NMR of **5**, ($CDCl_3$, 71 MHz).

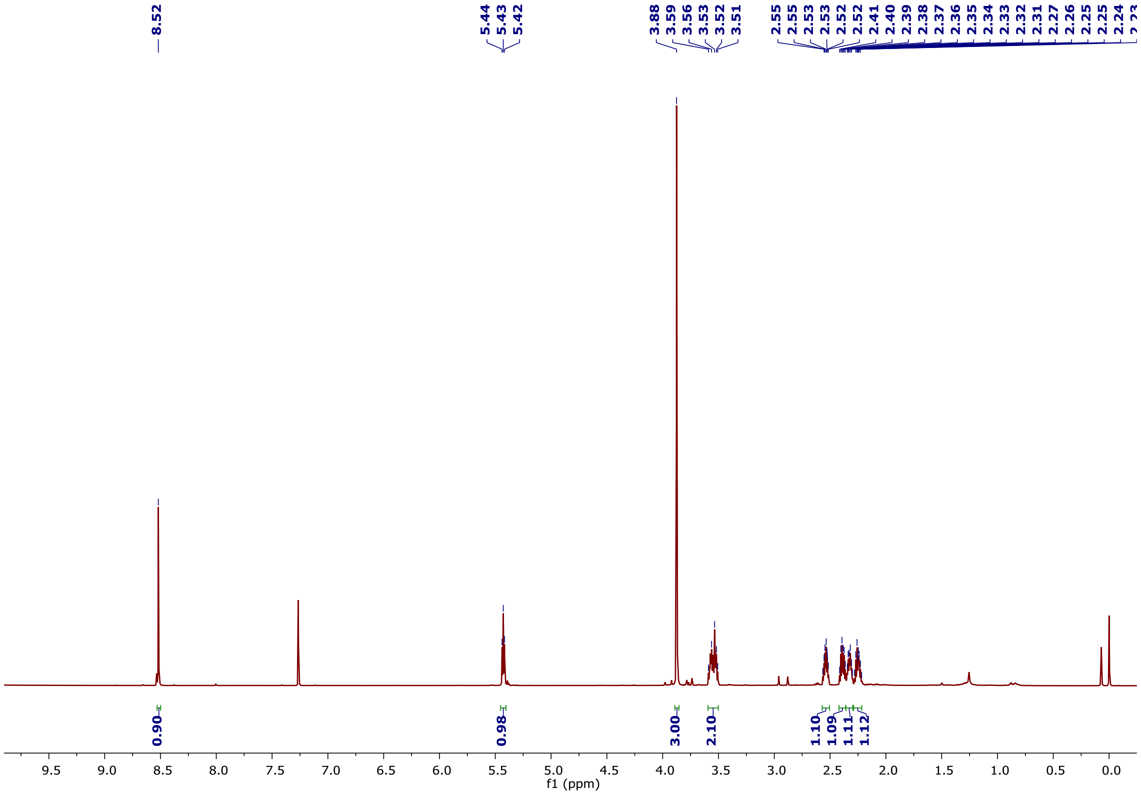

Figure S45: ^{1}H NMR of **6a**, ($CDCl_3$, 700 MHz).

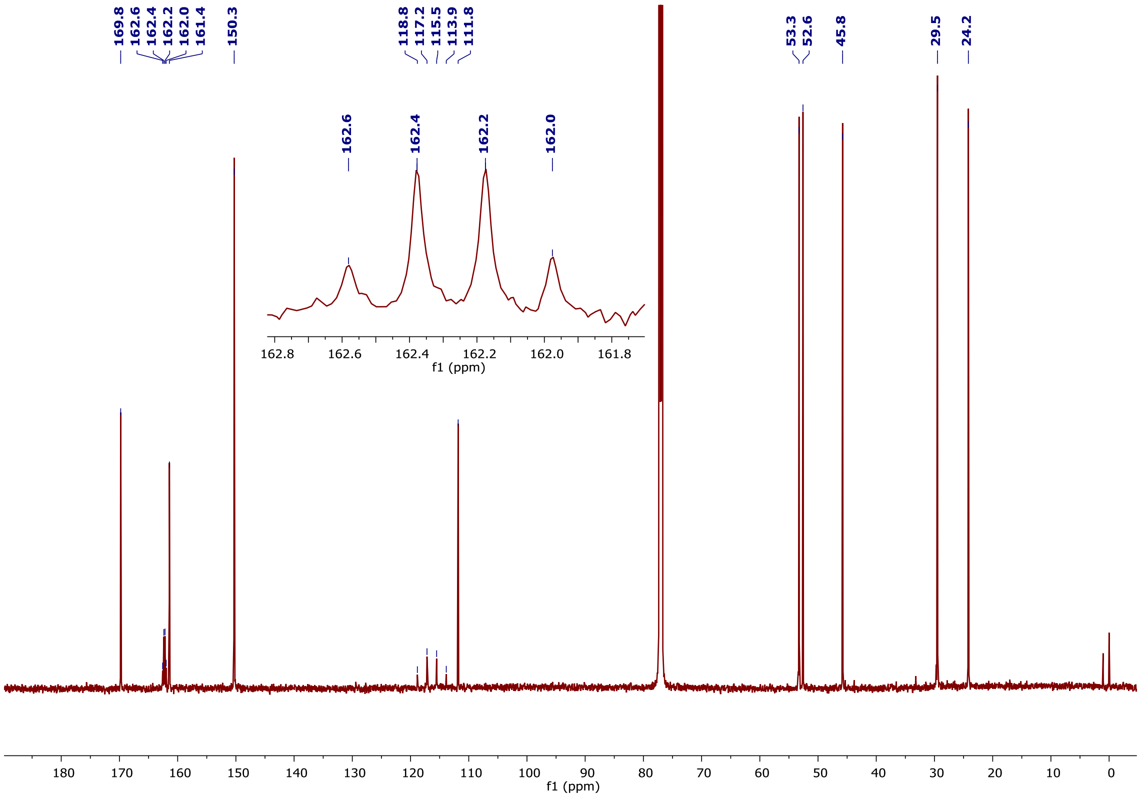

Figure S46: ^{13}C NMR of **6a**, ($CDCl_3$, 176 MHz).

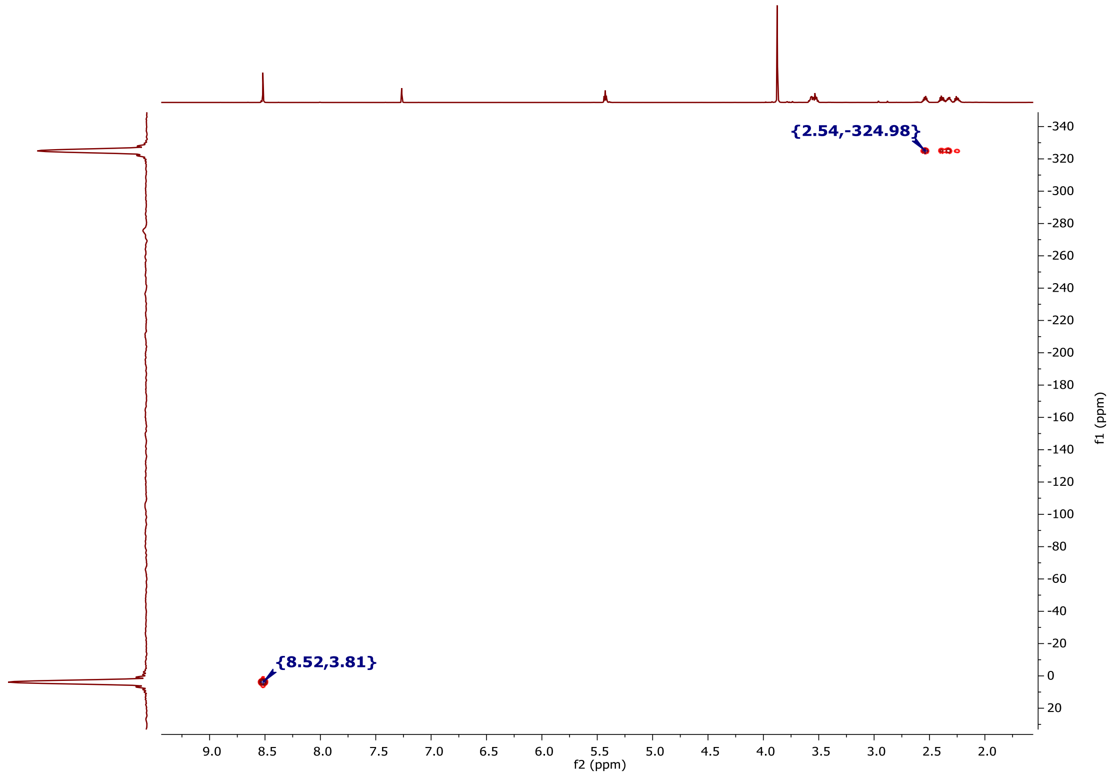

Figure S47: ^{15}N NMR of **6a**, ($CDCl_3$, 71 MHz).

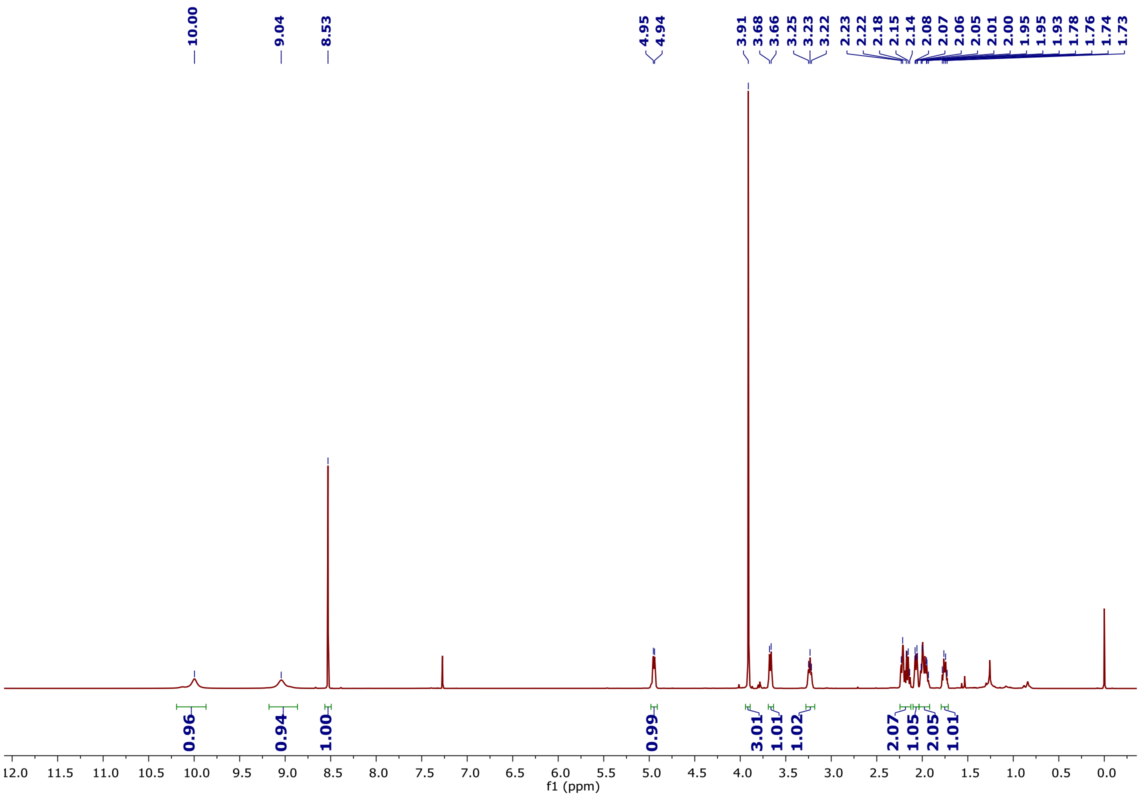

Figure S48: ^{1}H NMR of **6b**, ($CDCl_3$, 700 MHz).

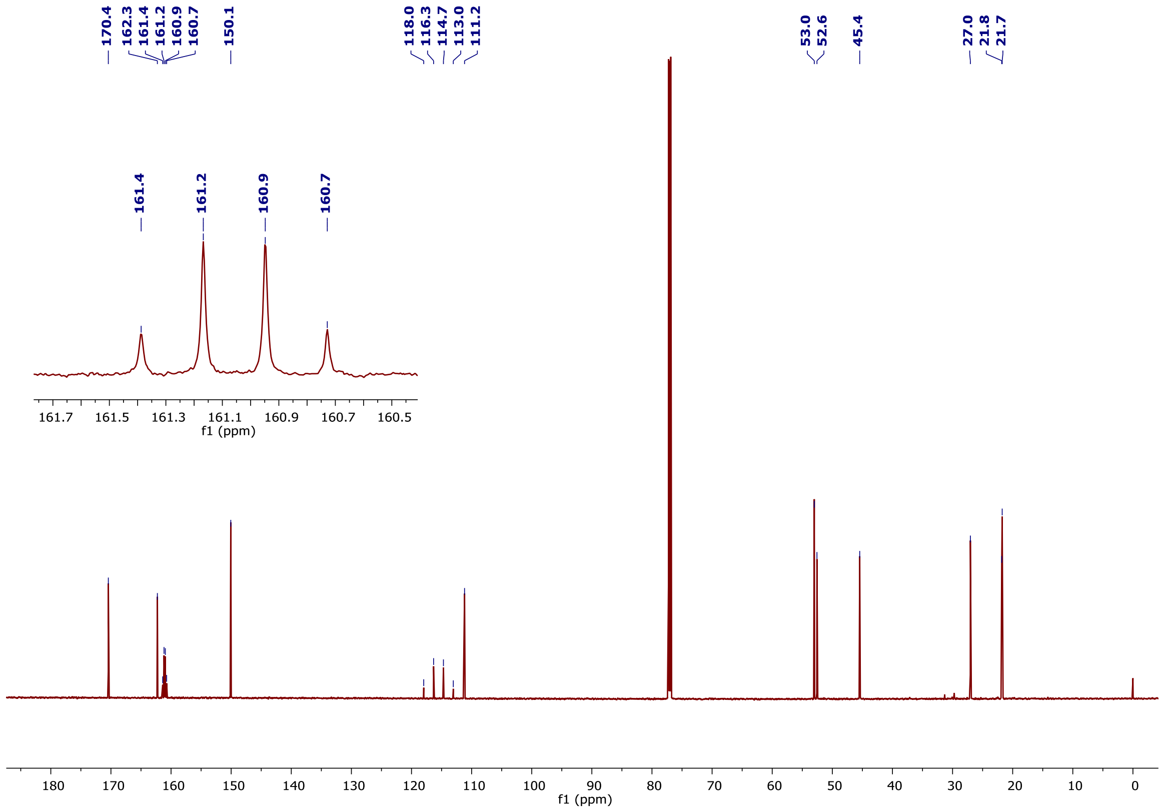

Figure S49: ^{13}C NMR of **6b**, ($CDCl_3$, 176 MHz).

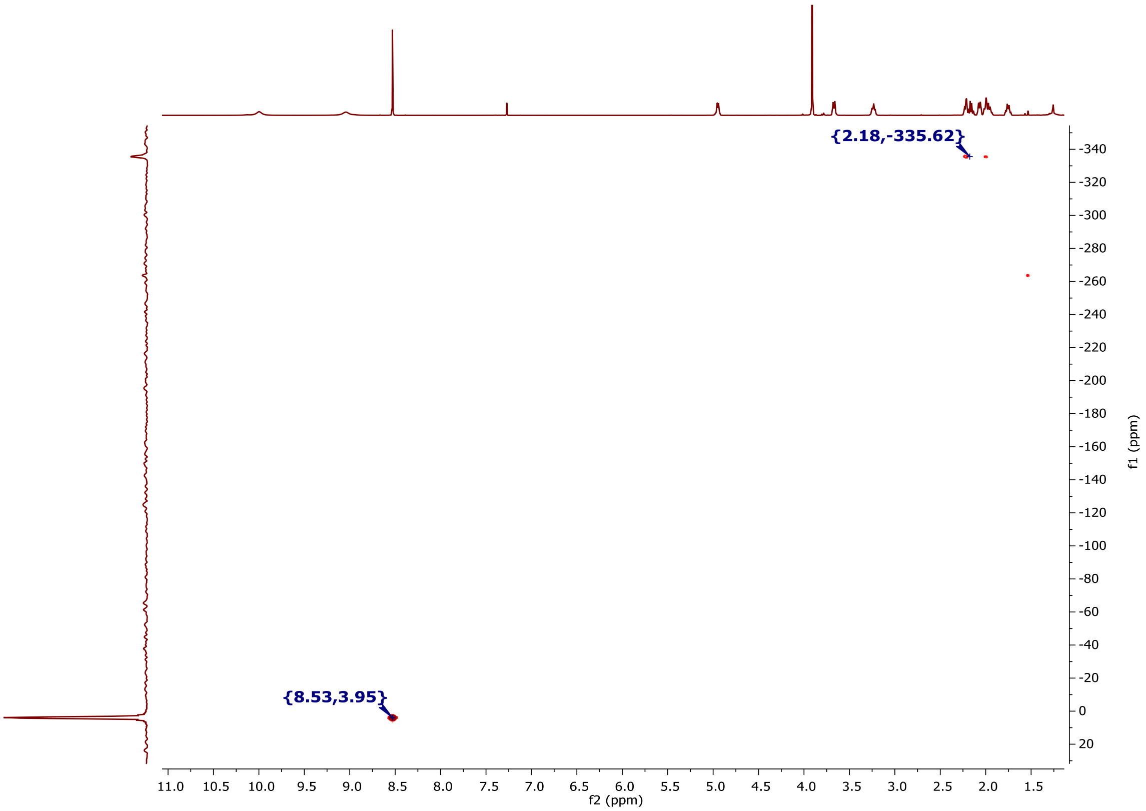

Figure S50: ^{15}N NMR of **6b**, ($CDCl_3$, 71 MHz).

7. Stacked chromatogram profile view of isolated enantiomers 4d–g

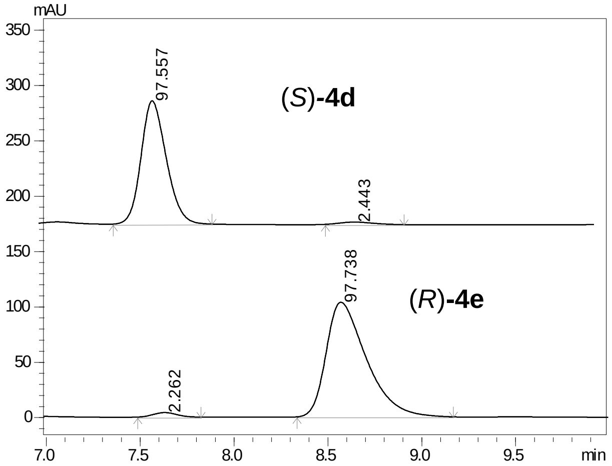

Figure S51: Stacked chromatogram profile view of isolated enantiomers **4d** and **4e**: (*S*)-**4d,** ee 98%**,** (t_R = 7.65 min) and (*R*)-**4e,** ee 98% (t_R = 8.6 min).

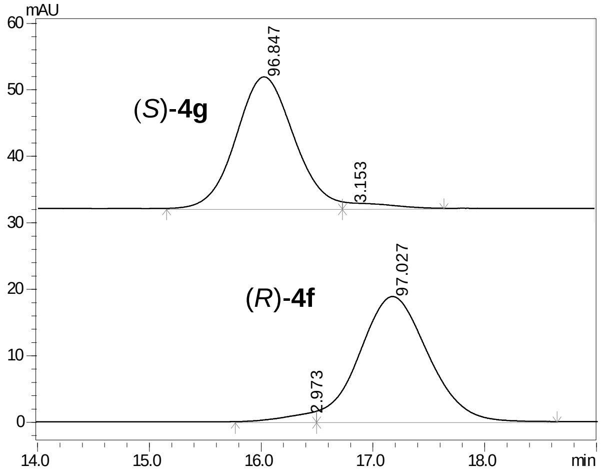

Figure S52: Stacked chromatogram profile view of isolated enantiomers **4f** and **4g**: (*R*)-**4f,** ee 97%**,** (t_R = 17.18 min) and (*S*)-**4g,** ee 97% (t_R = 16.08 min).

8. The ^{1}H NMR and 1D gradient NOE spectra of compound 4b

a)

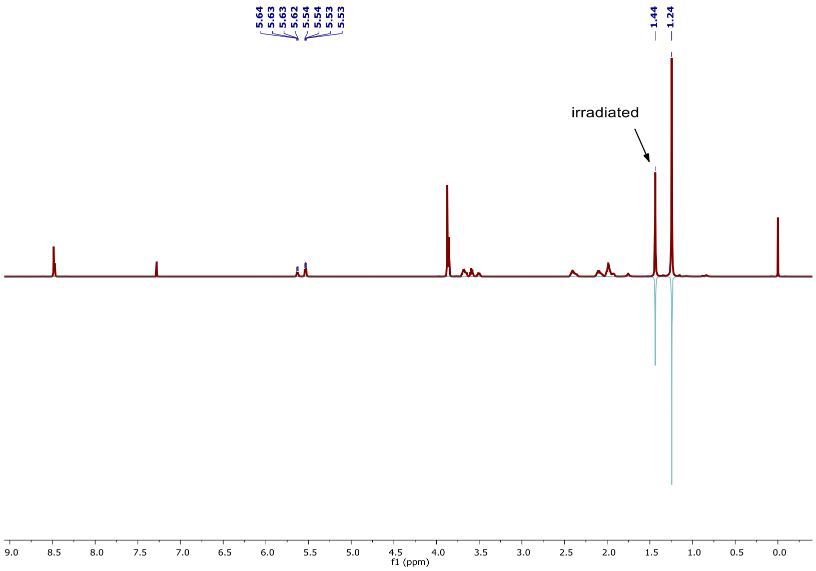

b)

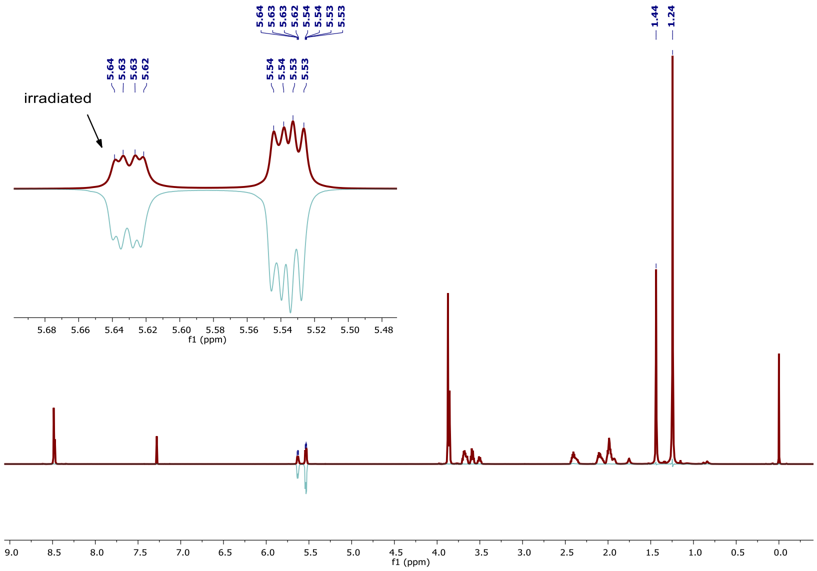

Figure S53: The superimposed ^{1}H NMR and 1D gradient NOE spectra with selective irradiation of signal at δ 1.44 ppm (a) and δ 5.61–5.65 ppm (b).

9. X-ray analysis of compound 6b

The X-ray intensity data was measured on a Rigaku, XtaLAB Synergy, Dualflex, HyPix diffractometer. Cu K\α micro focus sealed X-ray tube and PhotonJet (Cu) X-ray source. The structure was solved by direct methods and refined by full-matrix least squares techniques. Non-hydrogen atoms were refined with anisotropic displacement parameters. Hydrogen atoms were fitted to the peaks of the difference synthesis as well as calculated geometrically and refined with a riding model. The following software was used: CrysAlisPro (Rigaku corporation, 2020), Olex2 [1], the structure was solved with the ShelXT [2] structure solution program using Intrinsic Phasing and refined with the olex2.refine [3] refinement package using Gauss-Newton minimization. Experimental data and CCDC-code can be found in Table S1. Crystal data, data collection parameters, and structure refinement details are given in Tables S2 and S3. Molecular structure of asymmetric unit of **6b** as “Ortep View” is displayed in Figure S37.

Table S1: Experimental parameters and CCDC-Code

**Sample**	**Machine**	**Source**	**Temp.**	**Detector distance**	**Time/Frame**	**Fra-mes**	**Frame width**	**CCDC**
			[K]	[mm]	[s]		[°]	
5b	Rigaku, XtaLAB Synergy, Dualflex, HyPix	micro-focus sealed X-ray tube, PhotonJet (Cu)	150	31.4	0.33	2692	0.50	2003749

Table S2: Sample and crystal data of compound **6b**.

**Chemical formula**	$C_{10}H_{15}N_2O_3 \cdot C_2F_3O_2$	**Crystal system**	Monoclinic	
**Formula weight [g/mol]**	324.26	**Space group**	*P2₁*	
**Temperature [K]**	150	**Z**	4	
**Measurement method**	ϕ and ω scans	**Volume [Å^3]**	1422.77 (3)	
**Radiation wavelength [Å]**	1.54184	**Unit cell dimensions [Å^3] and [°]**	9.1009 (1)<br>17.8668 (1)<br>9.8366 (1)	90<br>117.186 (2)<br>90
**Crystal size/ [mm^3]**	0.22 × 0.18 × 0.17			
**Crystal habit**	Block, pale yellow			
**Density (calculated)/ [g/cm^3]**	1.514	**Absorption coefficient [mm^{-1}]**	1.25	
**Abs. Correction T_{min}**	0.746	**Abs. Correction T_{max}**	0.867	
**Abs. Correction type**	multi-scan *CrysAlis PRO* 1.171.40.35a (Rigaku Oxford Diffraction, 2018) Empirical absorption correction using spherical harmonics, implemented in SCALE3 ABSPACK scaling algorithm.	**F(000) [e$^-$]**	672	

Table S3: Data collection and structure refinement of compound **6b**.

**Index ranges**	-11 ≤ h ≤ 11<br>-22≤ k ≤ 14	**Theta range for data collection**	5-78

	-12 ≤ l ≤ 12	**[°]**		
**Reflections numbers**	4362	**Data / restraints / parameters**	4362/1/415	
**Refinement method**	Least squares matrix: full	**Final R indices**	All data	R1 = 0.0384; wR2 = 0.0992
**Function minimized**	$\Sigma w[\vert Fo\vert^2 - (1/k)\vert Fc\vert^2]$		**I** > 2σ(**I**)	R1 = 0.0382; wR2 = 0.0989
**Goodness-of-fit on F2**	1.0658	**Weighting scheme**	$w = 1/[\sigma^2(F_o^2) + (0.0678P)^2 + 0.2756P]$ where $P = (F_o^2 + 2F_c^2)/3$	
**Largest diff. peak and hole [e Å-3]**	+0.255 and -0.285			

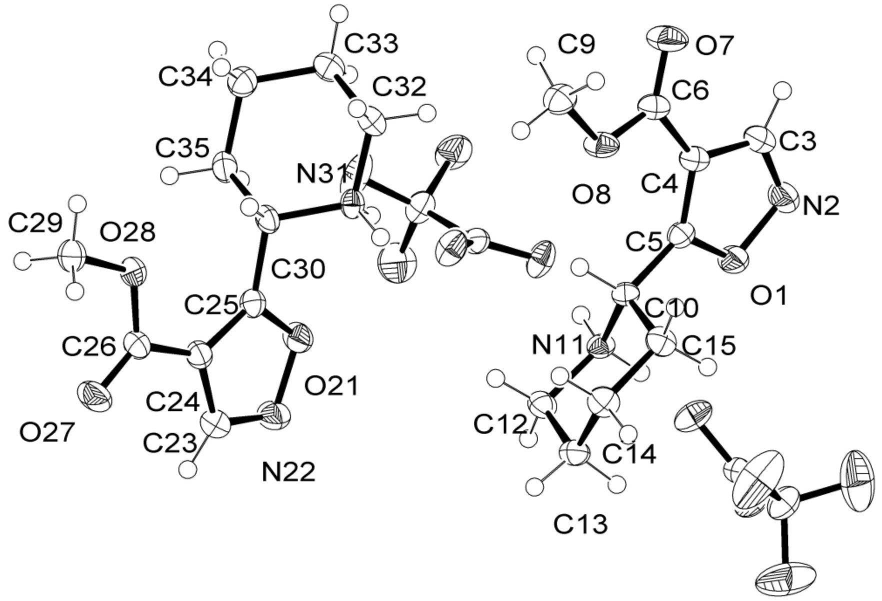

Figure S54: ORTEP view of asymmetric unit of crystal **6b**.

10. References

1. Dolomanov, O. V.; Bourhis, L. J.; Gildea, R. J.; Howard, J .A. K.; Puschmann, H. *J. Appl. Cryst*. **2009**, *42*, 339-341. doi:10.1107/S0021889808042726
2. Sheldrick, G. M. *Acta Cryst*. *A*, **2015**, *A71*, 3-8. doi:10.1107/S2053273314026370
3. Bourhis, L. J.; Dolomanov, O. V.; Gildea, R. J.; Howard, J. A. K.; Puschmann, H. *Acta Cryst*. *A*, **2015**, *A71*, 59-75. doi:10.1107/S2053273314022207
4. Dzedulionytė, K.; Voznikaitė, P.; Bieliauskas, A.; Malinauskienė, V.; Sløk, F. A.; Šačkus, A. *Molbank* **2021**, *2021*, M1207. https://doi.org/10.3390/M1207
5. Greck, C.; Thomassigny, C.; Le Bouc, G. *Arkivo*c **2012**, *8*, 231–249. doi:10.3998/ark.5550190.0013.821
6. Malinauskienė, V.; Kveselytė, A.; Dzedulionytė, K.; Bieliauskas, A.; Sløk, A. F.; Šačkus, A. *Chem. Heterocycl. Compd.* **2018**, *54,* 469–473. doi:10.1007/s10593-018-2291-1
7. Gao, W.; Lau, T.; Pan, S.; Phillips, D. P.; Wang, X. Compounds and compositions as TGR5 agonists. WO Patent 2012/082947A1, June 21, 2012.
8. Matulevičiūtė, G.; Arbačiauskienė, E.;, Kleizienė, N.; Kederienė, V.; Ragaitė, G; Dagilienė, M.; Bieliauskas, A.; Milišiūnaitė, V.; Sløk, F.A.; Šačkus, A. *Molecules* **2021**, *26*, 3808. doi:10.3390/molecules26133808
